# Pincer Nickel(II) Catalyzed Oxidative Carbonylation of Amines: A Phosgene-Free Synthesis for Isocyanates and Ureas

**DOI:** 10.1021/acsomega.6c01401

**Published:** 2026-06-08

**Authors:** Peter Szwedo, Raja Shekhar Kondrapolu, Pradip Munshi, Anindya Ghosh

**Affiliations:** † School of Physical Sciences, Chemistry Program, 14658 University of Arkansas at Little Rock, 2801 South University Avenue, Little Rock, Arkansas 72204, United States; ‡ CISCHEM (Centre for Innovation and Sustainable Chemistry), FF21 Kalpvruksh Complex, Vadodara, Gujarat 390021, India

## Abstract

A novel phosgene-free process for isocyanate and urea formations from amines and cyclic carbonates catalyzed via a Nickel­(II) pincer complex (**2**) is demonstrated. Under relatively mild reaction conditions (2 h and 25 °C for isocyanates; 3 h and 100 °C), various aliphatic and substituted aromatic amines are converted into 32 different isocyanate and urea products. Upon optimization, moderate to good turnover numbers (TONs) for isocyanate formations were calculated, with the highest TON for (3-isocyanatopropyl) benzene (4d, TON 277, 55% yield). For ureas, moderate to good TONs were also observed, with the highest TON for 1,3-bis­(3-phenylpropyl) urea (6g, TON 346, 69% yield). Kinetic studies were performed by varying the amounts of amine, base, catalyst, and propylene carbonate to determine the order of each component and establish the overall order as 2. With the help of kinetic data and analytical results, a possible reaction pathway is proposed through a Nickel­(I)–Nickel­(III) pathway.

## Graphical abstract

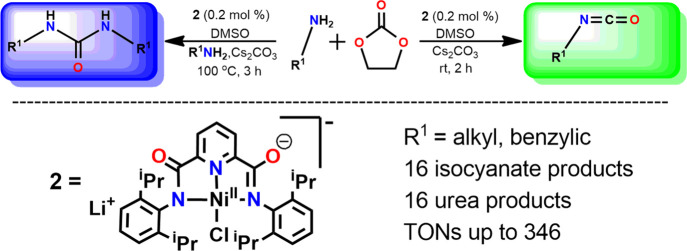

## 1 Introduction

Isocyanates are versatile synthetic intermediates for highly sought-after C–N containing organic compounds and polymeric materials.^1,2^ Due to their unique electrophilic nature,^3^ functionally modified isocyanates can react with a broad range of alcohols and amines to produce a variety of significant chemicals such as carbamates and ureas.^4–6^ Diisocyanates are another important set of isocyanates, primarily known as precursors for polyurethanes.^7–9^ Further, isocyanates are used in industrial production of materials such as elastomers, insulation, paints, and protective coatings.^10^ Due to demand for isocyanates, the global market for them grows by 5% each year, with primary demand in polyurethane production.^11^ Ureas are another class of C–N containing compounds that serve as a key basic raw chemical.^12^ Ureas are naturally occurring^13^ but urea moieties are present in industrially significant chemicals such as agrochemicals, dyes, pharmaceuticals, and resins_._ ^14–16^ Some key compounds featuring isocyanate or urea groups are illustrated in Figure 1.

**Figure 1:**
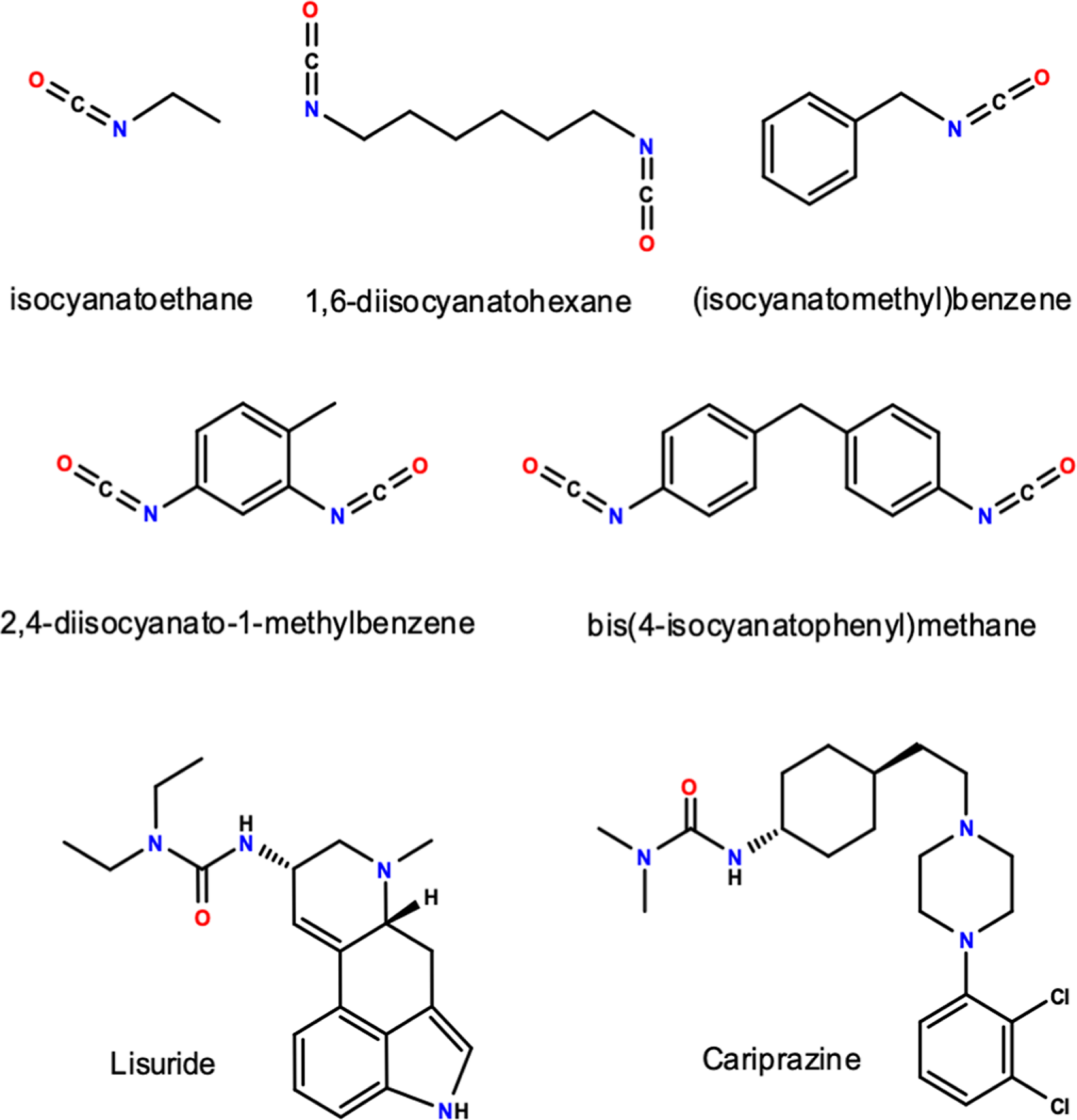
Important selected compounds containing the isocyanate and urea.

Despite the high demand and utilization for isocyanate and urea compounds, challenges remain in the synthetic processes.^17,18^ Presently, industrial isocyanate production relies almost entirely on the reaction of primary amines with phosgene.^19–22^ However, phosgene is a poisonous gas and has been classified as a chemical weapon after being the most lethal chemical agent in World War I.^23,24^ Further, the highly exothermic synthesis of phosgene involves the use of hazardous carbon monoxide, chlorine, and hydrochloric acid, which is produced as a byproduct.^25^ Replacement of phosgene for the synthesis of isocyanates has been successfully achieved utilizing carbonyldiimadizole (CDI); ironically, however, phosgene is also a critical component in producing CDI.^26^ Curtis, Hoffman, and Lossen rearrangements are commonly used to produce isocyanates in lab settings, but the Curtis and Hoffman rearrangements are toxic and involve halogenated species throughout the process.^27–30^ Boc-anhydrides and primary amines, along with leaving group displacement with metal cyanates, have been reported to generate the isocyanate from primary amines.^31,32^ Phosgene-free isocyanate production through reductive carbonylation of CO from nitro-arene compounds^33^ has been known since 1967, and developing these processes has been an area of interest among researchers. Additionally, carbamates and ureas can be thermally induced to reform the original isocyanate product.^10^ Table 1 shows a few examples of nonphosgene pathways to generate isocyanates including the current study.

**Table 1:** Comparison of the Non-Phosgene Route to Isocyanate and Urea Synthesis

	starting material	condition	yield	ref
1	R-NH-BOC	2-Cl-Pyridine (base), Trifluoromethanesulfonylanhydride (1.5 equiv), isobutanol (3 eq), 1 h, RT	Isocyanate (in situ formed).	^31^
			Carbamate 89%	
2	*n*Bu-Cl	NaOCN, Ni(dppe)_2_, DPPE, DMAC, 4.5 h, 150 °C	Butyl isocyanate 22%	^32^
3	Ar–NO_2_	CO (200 bar), Rh/C, FeCl_3_, 1,1,2-trichloro-1,2,2-trifluoro ethane, 190 °C, 5.5 h	Aryl isocyanate 35%	^33^
4	Ar–Cl or Ar-Triflate + NaOCN	Pd-Cat, Toluene, 110 °C, 16 h	Aryl isocyanate 86%	^47^
5	Cyanuric chloride + Ar–OH	Ni(COD)_2_/DPPE, KOCN, 100 °C, 2 h	Aryl isocyanate, 22%	^48^
6	R-NH–COOR^/^	ZnO, IL, 240 C, 1.5 h	RNHCO 85%	^64^
7	Aniline	Co-Salen/Silica, 40 bar CO + O_2_ (19:1 V), Dichlorobenzene, Trifluoroethanol, NaI 120 °C, 3 h	Phenyl isocyanate 51%	^66^
8	Ph(CH_2_)_3_I + CO + Na N_3_	40 W Blue LED, Ni/Pd(COD), Xanthphos (5 mol %), 48 h, Benz, MeCN	Ph(CH_2_)_3_NCO (90%)	^67^
9	Ph-(CH_2_)_3_NH_2_ + C_2_H_4_CO_3_(Primary amine + Ethylene carbonate)	Ni-Pincer (0.2mol %), CS_2_CO_3_, DMSO, RT, 2 h	3-isocyanatopropyl benzene, 55%	present work
10	CO_2_ + NH_3_	CuSO4 (50%), 25 MPa, 220 °C	NH_2_CONH_2_, 10%	^34^
11	R-NH_2_	CO, Fe, PdX_2_ (0.02 mol %), I_2_		^61^
12	Ph-(CH_2_)_3_NH_2_ + C_2_H_4_CO_3_(Primary amine + Ethylene carbonate)	Ni-Pincer (0.2mol %), CS_2_CO_3_, DMSO, 100 °C, 3 h	Ph(CH_2_)_3_NH–CO-NH(CH_2_)_3_Ph	present work

The primary method for simple urea synthesis involves variations of the Bosch-Meiser process, but it requires high-pressure, high-temperature conditions.^34,35^ However, designing complicated, substituted ureas is more challenging. CO_2_ fixation and production of ureas from CO_2_ and amines has been one such promising solution and a large area of focused research.^36–40^ On the surface, the process involves reacting an amine with CO_2_ to form carbamic acid, then reacting it with another amine to produce a carbamate salt, followed by dehydration to form a newly substituted urea product.^13^ However, the dehydration step is challenging, and the transformation steps among CO_2_ and amines are reversible.^13,41^ The main industrial processes for polyurea synthesis involve additions of amines to isocyanates^42^ and serve as another attractive route for complex synthetic urea productions.^43,44^

To devise a more environmentally friendly and cost-effective process, there’s interest in catalysts involving 3d metals.^45^ Metals like cobalt, iron, and nickel are not only more affordable but also essential for life, making them attractive alternatives to heavier 4d and 5d metals.^46^ Recent efforts have focused on transition-metal-catalyzed approaches using Cu, Ni, and Pd complexes for isocyanate formation and subsequent carbamate and urethane synthesis.^47–49^ Oxidative carbonylations of amines catalyzed by nickel and palladium,^45,50^ but these reactions still require elevated pressures or temperatures and remain vastly underexplored.^14^ Hence, innovating nonphosgene synthetic routes for isocyanate and urea synthesis holds substantial appeal for both academia and industry alike.^46,51^

In this study, we employed an amide-based NNN Nickel­(II) pincer complex **(2**) for isocyanate and urea synthesis through oxidative carbonylation of amines using propylene carbonate as the carbonyl source. Oxidative carbonylations of alkanes^52–56^ and alcohols to carbonates using carbon monoxide as the carbonyl source have been reported.^57^ Additionally, conversions of amines to ureas via oxidative carbonylation using carbon monoxide as carbonyl source have also been reported.^58–62^ More recently, oxidative carbonylation has been reported for the synthesis of drug molecules bearing unsymmetrical urea moieties using copper and cobalt catalysis.^63^ However, oxidative carbonylations of amines to isocyanate synthesis have been limited to theoretical study^64^ or to carbon monoxide or phosgene sources in several reports.^65–67^ Some processes using cyclic carbonates as starting materials for similar types of products have also been reported in the literature.^42,68,69^ The pathway for the formation of isocyanate and urea products from amines and cyclic carbonates, catalyzed by nickel­(II) pincer complex (**2**), described in present study, is the first of its kind to the best of our knowledge.

Though the nickel­(II) pincer catalyst (**2**) used in this study has already introduced new methods for C–C and various C–N bond formations,^70–74^ this catalyst (**2**) has not yet been explored for the synthesis of isocyanates and ureas as shown in Figure 2 below. The methodology has huge potential toward nonphosphene routes of synthesis for isocyanate and urea.^75^ The present report highlights a one-pot process that converts cyclic carbonates and amines to isocyanates and urea derivatives with satisfactory yields, achieving turnover numbers (TONs) up to 276 for isocyanates and 346 for ureas (Schemes 2 and 4). The isocyanate reactions occur at room temperature with a base for 2 h, while urea reactions require higher temperatures (100 °C) with a base in 3 h. below.

**Figure 2:**
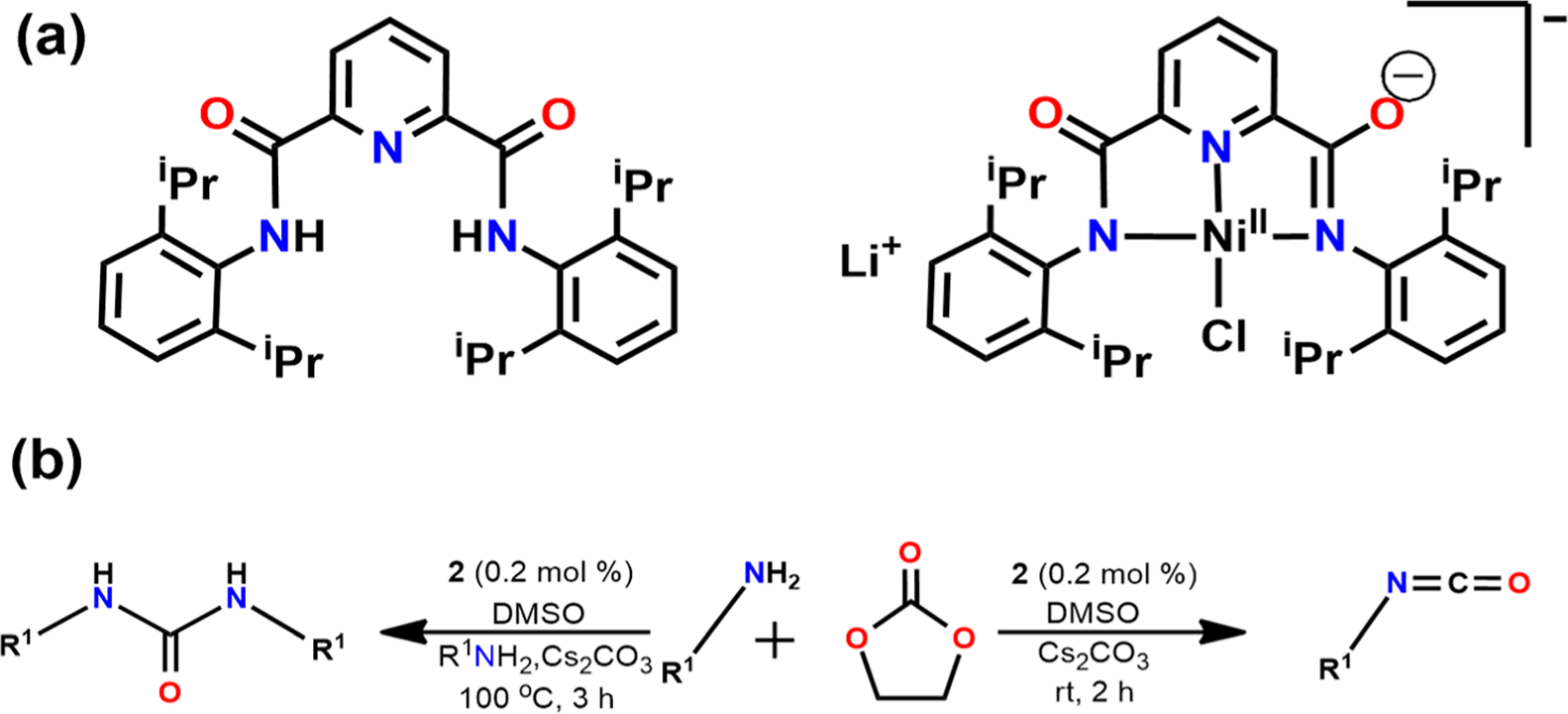
(a) Design of pincer ligand (**1**) and nickel­(II) pincer complex (**2**). (b) The general reaction for isocyanates and ureas.

**Scheme 1:**
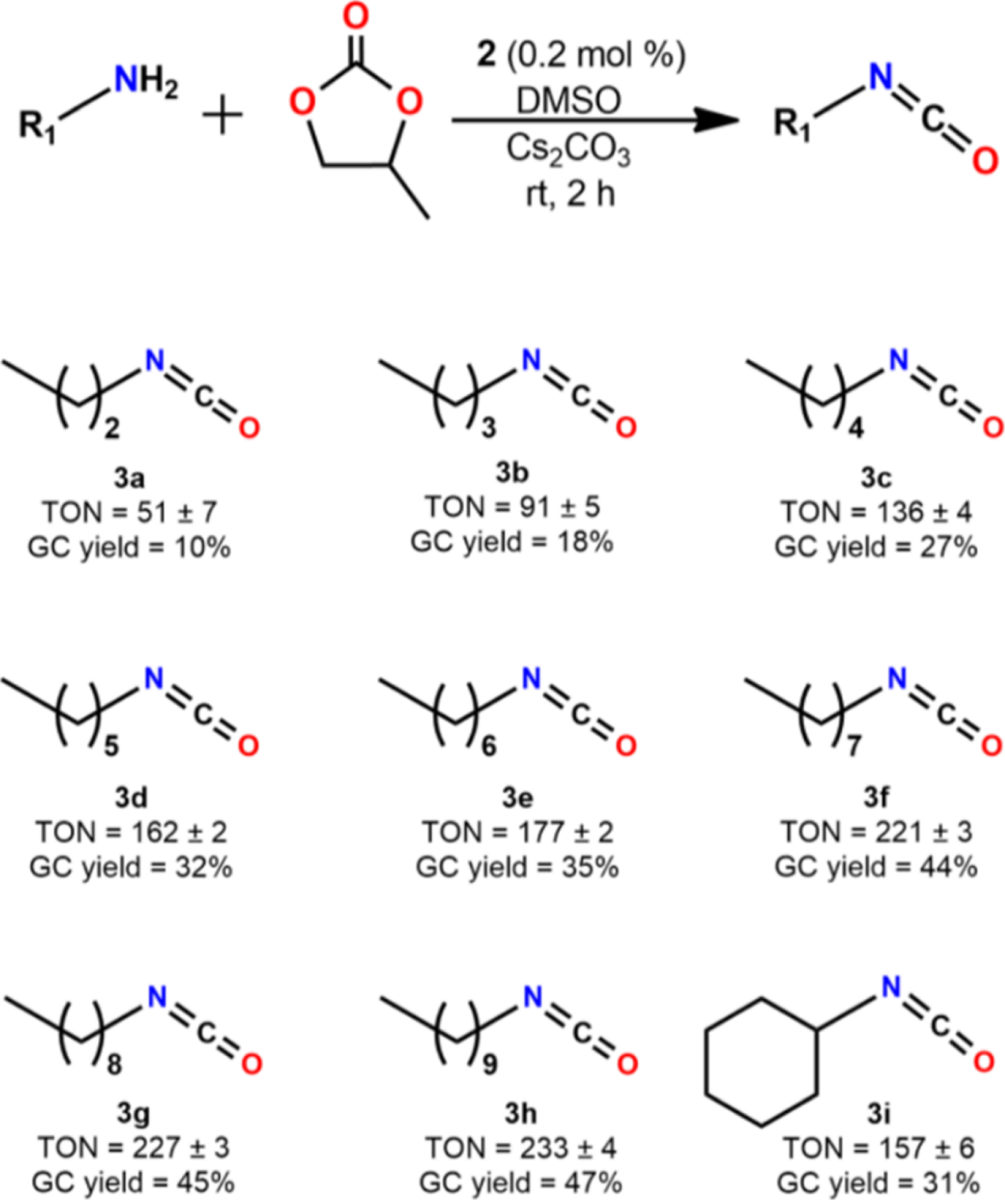
Summary of Isocyanate Products Formed via Oxidative Carbonylation of Aliphatic Amines^a^–^c^

a Conditions: Reactions were carried out with **2** (2 mg, 3.42 × 10^–3^ mmol), Cs_2_CO_3_ (100 eq, 112 mg, 0.342 mmol), propylene carbonate (500 eq, 146 μL, 1.71 mmol), amine (500 eq, 1.71 mmol) in DMSO (1.0 mL).
b Duplicate trials were run.
c The internal standard used for TON analysis was *n*-decane (25.0 μL, 1.28 × 10^–4^ mol).

**Scheme 2:**
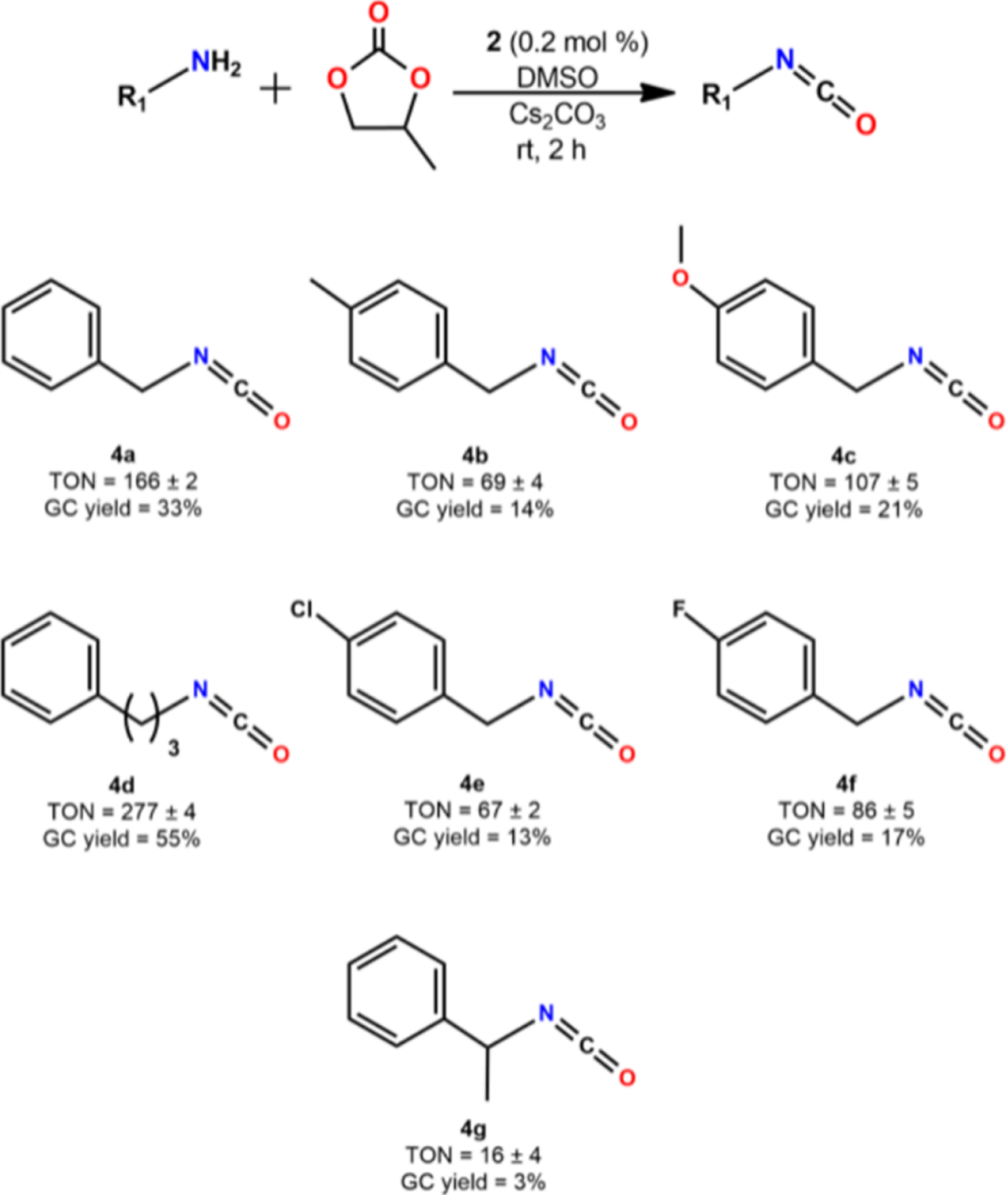
Summary of Isocyanate Products Formed via Oxidative Carbonylation of Aromatically Substituted Amines^a^–^c^

a Conditions: Reactions were carried out with **2** (2 mg, 3.42 × 10^–3^ mmol), Cs_2_CO_3_ (100 eq, 112 mg, 0.342 mmol), propylene carbonate (500 eq, 146 μL, 1.71 mmol), amine (500 eq, 1.71 mmol) in DMSO (1.0 mL).
b Duplicate trials were run.
c The internal standard used for TON analysis was *n*-decane (25.0 μL, 1.28 × 10^–4^ mol).

**Scheme 3:**
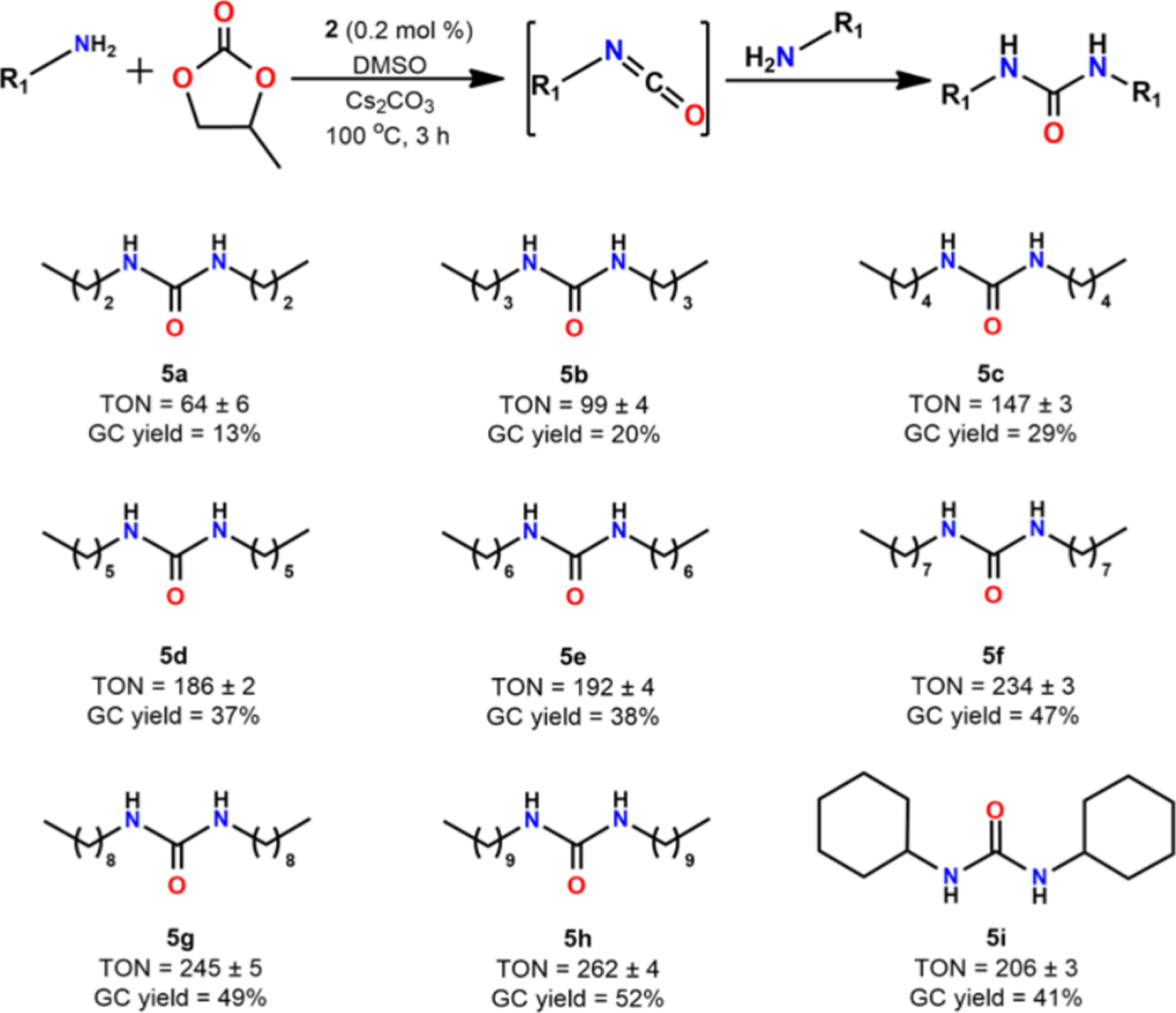
Summary of Urea Products Formed via Propylene Carbonate-Sourced Carbonyls, Oxidatively Added to Various Alkyl-Substituted Amines^a^–^c^

a Conditions: Reactions were carried out with **2** (2 mg, 3.42 × 10^–3^ mmol), Cs_2_CO_3_ (100 eq, 112 mg, 0.342 mmol), propylene carbonate (500 eq, 146 μL, 1.71 mmol), amine (500 eq, 1.71 mmol) in DMSO (1.0 mL).
b Duplicate trials were run.
c The internal standard used for TON analysis was *n*-decane (25.0 μL, 1.28 × 10^–4^ mol).

**Scheme 4:**
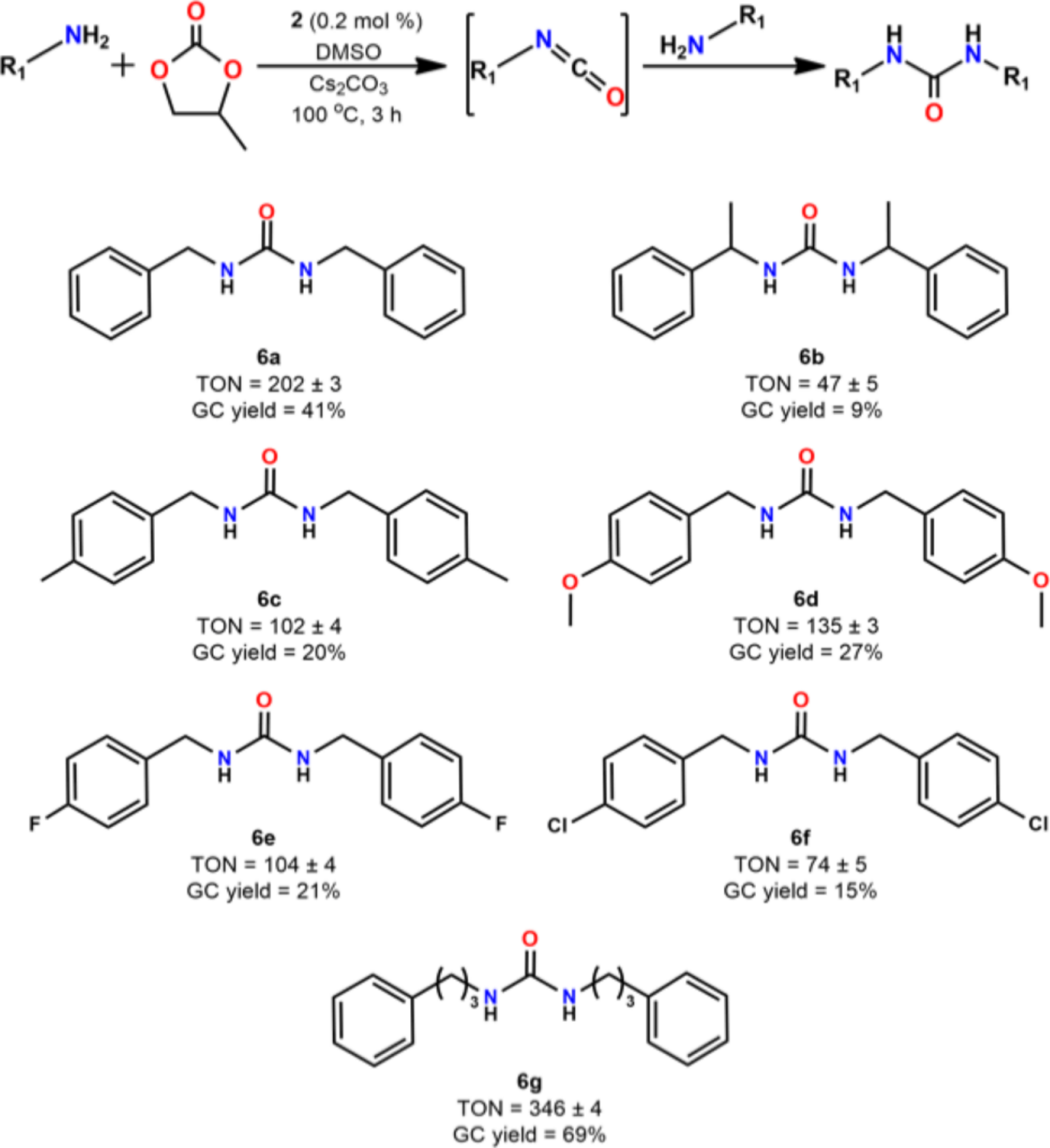
Summary of Urea products Formed via Propylene Carbonate-Sourced Carbonyls, Oxidatively Added to Various Aromatically Substituted Amines^a^–^c^

a Conditions: Reactions were carried out with **2** (2 mg, 3.42 × 10^–3^ mmol), Cs_2_CO_3_ (100 eq, 112 mg, 0.342 mmol), propylene carbonate (500 eq, 146 μL, 1.71 mmol), amine (500 eq, 1.71 mmol) in DMSO (1.0 mL).
b Duplicate trials were run.
c The internal standard used for TON analysis was *n*-decane (25.0 μL, 1.28 × 10^–4^ mol).

## 2 Results and Discussion

### 2.1 Optimization of Base and Solvent

Initial observations of isocyanate production prompted a deeper exploration of this distinctive process for isocyanate formation. Efforts were made to optimize the substrate scope to achieve the desired isocyanate product. A significant part of this study was dedicated to optimizing the choice of base and solvent. Unless stated otherwise, all experiments employed the Nickel­(II) complex (**2**), propylene carbonate (500 eq, 146 μL, 1.71 mmol), and hexylamine (500 eq, 225 μL, 1.71 mmol). Each set of experiments was conducted in duplicate at 25 °C, with stirring for 2 h. The results of these experiments are summarized as mentioned previously in Table 2.

**Table 2:**
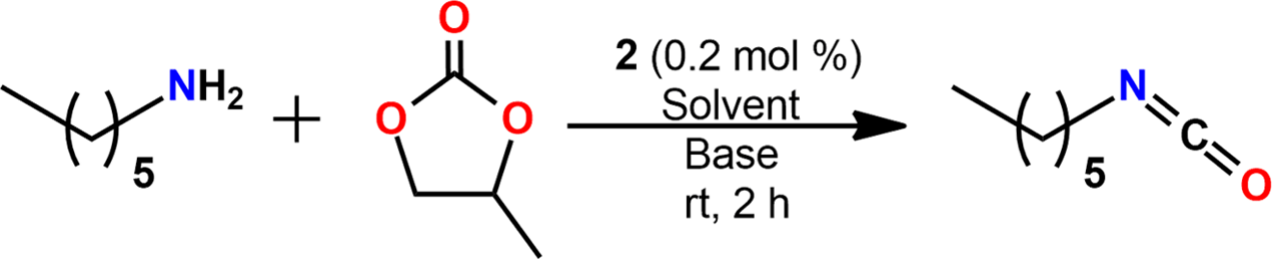
Optimization of Base and Solvent in Terms of Isocyanatohexane Turnover Number (TON) Formation as a Function of Time^a^

entry	base	solvent	TON	yield
1	No base	DMSO	23 ± 2	5%
2	KOH	DMSO	54 ± 3	11%
3	Cs_2_CO_3_	DMSO	162 ± 4	32%
4	LiHMDS	DMSO	134 ± 3	27%
5	K^t^BuO	DMSO	71 ± 4	14%
6	Cs_2_CO_3_	No solvent	53 ± 3	11%
7	Cs_2_CO_3_	DCM	97 ± 4	19%
8	Cs_2_CO_3_	THF	151 ± 4	30%
9	Cs_2_CO_3_	Toluene	88 ± 4	18%
10	Cs_2_CO_3_	Cyclohexane	103 ± 2	21%
11	NiCl_2_	DMSO	not detected	-
12	Cs_2_CO_3_ only	DMSO	not detected	-

a Yields represent an average of duplicate trials.

b TONs were calculated from GC–MS peak integrations using *n*-decane (25.0 μL, 1.28 × 10^–4^ mol) as the internal standard.

c Various bases: KOH = Potassium hydroxide. LiHDMS = Lithium bis­(trimethylsilyl)­amide.

d Various solvents: DMSO = Dimethyl sulfoxide. DCM = Dichloromethane.

e The reaction was run without the catalyst.

First, we conducted base studies using 1.0 mL of dimethyl sulfoxide (DMSO). Each base was tested at 100 mol equiv relative to the Nickel­(II) complex (**2**). Our data indicated that non-nucleophilic bases enhanced the conversion rates of isocyanate products. Among the bases (KOH, KO^t^Bu, LiHMDS, and Cs_2_CO_3_) tested. Among them, cesium carbonate (Cs_2_CO_3_) was found to be the most effective in promoting isocyanate formation. Notably, without a base, the reaction produces the lowest isocyanate yield (5%) (Table 2, Entry 1), indicating the positive role of the base. The solvents DMSO, Dichloromethane, THF, Toluene, and cyclohexanone show varied performance when combined with bases. Cs_2_CO_3_ shows the trend in % Yield across different solvents: No solvent (11%) < Toluene (18%) < Dichloromethane (19%) < Cyclohexane (21%) < THF (30%) < DMSO (32%). On the other hand, DMSO, the best solvent, shows a trend across different bases: KOH (11%) < KO^t^Bu (14%) < LiHMDS (27%) < Cs_2_CO_3_ (32%). The reason for the observed trend is not clear to us. Possibly the characteristic of cesium acting as a base^76,77^ or the solubility of base in the reaction medium play a combined role in contributing to the reaction performance. An experiment was also run with nickel­(II) chloride instead of **2** but no product was detected indicating the importance of pincer ligand (Table 2, entry 11). Experiment was also performed with base only (Entry 12, Table 2) and no product was detected.

DMSO, which was used in other studies, to determine their efficacy in isocyanatohexane synthesis. In each trial, cesium carbonate (Cs_2_CO_3_) (100 eq, 112 mg, 0.342 mmol) was incorporated. When no solvent was specified, the reaction utilized 1.0 mL of propylene carbonate. All other experiments adhered to the conditions mentioned earlier. Our results indicated that DMSO was the optimal solvent for facilitating isocyanatohexane production. This phenomenon can be attributed to the increased nucleophilicity that amine substrates exhibit when introduced into polar aprotic solvents like DMSO.^78^ Remarkably, significant quantities of the isocyanate were produced when propylene carbonate functioned both as the solvent and reagent (in the no solvent setup).

### 2.2 Optimization of Time and Temperature for Isocyanate Product

After identifying the most effective base and solvent in the scope of the isocyanate product, we proceeded to examine the best time and temperature conditions. Each test utilized the Nickel­(II) complex (**2**), propylene carbonate (500 eq, 146 μL, 1.71 mmol), hexylamine (500 eq, 225 μL, 1.71 mmol), and Cs_2_CO_3_ (100 eq, 112 mg, 0.342 mmol). These evaluations were conducted in duplicate and spanned time intervals up to 3 h, at three temperatures: room temperature (rt, 25 °C), 50 °C, and 100 °C. The most effective temperature for isocyanate formation was determined to occur at 25 °C. We found that 25 °C was the optimal temperature for isocyanate formation and that 120 min was the optimal duration. These results are summarized in Figure 3a. Remarkably, at elevated temperatures, we observed reduced TONs for the isocyanate product. The GC–MS analysis further highlighted the significant formation of urea products. Consequently, subsequent studies were oriented to assess both isocyanate and urea product yields over time.

**Figure 3:**
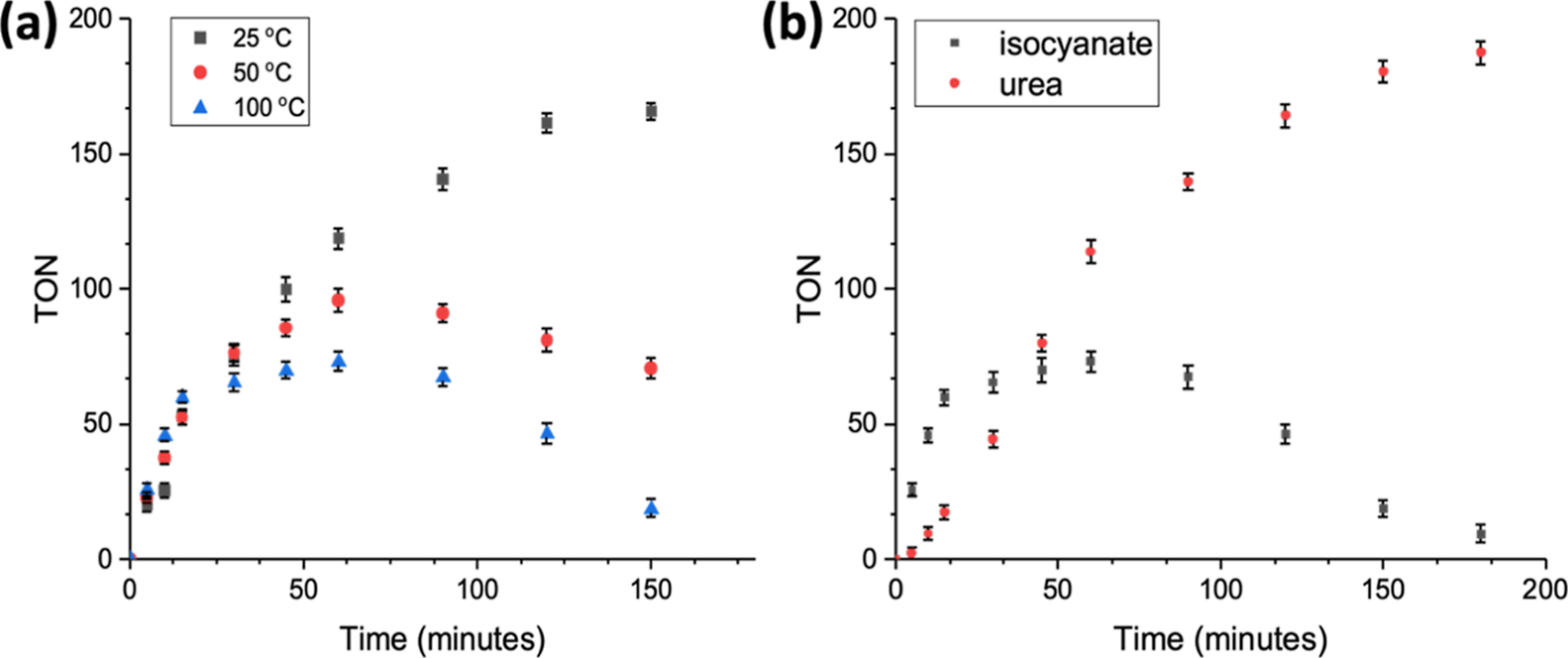
(**a**) Effects of temperature in terms of isocyanatohexane turnover number (TON) formation as a function of time over 150 min. (**b**) Effects of isocyanatohexane versus 1,3-dihexylurea turnover number (TON) formations at 100 °C as a function of time over 180 min. Conditions: Reactions for temperature studies were carried out with **2** (2 mg, 3.42 × 10^–3^ mmol), Cs_2_CO_3_ (100 eq, 112 mg, 0.342 mmol), propylene carbonate (500 eq, 146 μL, 1.71 mmol), hexylamine (500 eq, 225 μL, 1.71 mmol) in DMSO (1.0 mL). Duplicate trials were run. The internal standard used for TON analysis was *n*-decane (25.0 μL, 1.28 × 10^–4^ mol).

After establishing the optimal base, solvent, and temperature for the synthesis of the isocyanate product, we turned to identifying the optimal reaction time, particularly for urea product formation. Each trial, conducted in duplicate, employed the Nickel­(II) complex (**2**), propylene carbonate (500 eq, 146 μL, 1.71 mmol), hexylamine (500 eq, 225 μL, 1.71 mmol), and Cs_2_CO_3_ (100 eq, 112 mg, 0.342 mmol) and was carried out at 100 °C. Drawing insights from the previous time study, we assessed urea TONs at different intervals over 3 h. The optimal duration for urea formation was identified as 180 min. The relationship between the diminishing isocyanate product and the increasing urea formation is detailed in the graphical representation provided in Figure 3b. The collected data indicate a potential equilibrium for the isocyanate product; the conversion of amines to isocyanates seems to occur at a rate similar to that of urea production between 15 and 90 min.

### 2.3 Isocyanate Formations with the Reaction of Cyclic Carbonate Alkyl Substituted Amines

After optimizing the reaction conditions, we carried out one-pot nickel-catalyzed oxidative carbonylation of amines using the previously established parameters. We used propylene carbonate as the carbonyl source and reacted it with various aliphatic amine nucleophiles. The resulting alkyl-substituted isocyanate products are detailed in Scheme 1. In total, nine distinct products were synthesized, with yields ranging from fair to good for the target isocyanate products (**3a**, **51**-**5d**, **233**).

From our observations, the length of the carbon chain in the aliphatic, nonbranched amine nucleophile seemed to correlate directly with the yield of the isocyanate product. This correlation is likely due to the increased nucleophilicity of primary amines as the carbon chain lengthens.^79^ As aliphatic chains grow longer, the nucleophilic strength of the amine-coupling partner enhances.^80^ These factors may influence the reaction yield. Consequently, stronger, longer-chain amine nucleophiles showed higher TON conversions for isocyanate products compared to their less nucleophilic counterparts. This could also account for the observed decrease in isocyanate conversions for sterically hindered, branched aliphatic amines. Secondary amines are less nucleophilic because of the strengthened N–H bond in substituted amines.^81^ However, a decent conversion was obtained for the isocyanato Cyclohexane (**3i**, **157**). The highest TON was recorded for the isocyanatodecane product (**3h, 233**), while the lowest yield was recorded for the isocyanatopropane product (**3a**, **51**).

### 2.4 Isocyanate Formations with the Reaction of Cyclic Carbonate and Aromatically Substituted Amines

After exploring the formation of aliphatic isocyanates, we turned our attention to the synthesis of aromatically substituted isocyanates. Using the established one-pot nickel-catalyzed oxidative carbonylation of amines, propylene carbonate served as the carbonyl source. We reacted this with a range of aromatically substituted amine nucleophiles. The resulting aromatically substituted isocyanate products are detailed in Scheme 2. Overall, TONs for the coupled products ranged from 16 (**4g**) to 277 (**4d**).

In general, aromatic substituted isocyanate products exhibited lower TONs compared to their alkyl-substituted counterparts. The reduced reactivity of 1-methyl-4-(isocyantomethyl) benzene (**4b**, **69**) and 1-methoxy-4-(isocyantomethyl) benzene (**4c**, **107**) when compared to their nonsubstituted counterpart, (isocyanatomethyl)­benzene (**4a**, **166**), can be attributed to steric effects. Moreover, electron-withdrawing substituents on the benzene ring seemed to depress the amine’s reactivity. For halogenated substituents, the sigma electron-withdrawing properties of chlorine and fluorine reduced the reactivity of the benzylamine substrates. Particularly with fluorine, its short bond length and strong polarization of the fluorine–carbon bond likely reduced the electron density around the ipso carbon, thereby lessening the amine’s reactivity.^82^ The 1-fluoro-4-(isocyanatomethyl)­benzene product (**4f**, **86**) exhibited a higher TON than the 1-chloro-4-(isocyanatomethyl)­benzene (**4e**, **67**), which can be attributed to properties of fluorine compared to chlorine. The lowest TON was noted for (1-isocyanatoethyl) benzene (**4g**, **16**), possibly due to the reduced nucleophilicity of the sterically hindered, branched amine. Conversely, the 3-phenylpropylamine’s increased nucleophilicity, due to its aliphatic chain, resulted in the highest TON for (3-isocyanatopropyl)­benzene (**4d**, **277**).

### 2.5 Symmetric Urea Formations with the Reaction of Propylene Carbonate and Alkyl-Substituted Amines

After completing the investigations on various isocyanate formations, we turned our attention to the synthesis of symmetric alkyl-substituted ureas. In the one-pot nickel-catalyzed oxidative carbonylation of amines described earlier, propylene carbonate served as the carbonyl source. This substrate was then reacted with a range of aliphatic amine nucleophiles. Initially, the associated isocyanate intermediate was formed, which was then further converted to yield symmetric urea products. These alkyl-substituted urea products are detailed in Scheme 3.

In general, the yields for the urea products were fair to good, ranging from **5a** (**64**) to **5h** (**262**). The TONs for these alkyl-substituted urea products were slightly higher than those for the associated isocyanate products, as seen in Scheme 1. A clear trend emerged: the length of the carbon chain of the aliphatic amine nucleophile was directly proportional to the quantity of urea product formed. Longer aliphatic chains enhanced the amine nucleophilicity through pronounced inductive effects. As a result, urea products synthesized from stronger amine nucleophiles had higher TON conversions compared to those from their less nucleophilic counterparts. No conversions were noted for sterically hindered, branched aliphatic amines. However, a commendable TON conversion was achieved for 1,3-dicyclohexylurea (**5i**, **206**). As expected, the highest TON recorded was for 1,3-didecylurea (**5h**, **262**), while the lowest was for 1,3-dipropylurea (**5a**, **64**).

### 2.6 Symmetric Urea Formations with Further Reaction of Aromatically Substituted Amines

After exploring aliphatic urea formations, we shifted our focus to the synthesis of aromatically substituted urea. The one-pot nickel-catalyzed oxidative carbonylations of amines were performed under the conditions previously described. In this series of experiments, propylene carbonate was employed as the carbonyl source, and the substrate was reacted with various aromatic amine nucleophiles. This sequence produced the associated isocyanate intermediate, which was then further processed to yield symmetric urea products. The results for the aromatically substituted urea products are detailed in Scheme 4.

In general, the yields for these urea products were fair to good, ranging from **6b** (**47**) to **6g** (**346**). The TONs for the aromatically substituted urea products were typically lower than those observed for alkyl-substituted urea. However, compared with the TONs of their corresponding isocyanate products (as shown in Scheme 2), the aromatically substituted urea products showed slightly higher TONs. It is noteworthy that a modest yet significant conversion was observed for the branched product, 1,3-*bis*(1-phenylethyl) urea (**6b**, **47**). A significant TON conversion was also noted for 1,3-dibenzylurea (**6a**, **202**). The highest TON was for 1,3-*bis*(3-phenylpropyl) urea (**6g**, **346**), while the lowest was for 1,3-*bis*(1-phenylethyl) urea (**6b**, **47**).

### 2.7 Kinetics

The formation rates of isocyanatohexane in relation to the measured quantities of propylene carbonate, hexylamine, catalyst (**2**), and Cs_2_CO_3_ were individually examined. Each investigation involved adjusting the quantity of one starting material while keeping the amounts of the other three reagents consistent with the optimized reaction conditions. The reaction duration was consistently set at 2 h. The reaction temperature was maintained at 25 °C. The presented data reflect the TON values across the entire 120 min reaction period.

Initially, TON values were determined by adjusting the propylene carbonate concentration to 1.66–3.39 M. Subsequently, the hexylamine concentration was varied over the same range to obtain its TON values. In the third set of experiments, the concentration of the Nickel­(II) complex (**2**) was adjusted between 1.22 and 3.67 mmol to determine its TON values. Finally, by altering the concentration of the base cesium carbonate (Cs_2_CO_3_) between 0.244 and 0.489 mol, respective TON values were gathered. Graphical representations of these findings, plotting the logarithm of the rate against the logarithm of the concentration, are displayed in Figure 4.

**Figure 4:**
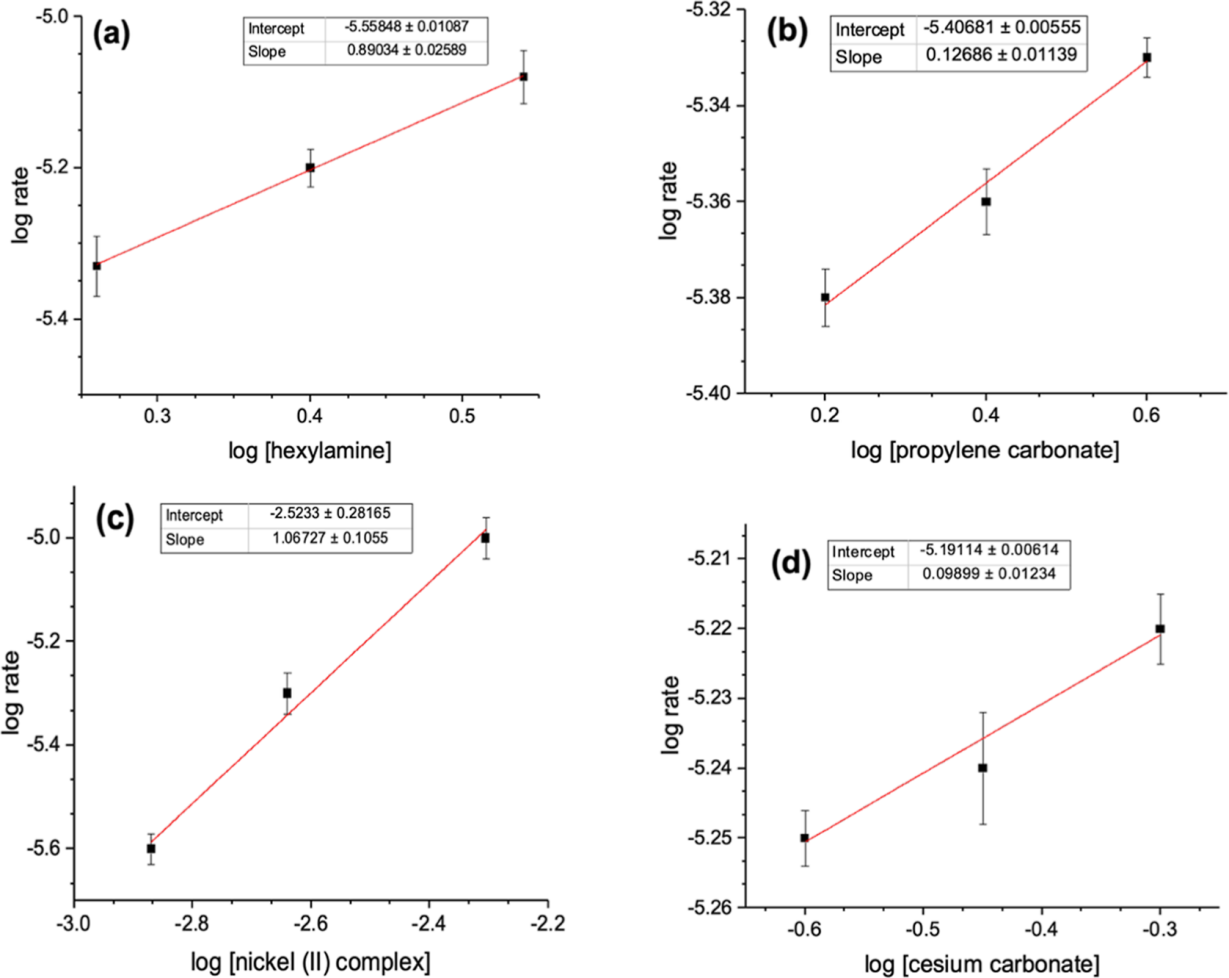
Plots of log rate of isocyanatohexane formation as a function of maintaining concentrations of other starting materials. (**a**) Log rate versus varying log [hexylamine], maintaining all other concentrations constant. (**b**) Log rate versus varying log [propylene carbonate], maintaining all other concentrations constant. (**c**) Log rate versus varying log [Nickel­(II) complex], maintaining all other concentrations constant. (**d**) Log rate versus varying log [cesium carbonate], maintaining all other concentrations constant. Conditions when maintaining concentrations: **2** (2 mg, 3.42 × 10^–3^ mmol), Cs_2_CO_3_ (100 eq, 112 mg, 0.342 mmol), propylene carbonate (500 eq, 146 μL, 1.71 mmol), hexylamine (500 eq, 225 μL, 1.71 mmol) in DMSO (1.0 mL). Duplicate trials were run. The internal standard used for yield analysis was *n*-decane (25.0 μL, 1.28 × 10^–4^ mol). The *R* ^2^ values are (a) = 0.9873, (b) 0.9868, (c) 0.9698 and (d) 0.9774.

By applying differential rate laws, the order of each starting material can be determined from the slope of its respective plot. For the base (Cs_2_CO_3_) and the carbonyl source (propylene carbonate), the reaction order appears to be zero, as indicated by a slope nearly equal to zero. In contrast, for the amine (hexylamine) and the catalyst **(2)**, the reaction demonstrates first-order behavior, as reflected by a slope close to one. This indicates that the system is of second order overall. These findings align with literature values previously reported for other nickel-catalyzed reactions.^83^

### 2.8 Plausible Reaction Pathway

A mechanistic pathway is proposed for the formation of isocyanate and urea catalyzed by the Nickel­(II) pincer complex **(2)**. The nature of nickel, with a wide range of oxidation states from Nickel(0) to Nickel­(IV), plays an important role in the catalytic cycle.^84^ The formation of a divalent Nickel­(II) complex **(2)** potentially occurs in the presence of two deprotonated σ-donating amides of the ligand **(1)**.^85–90^ Yet, given that catalyst **(2)** was synthesized using *n*-butyllithium, the catalyst **(2)** is expected to better mediate reactions in the presence of Li^+^. Due to its small size, Li^+^ can coordinate to donor oxygen atoms. It is plausible that catalyst **(2)** contains imine-type bonding within its structure, suggesting that the amide bonds in **(2)** could be in imine form. Such structures have been previously described in the literature.^91^ Past studies have provided NMR evidence for such structures, possibly arising from ligand asymmetry.^92^

UV–vis spectroscopy was utilized to examine the interactions between complex **(2)** and various substrates and starting materials in DMSO. Figure 5 depicts the changes in absorbance when the Nickel­(II) complex **(2)** is combined with different starting materials. Alone, the catalyst **(2)** exhibits an absorption onset at 550 nm, a shoulder at 500 nm, and peak absorption at 450 nm. Upon mixing with propylene carbonate, the absorbance curve closely mirrors the UV–vis spectrum of the catalyst **(2)** on its own, suggesting no immediate interaction between **(2)** and propylene carbonate. Nonetheless, the combined absorbance curve with propylene carbonate and **(2)** shifts to a shorter wavelength, peaking at 440 nm. This shift is likely due to the solution’s dilution by propylene carbonate. When the amine is introduced to the nickel complex **(2)**, the absorbance peak shifts to a shorter wavelength, and the shoulder disappears, indicating a robust interaction between the amine and the catalyst **(2)**. Incorporating the base Cs_2_CO_3_ into catalyst **(2)** results in a modest alteration of the UV–vis spectrum, leaning toward the IR region. Absorption begins at 550 nm and peaks at 500 nm. A noteworthy observation is the modification of the UV–vis spectrum upon addition of the base, a pivotal step in the catalytic process. It is postulated that the sterically hindered base converts the Nickel­(II) in catalyst **(2)** to a low-valent Nickel­(I).^93^ Additionally, no absorption in the visible spectrum is detected for either hexylamine or propylene carbonate.

**Figure 5:**
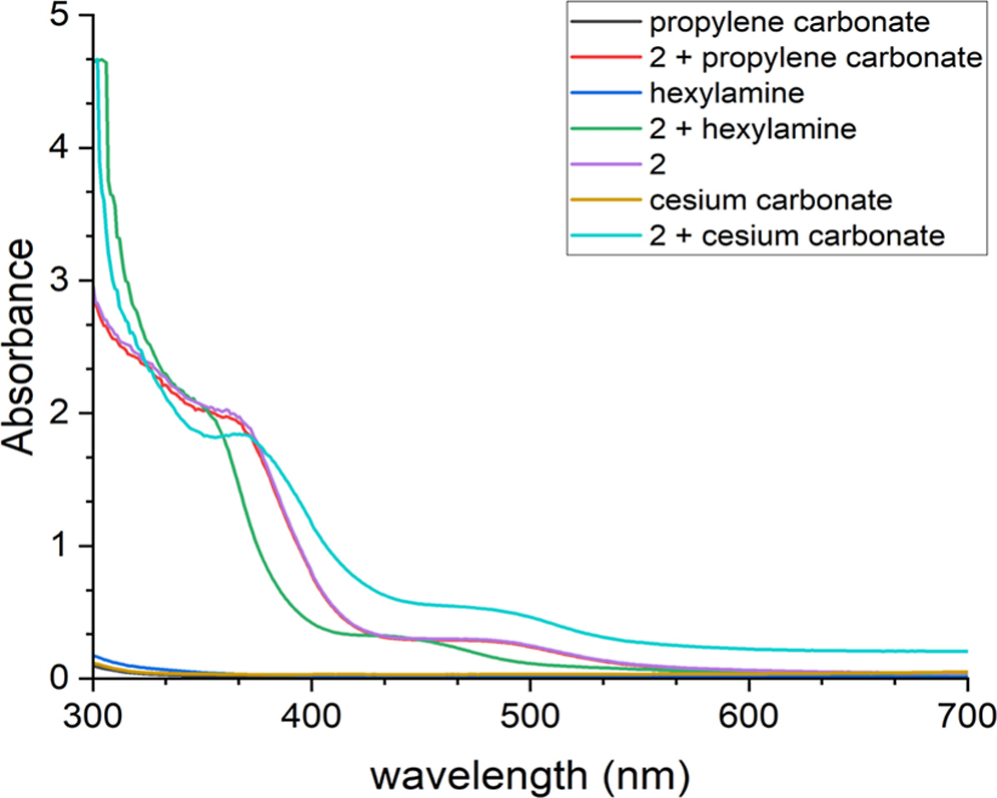
UV–vis studies for **(2)** and various starting materials for isocyanate formation. Conditions for reagents: **2** (2 mg, 3.42 × 10^–3^ mmol), Cs_2_CO_3_ (100 eq, 112 mg, 0.342 mmol), propylene carbonate (500 eq, 146 μL, 1.71 mmol), hexylamine (500 eq, 225 μL, 1.71 mmol) in DMSO (1.0 mL).

Based on the available data, the probable reaction pathway in Figure 6. The mechanism proposed to begin with the coordination of the amine to the metal center, displacing the chloride ligand. **L** stands for the doubly deprotonated ligand, **1**. The base, such as Cs_2_CO_3_, serves primarily to quench the proton generated during the reaction and does not influence the reaction rate or directly participate in the catalytic cycle. The amine plays a dual role, acting both as a substrate and as a potential reductant by priming intermediate (2) for reactivity via a single-electron transfer (SET) process. SET pathways have been proposed in certain nickel-catalyzed transformations,^94,95^ and we propose a similar process operates in this system. Subsequent oxidative addition of propylene carbonate to a Ni­(I) species, accompanied by ring opening, leads to the formation of an acyl alkoxy nickel­(III) intermediate following displacement of the coordinated amine. We have identified this intermediate formation via mass spectrometry. Which is given in the Supporting Information (Figure S41). An experiment was conducted with TEMPO, and no rate change was observed, which is indicative of a nonradical pathway.

**Figure 6:**
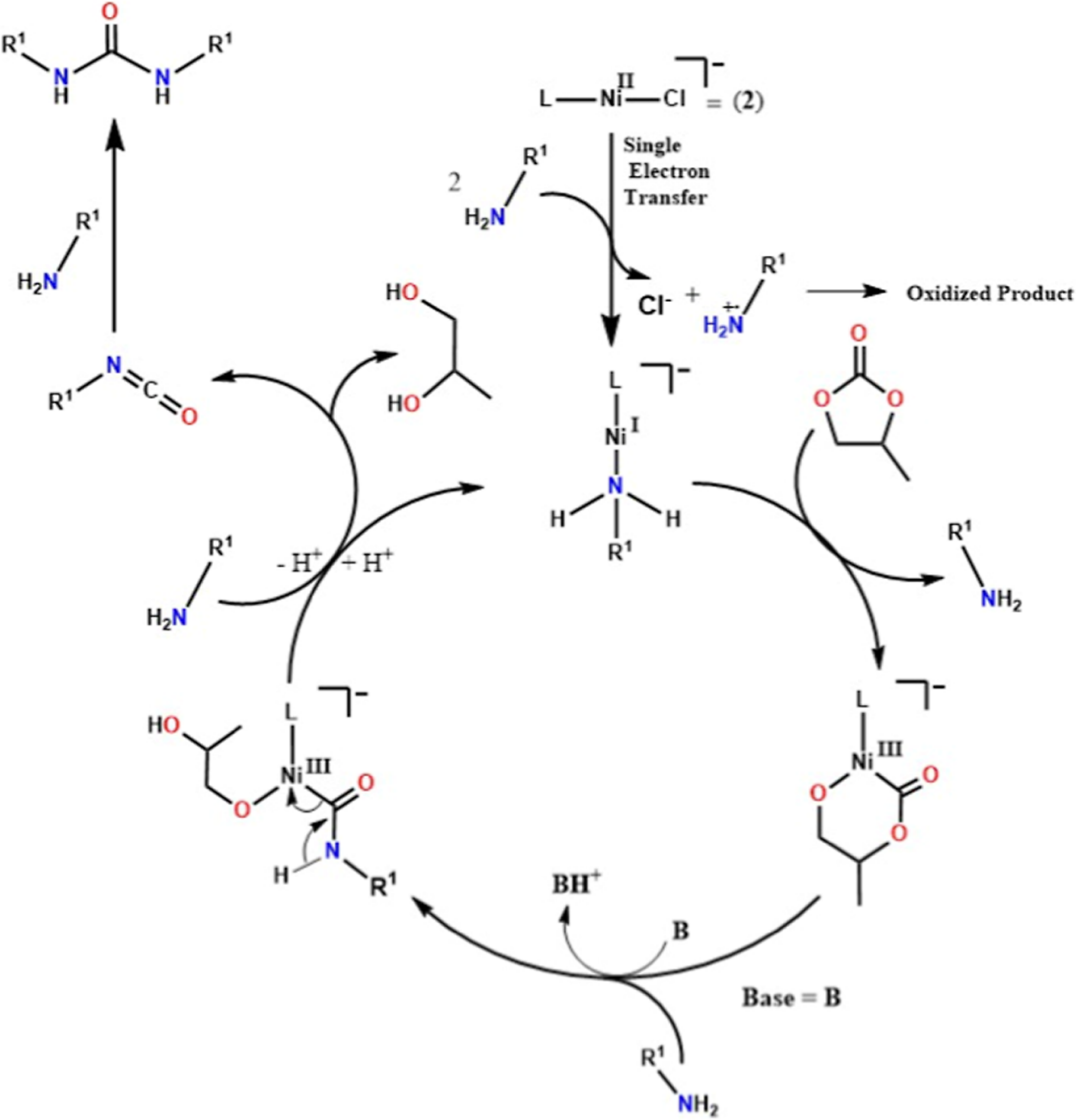
Proposed mechanism in the scope of isocyanates leading to urea formation. **L** stands for a doubly deprotonated ligand, **1**.

Nucleophilic attack of the amine nitrogen on the carbonyl carbon derived from propylene carbonate generates a second acyl nickel­(III) intermediate bearing an alkoxy ligand, with concurrent proton quenching by base. This intermediate then rearranges to form the isocyanate, as shown in Figure 6. The catalytic cycle proceeds with the release of 1,2-propanediol (as identified by GC–MS) and regeneration of the active nickel species, while incorporation of a new propylene carbonate molecule sustains the cycle and affords the desired isocyanate. At elevated or even room temperatures, the isocyanate further reacts with excess amines to yield urea derivatives (Figure 6). The overall catalytic sequence is summarized in Figure 6, and detailed mechanistic analysis of the isocyanate-to-urea transformation is beyond the scope of this study.

## 3 Conclusion

This study demonstrates a new synthesis route for isocyanates and ureas via oxidative carbonylations of amines from carbonyls sourced from cyclic carbonate, catalyzed via a Nickel­(II) pincer catalyst (**2**). This distinctive pincer ligand-based Nickel­(II) complex (**2**) significantly improves catalytic efficiency, enabling the formation of numerous complex products. Without this catalyst, these products do not form, and even with the catalyst alone, product formation is minimal, highlighting the necessity of optimized conditions (base, solvent, temperature, and time). Kinetic studies show a second-order reaction behavior, with zero order for base and propylene carbonate, and first order for the Nickel­(II) complex and amine. The research also proposes a mechanistic cycle, giving insight into the catalytic pathway. Comprehensive GC–MS analysis confirms significant yields of isocyanate and urea products. The study indicates that the best results are achieved within the first 60 to 180 min of the reaction, with 32 different products identified. Alkyl-substituted amines generally show better reactivity than aromatic ones. The calculated TONs for the isocyanate products ranged from moderate (**4g**, **16**) to good (**4d**, **277**), and for the urea products, from moderate (**6b**, **47**) to good (**6g**, **346**).

## 4 Experimental Section

### 4.1 Instrumentation, Materials, and Methods

The chemicals used were purchased from Alfa Aesar (Haverhill, MA, USA), Fischer Scientific Company (Waltham, MA, USA), Sigma-Aldrich (St. Louis, MO, USA), and VWR International (Radnor, PA, USA). Chemicals used are considered pure in their purchased form, and no further purification was performed. Argon (Ar) and nitrogen (N_2_) were purchased from Air Gas. Solvents (SureSeal) were used as obtained and purged with Ar or N_2_ after each use. Amine reagents (liquid) were stored under inert atmosphere before and after each use. Propylene carbonate was contained inside a SureSeal container and purged with Ar before and after use. ^1^H NMR and ^13^C NMR were recorded using a multinuclear JEOL 400 NMR. Electrospray ionization mass spectra (ESI-MS) were obtained using an Agilent 100 series MSD VL. Gas chromatography/mass spectrometry (GC–MS) was used to analyze the isocyanate product conversions. The GC–MS used was a Shimadzu QP2010 Plus and Shimadzu QP2010 *ultra* electron ionization (EI) detection system. The stationary phase consists of a 30 m DB-5 nonpolar column, and He gas is used as the carrier gas. Other GC–MS parameters to note include injection temperature (250 °C), injection volume (1.0 μL), and run-time (37.5 min). Ultraviolet–visible spectra were obtained using a PerkinElmer Lambda 365 UV–vis. FT-IR spectra were obtained using a Thermo Fisher Scientific Nicolet 6700.

The internal standard method was used to calculate turnover numbers (TON) for the converted isocyanate products according to a previously reported procedure.^74^ The internal standard used was *n*-decane. Areas under the curve integrations of the internal standard peaks and target product peaks were ratioed, and a dilution factor was applied to calculate the number of moles of product formed in the reaction mixture. TON was then calculated from the quotient of total moles of formed products and total moles of catalyst used. TON indicates the number of chemical conversions from the substrate added to the chemical reaction. GC yields are provided for a comparison of product formation.

### 4.2 Synthesis Pincer Ligand (**1**)

According to the literature,^91,96^ ligand synthesis was performed under a nitrogen (N_2_) atmosphere. 2,6-pyridinedicarbonyl dichloride was dissolved in dichloromethane (DCM) and added dropwise to a solution of 2,6-diisopropylaniline and triethylamine (Et_3_N). The reaction mixture was stirred and refluxed at 50 °C for 6 h. The organic product was recovered from DCM and washed with aqueous solutions of 5% sodium bicarbonate (NaHCO_3_), followed by 3% hydrochloric acid (HCl). Sodium sulfate (Na_2_SO_4_) was used to remove any remaining water from the DCM mixture prior to vacuum filtration. The solvent was removed by rotary evaporation, and the precipitated white solid was recovered. The product was recrystallized using hexane. The resulting product was further dried overnight under negative pressure. After collecting the product, a white, flaky solid residue was collected. The mass was recorded at 687 mg, yielding 87%. Characterizations for the ligand product **(1)** were performed via FT-IR, ^1^H NMR, and ^13^C NMR.

### 4.3 Synthesis of Nickel­(II) Complex (**2**)

According to the literature,^91,96,97^ metalation of the ligand was performed under N_2_ atmosphere. **(1)** was dissolved in tetrahydrofuran (THF), and the solution temperature was reduced to 0 °C. *N*-butyllithium (2.1 mol eq) was then slowly added to the solution mixture. Maintaining the temperature, Nickel­(II) dichloride glyme (C_4_H_10_Cl_2_NiO_2_) (1.1 mol eq) was added to the reaction mixture 15 min after the base was added. Low temperature was maintained throughout the reaction, which was stirred overnight. A liquid nitrogen cold trap was used for solvent removal, and the resulting solid was dried under negative vacuum pressure. After collecting the fine wine-red powder product, the mass was recorded as 771 mg, yielding 93%. Characterizations for the Nickel­(II) pincer complex **(2)** were performed via ESI-MS negative mode and FT-IR. It is believed that the chlorometalated form (**2)** may get converted to a hydroxymetalated catalyst in the presence of moisture, which could also exhibit catalytic activity.^97,98^

### 4.4 General Synthesis of Isocyanates and Ureas

C–N cross-coupling reactions were performed in duplicate trials. Mixing amines with propylene carbonate depended on the target product and was performed in a one-pot process. The Ni­(II) complex **(2)** (2 mg, 3.42 × 10^–3^ mmol) was weighed and added to a dry Pyrex test tube, then dissolved in anhydrous DMSO (1.0 mL), and transferred to the test tube with a gastight syringe. All ensuing molar equivalents (eq’s) for remaining starting materials were calculated from the initial molar amount of **(2)**. In sequence, cesium carbonate (Cs_2_CO_3_) (100 eq, 112 mg, 0.342 mmol), followed by the propylene carbonate (500 eq, 146 μL, 1.71 mmol) and corresponding amine (500 eq, 1.71 mmol) starting reagents were added to complete the final reaction mixture. The reaction mixture was stirred at room temperature for 2 h to produce isocyanate products. The reaction mixture was stirred at 100 °C for 3 h.

After the reaction was complete, the mixture was filtered through Celite to remove any solid residues, and then the sample was prepared for postreaction analysis by GC–MS. A portion (100.0 μL) of the filtered reaction mixture was transferred to a GC–MS sample vial. An internal standard of *n*-decane (25.0 μL, 1.28 × 10^–4^ mol) was added, and the sample was diluted with DCM to a final volume of 1.50 mL. Following the GC–MS analyses, quantitative evaluations were conducted based on peak area integrations of the standard *n*-decane and the desired isocyanate products.

## Supplementary Material



## Notes

GC–MS data for all isocyanate and urea products (Figures S1–S32); FT-IR, ^1^H NMR, and ^13^C NMR spectra of the NNN ligand (Figures S33–S35); ESI–MS and FT-IR characterization of the Ni­(II) complex (Figures S36–S38); ^1^H and ^13^NMR of 1,3-diheptyl urea (Figures S39 & S40) and mass spectrum of catalytic intermediate (Figure S41) (PDF)
