# Supplementary material for: Pincer Nickel(II) Catalyzed Oxidative Carbonylation of Amines: A Phosgene-Free Synthesis for Isocyanates and Ureas
Source: ACS Omega. 2026 Jun 8;11(24):35561–74. doi: 10.1021/acsomega.6c01401 (PMC13294947; doi:10.1021/acsomega.6c01401)

Supporting Information

Pincer Nickel(II) Catalyzed Oxidative Carbonylation of Amines: A Phosgene-Free Synthesis for Isocyanates and Ureas

Peter Szwedo, Raja Shekhar Kondrapolu, Pradip Munshi*, and Anindya Ghosh*

Corresponding authors:

Anindya Ghosh

School of Physical Sciences, Chemistry Program, University of Arkansas at Little Rock, 2801 South University Avenue, Little Rock, AR 72204, USA. Email: axghosh@ualr.edu

Pradip Munshi:

CISCHEM, Centre for Innovation and Sustainable Chemistry, Kalpvruksh Complex, Vadodara, Gujarat 390021, India. Email: pradip.munshi@cischem.org

Authors:

Peter Szwedo, Raja Shekhar Kondrapolu:

School of Physical Sciences, Chemistry Program, University of Arkansas at Little Rock, 2801 South University Avenue, Little Rock, AR 72204, USA.

Table of Contents

Gas Chromatography–Mass Spectrometry

Figure S1: 1-isocyanatopropane S5

Figure S2: 1-isocyanatobutane S6

Figure S3: 1-isocyanatopentane S7

Figure S4: 1-isocyanatohexane S8

Figure S5: 1-isocyanatoheptane S9

Figure S6: 1-isocyanatooctane S10

Figure S7: 1-isocyanatononane S11

Figure S8: 1-isocyanatodecane S12

Figure S9: isocyanatocyclohexane S13

Figure S10: (isocyanatomethyl)benzene S14

Figure S11: 1-(isocyanatomethyl)-4-methylbenzene S15

Figure S12: 1-(isocyanatomethyl)-4-methoxybenzene ... S16

Figure S13: (3-isocyanatopropyl)benzene S17

Figure S14: 1-chloro-4-(isocyanatomethyl)benzene S18

Figure S15: 1-fluoro-4-(isocyanatomethyl)benzene S19

Figure S16: (1-isocyanatoethyl)benzene S20

Figure S17: 1,3-dipropylurea S21

Figure S18: 1,3-dibutylurea S22

Figure S19: 1,3-dipentylurea S23

Figure S20: 1,3-dihexylurea S24

Figure S21: 1,3-diheptylurea S25

Figure S22: 1,3-dioctylurea S26

Figure S23: 1,3-dinonylurea S27

Figure S24: 1,3-didecylurea S28

Figure S25: 1,3-dicyclohexylurea S29

Figure S26: 1,3-dibenzylurea S30

Figure S27: 1,3-bis(4-methylbenzyl)urea S31

Figure S28: 1,3-bis(4-methoxybenzyl)urea S32
Figure S29: 1,3-diphenylpropylurea S33
Figure S30: 1,3-bis(4-chlorobenzyl)urea S34
Figure S31: 1,3-bis(4-fluorobenzyl)urea S35
Figure S32: 1,3-bis(1-phenylethyl)urea S36

Other Instrumentation

Figure S33: FT-IR of NNN Ligand S38
Figure S34: ^{1}H NMR of NNN Ligand S39
Figure S35: ^{13}C NMR of NNN Ligand S40
Figure S36: ESI-MS (negative mode) of Ni(II) complex ... S41
Figure S37: FT-IR of Ni(II) complex S42
Figure S38: ^{1}H NMR of Ni(II) complex S43
Figure S39: ^{1}H NMR of 1-3-diheptyl urea S44
Figure S40: ^{13}C NMR of 1-3-diheptyl urea S45
Figure S41: Mass spectrum of the acyl alkoxy nickel(III) intermediate.................S45

Gas Chromatography-Mass Spectrometry

Figure S1: 1-isocyanatopropane

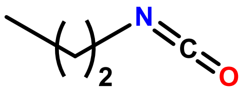

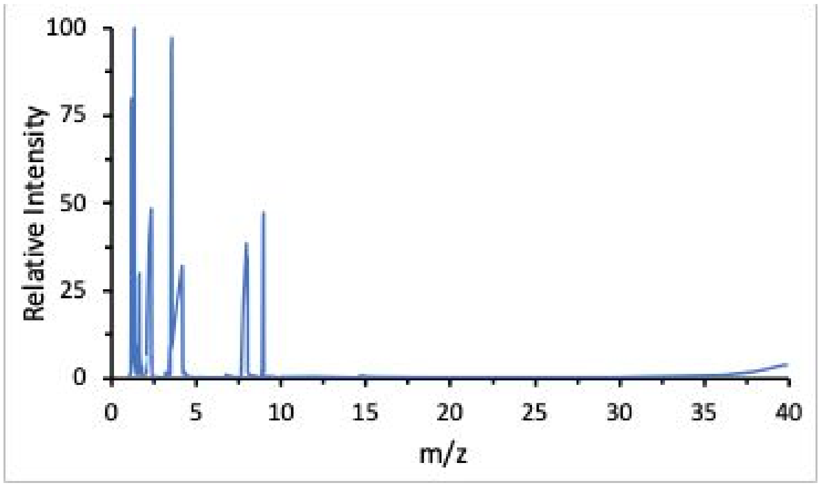

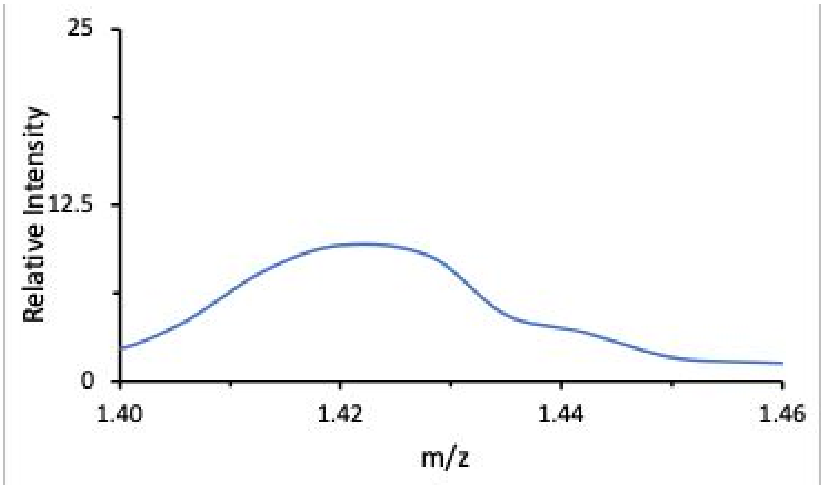

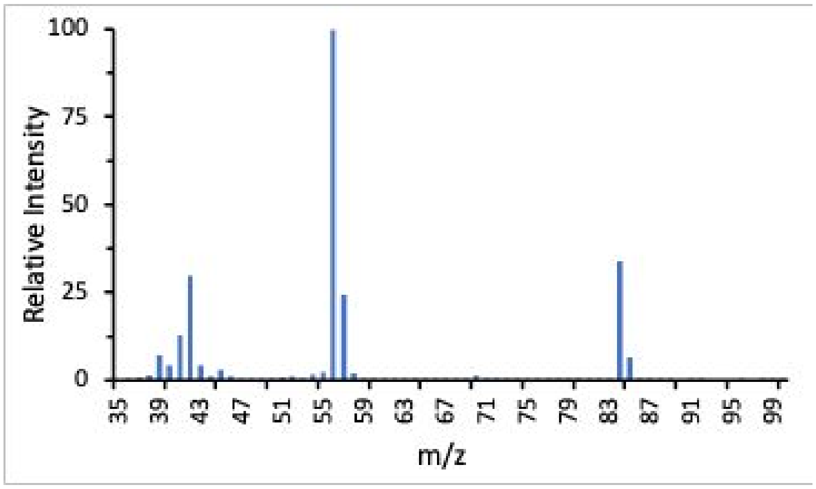

Figure S2: 1-isocyanatobutane

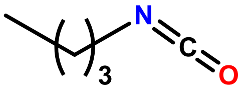

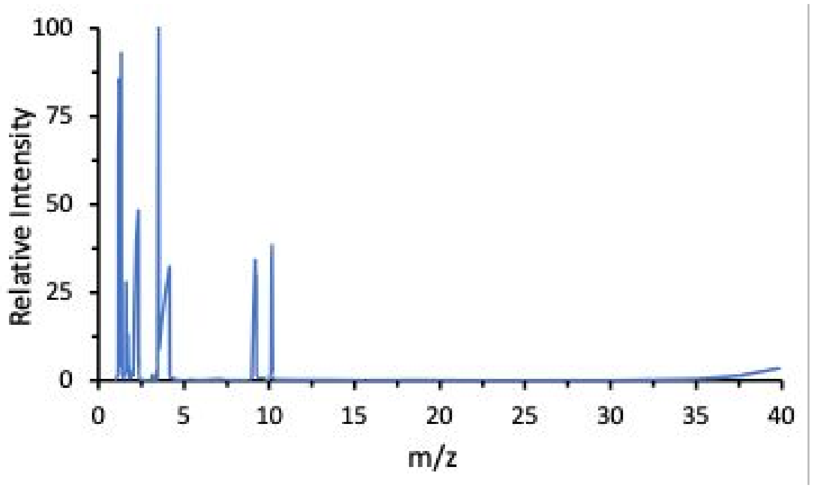

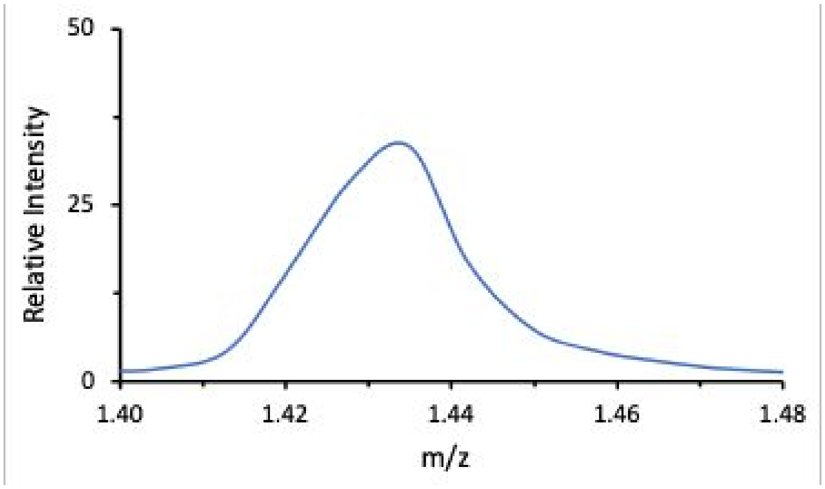

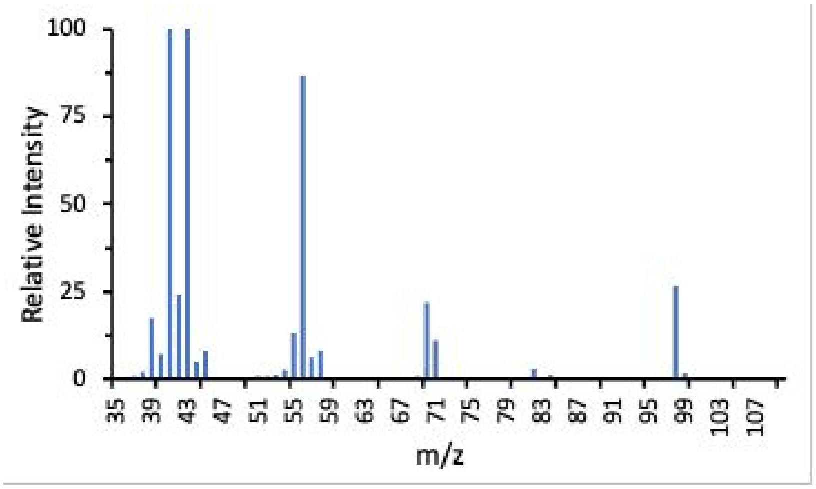

Figure S3: 1-isocyanatopentane

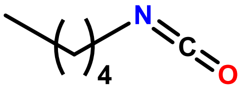

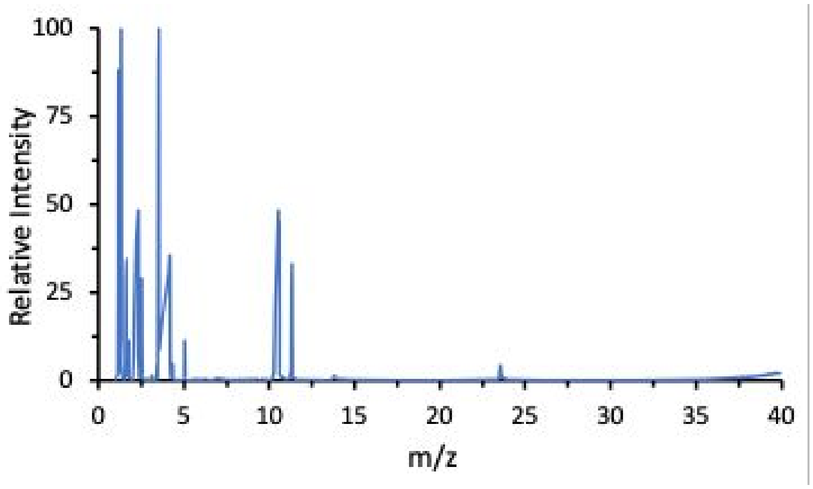

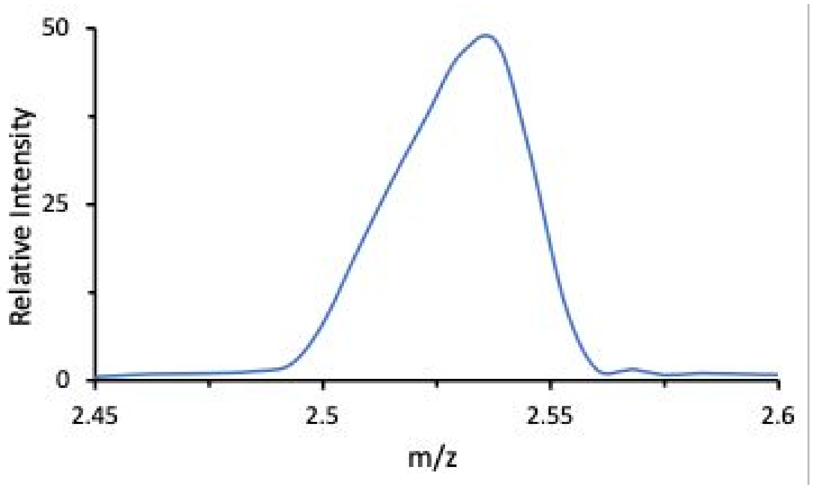

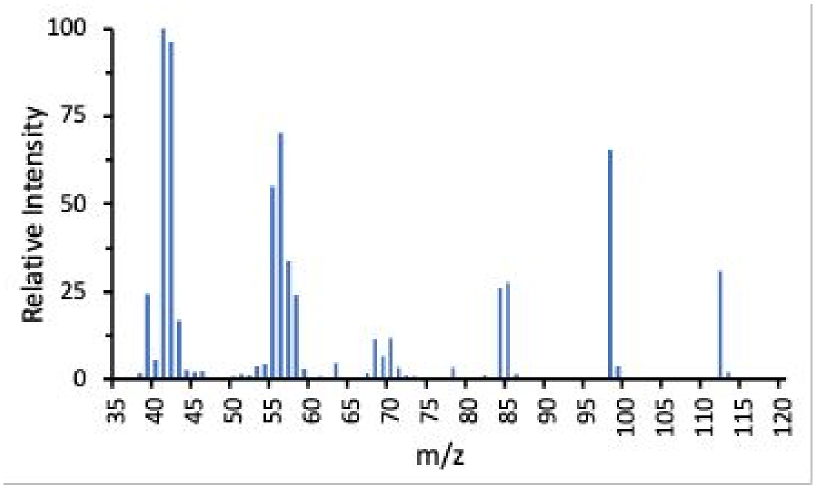

Figure S4: 1-isocyanatohexane

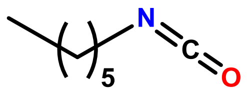

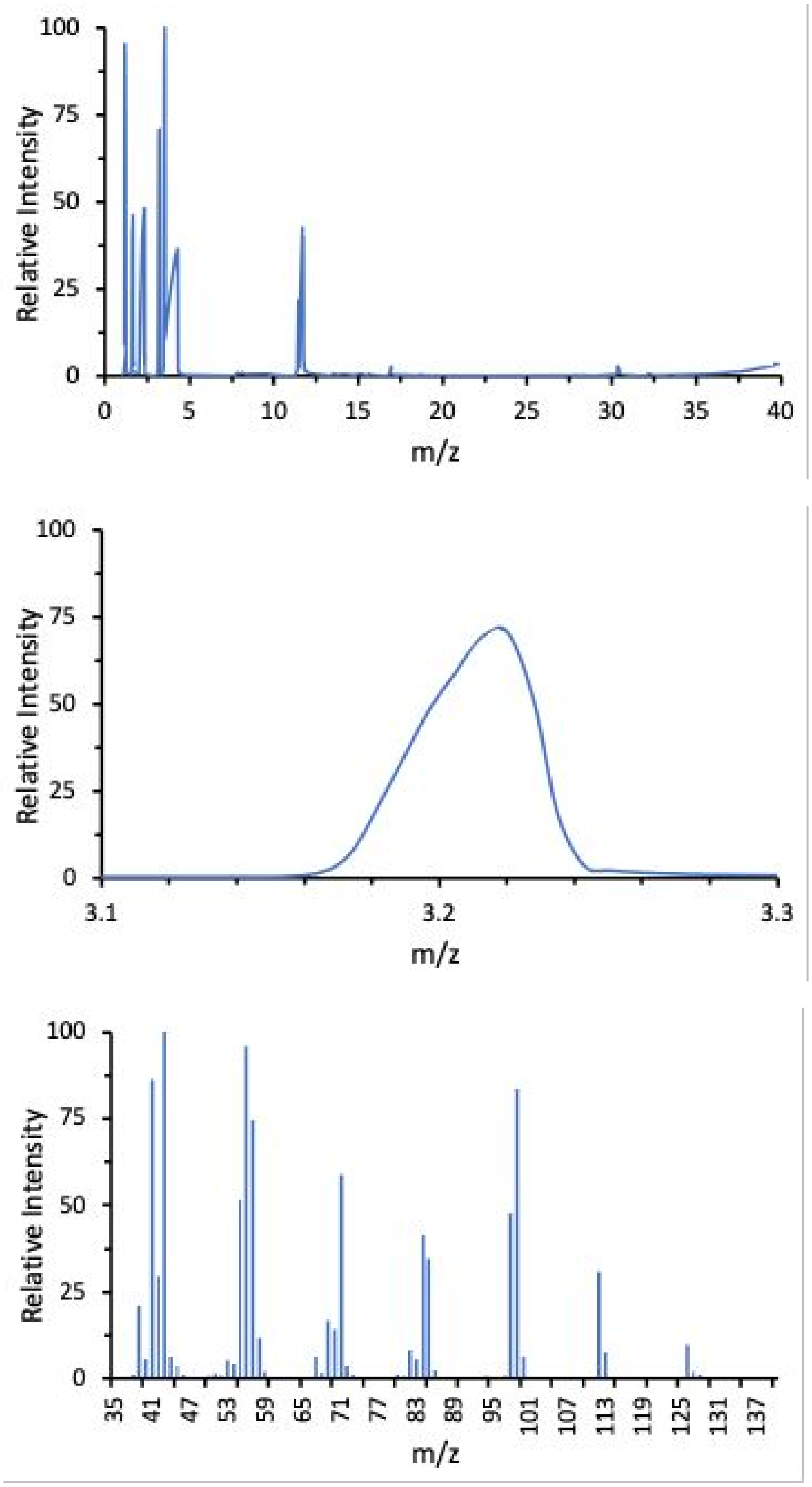

Figure S5: 1-isocyanatoheptane

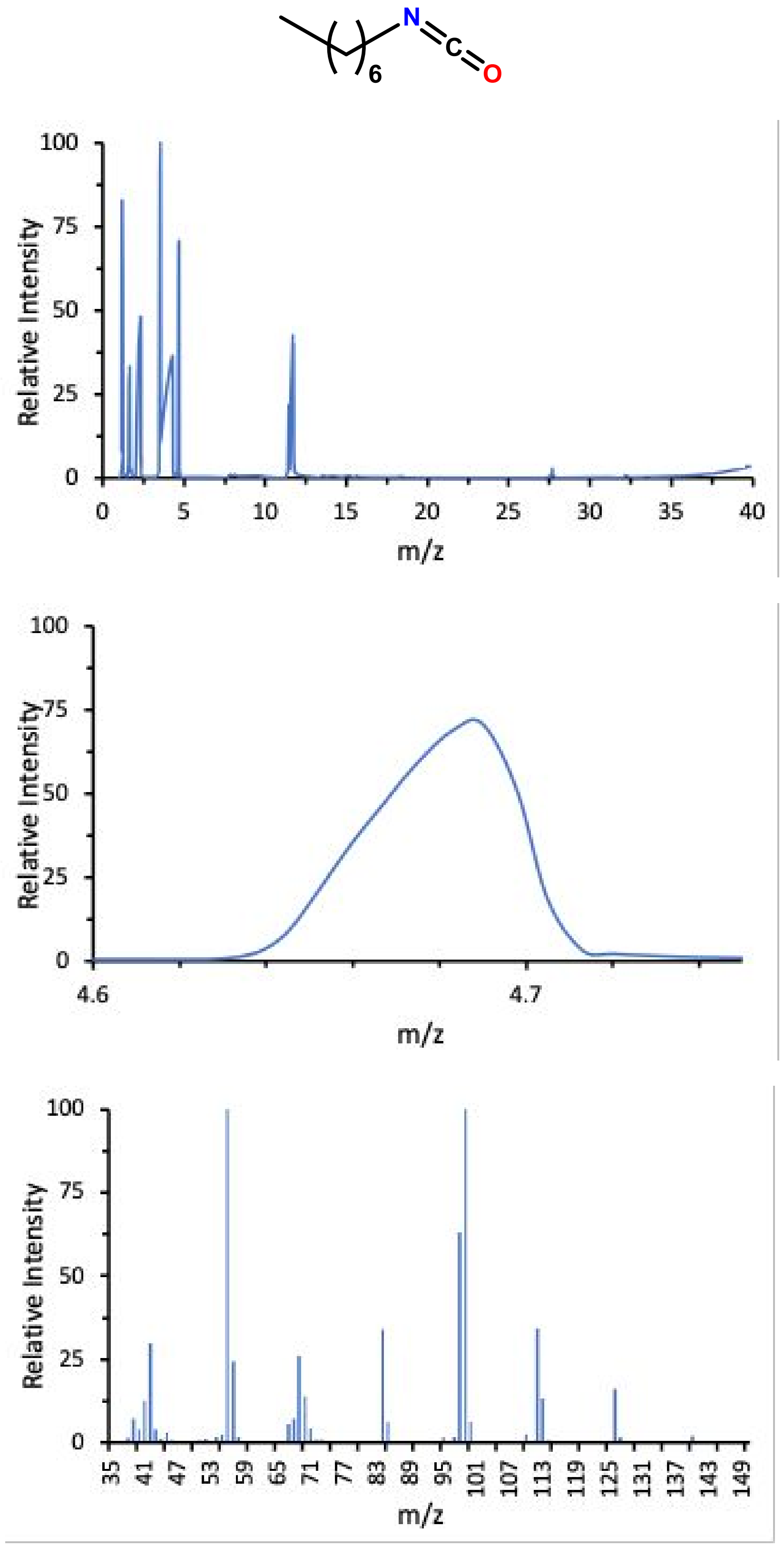

Figure S6: 1-isocyanatoocatane

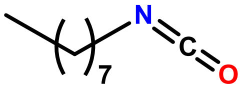

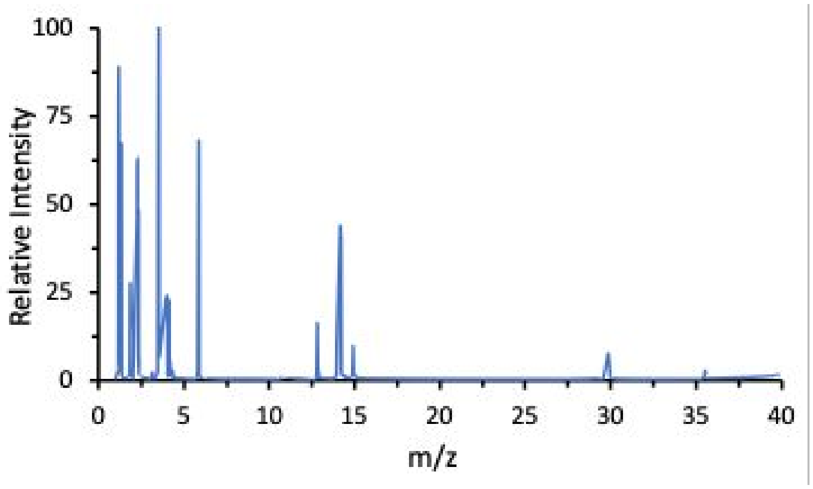

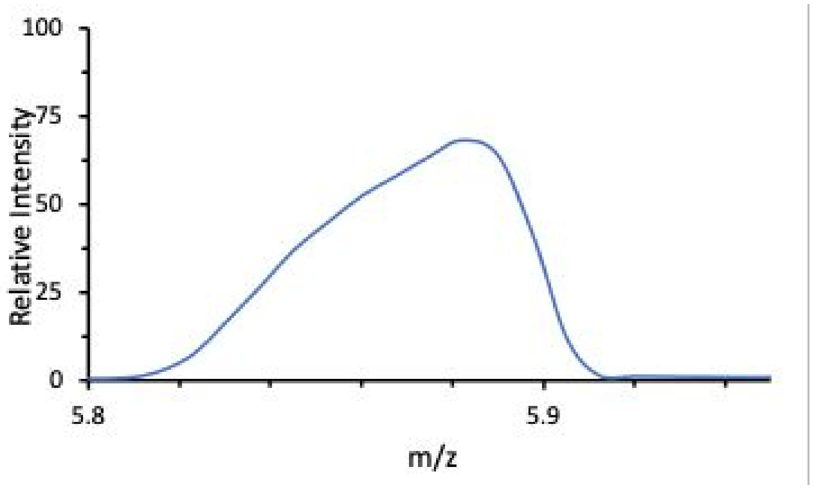

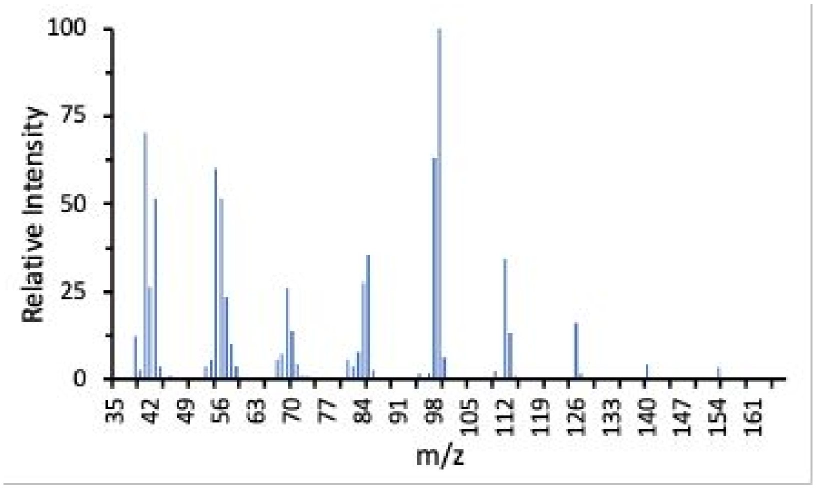

Figure S7: 1-isocyanatononane

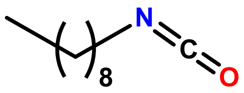

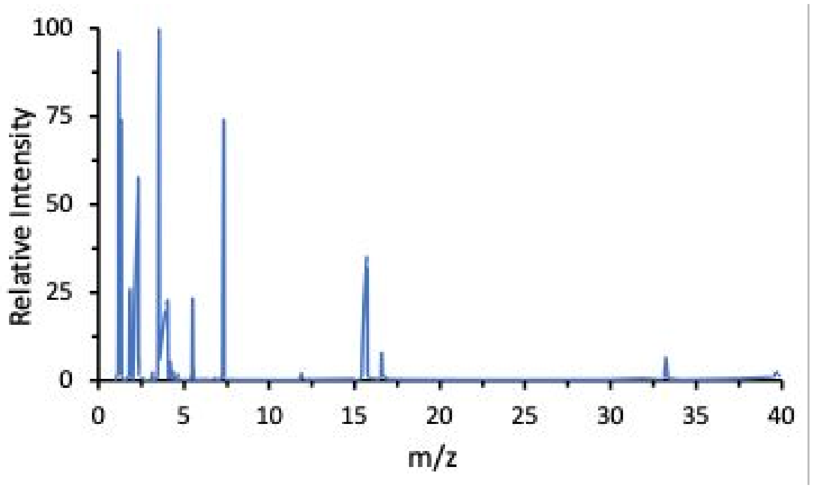

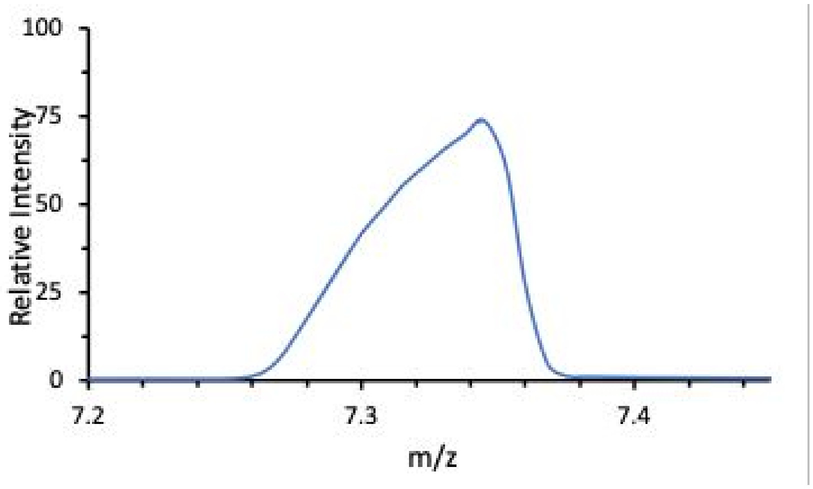

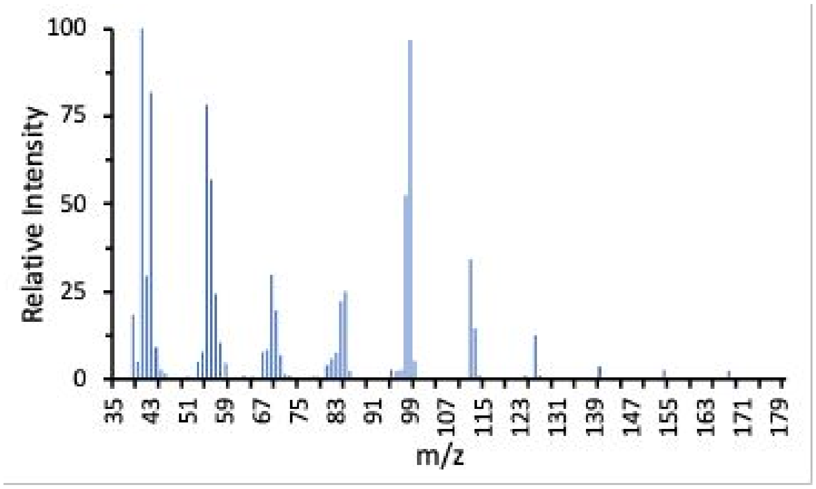

Figure S8: 1-isocyanatodecane

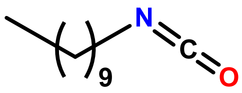

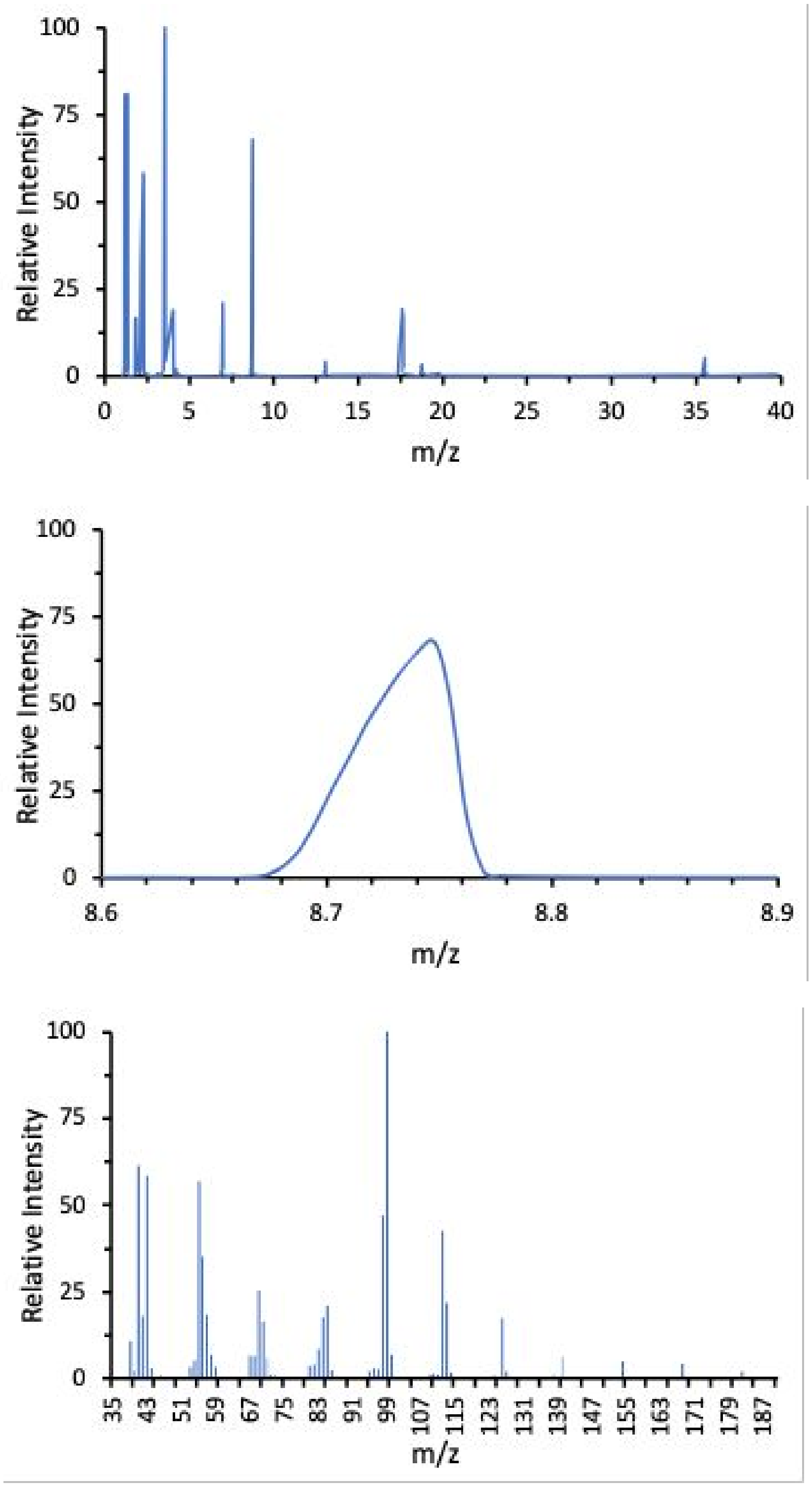

Figure S9: isocyanatocyclohexane

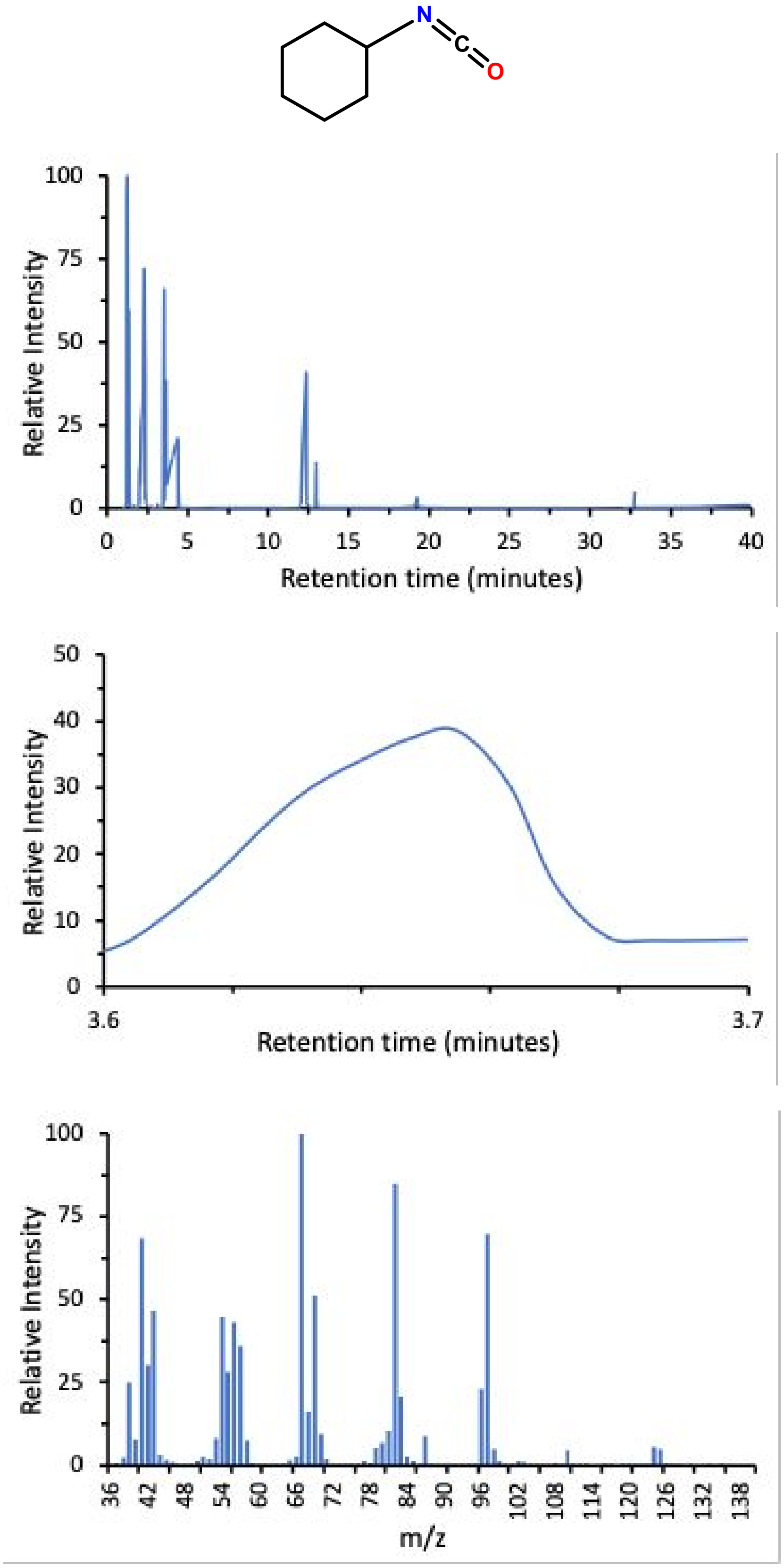

Figure S10: (isocyanatomethyl)benzene

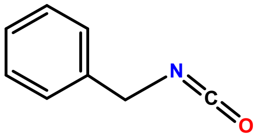

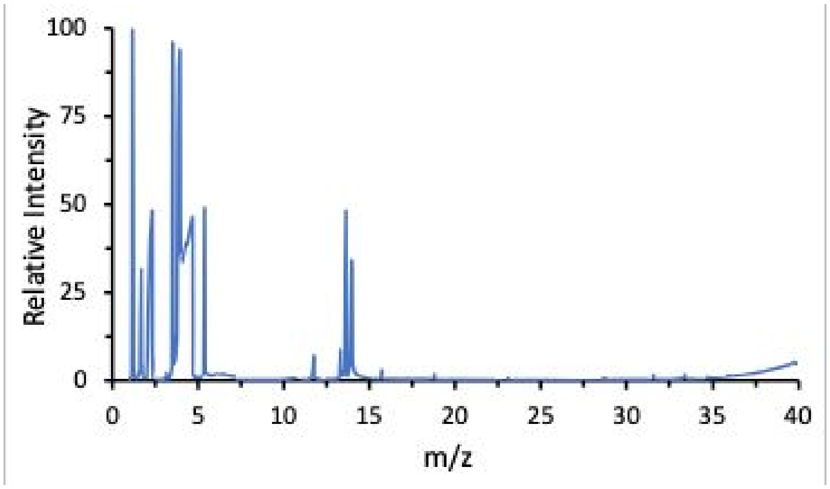

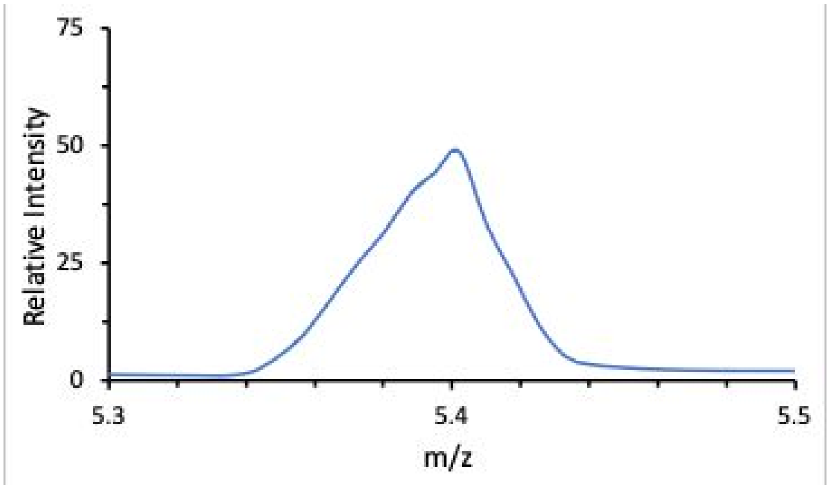

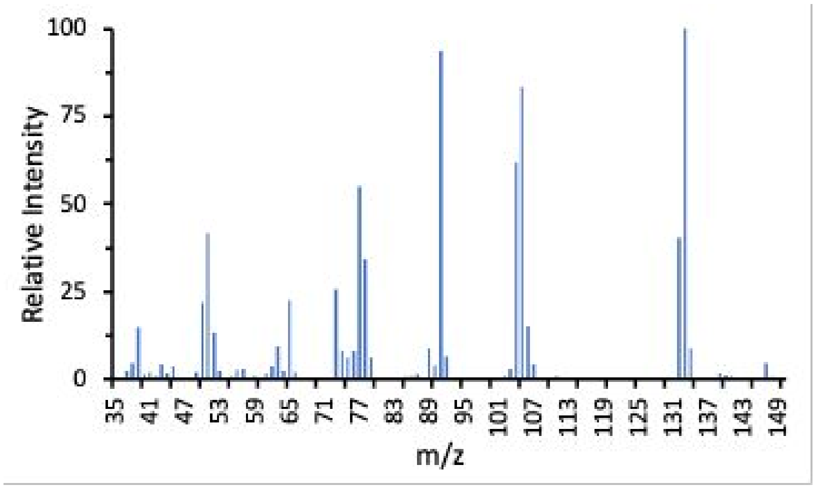

Figure S11: 1-(isocyanatomethyl)-4-methylbenzene

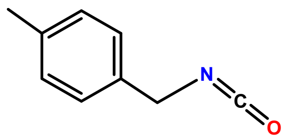

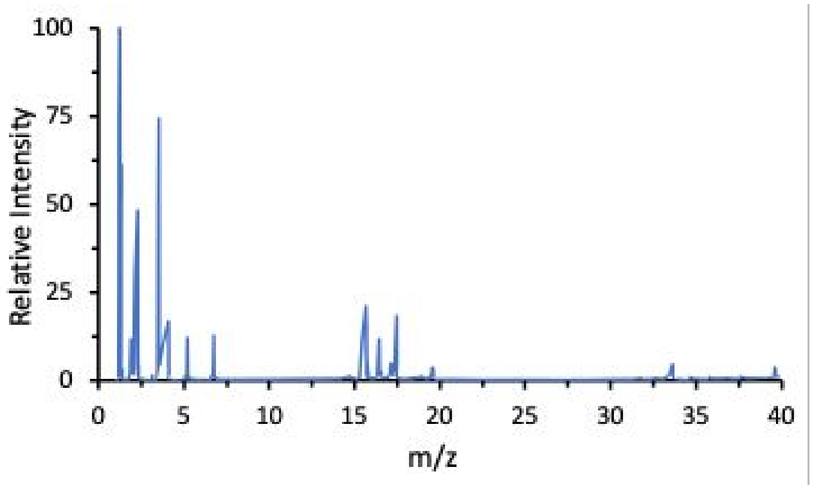

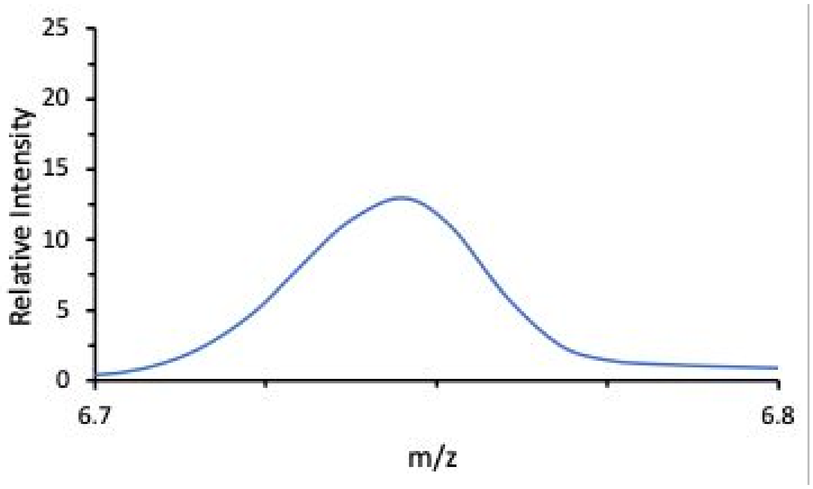

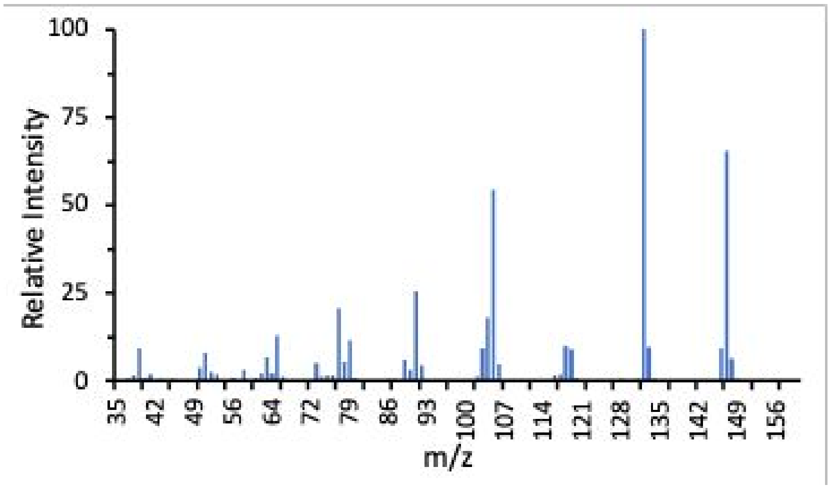

Figure S12: 1-(isocyanatomethyl)-4-methoxybenzene

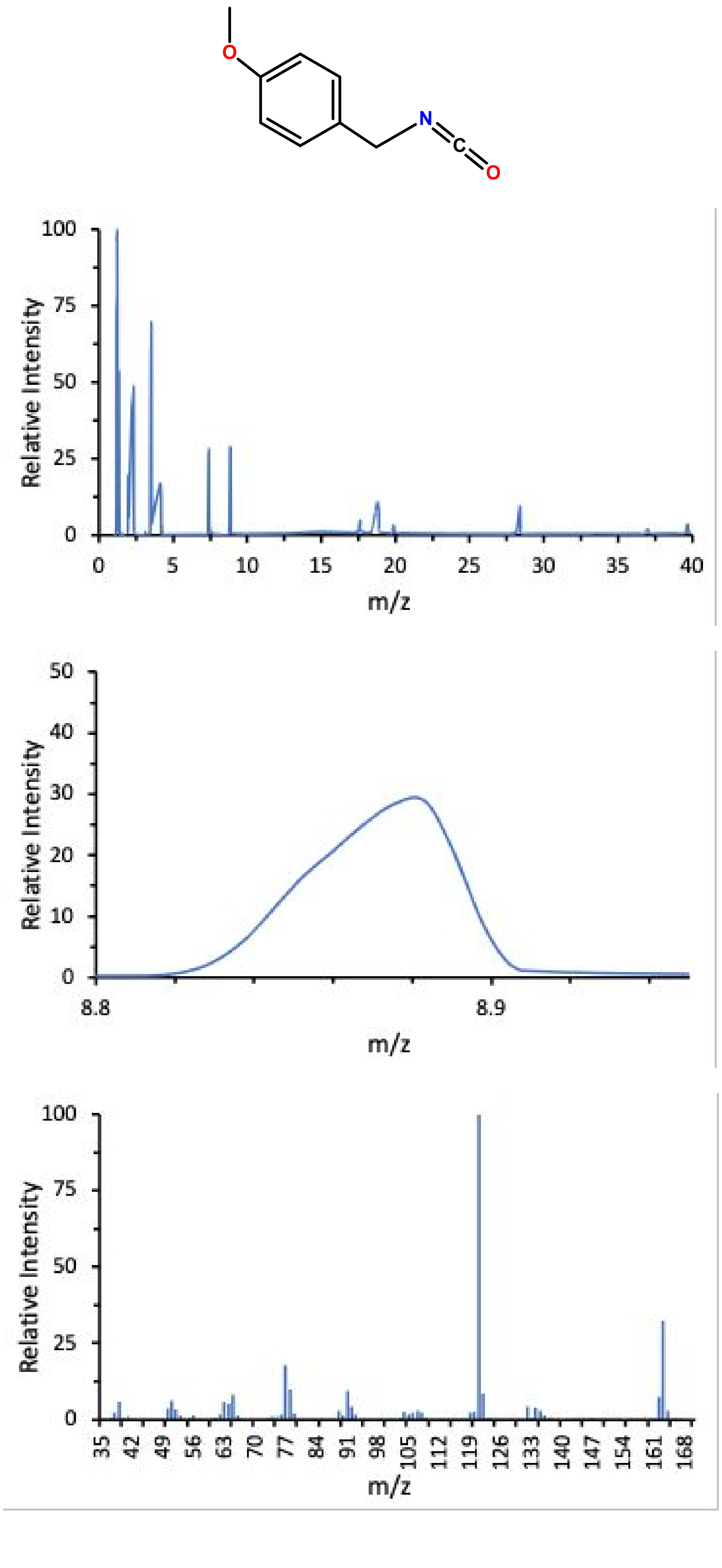

Figure S13: (3-isocyanatopropyl)benzene

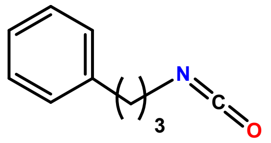

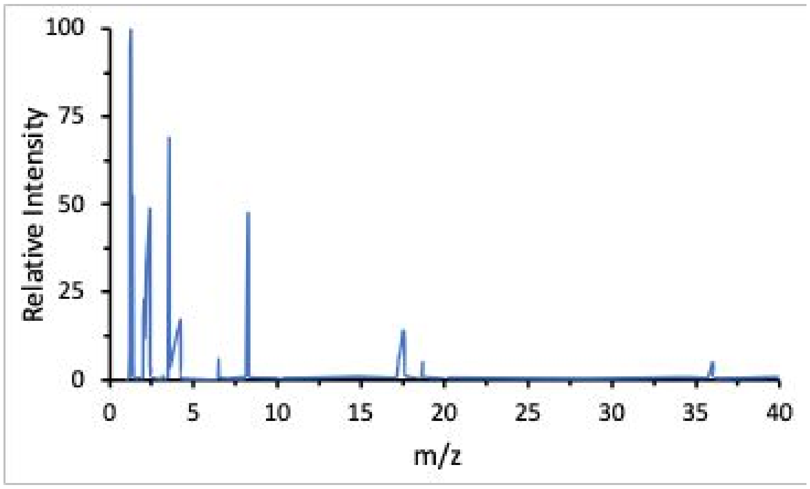

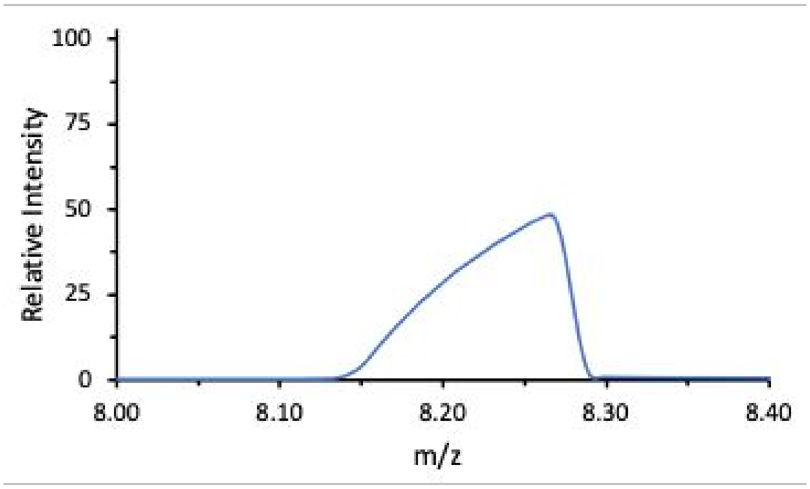

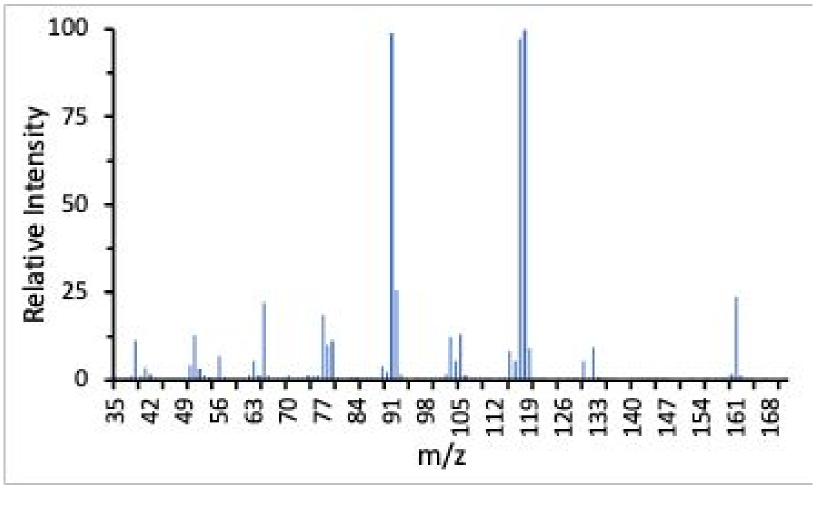

Figure S14: 1-chloro-4-(isocyanatomethyl)benzene

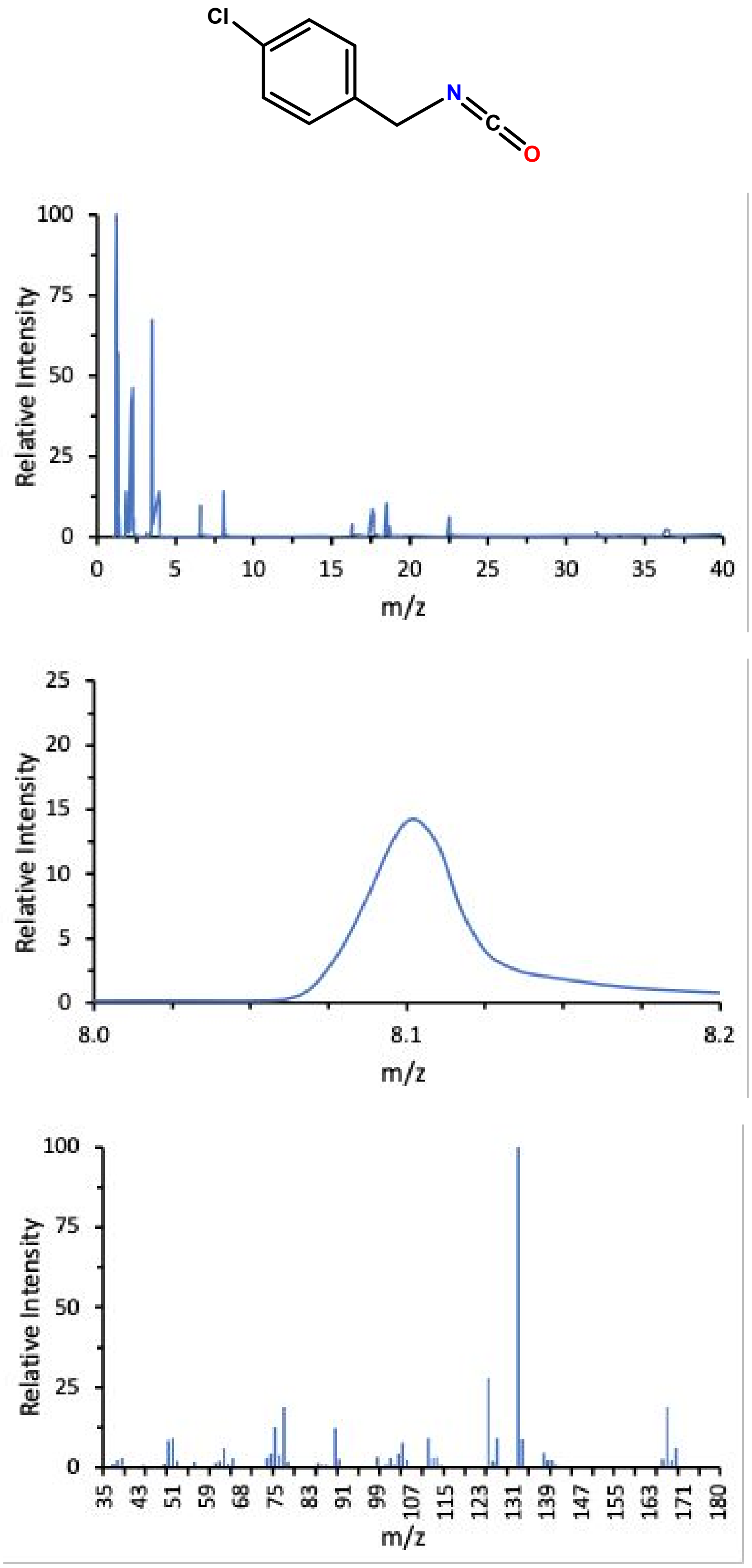

Figure S15: 1-fluoro-4-(isocyanatomethyl)benzene

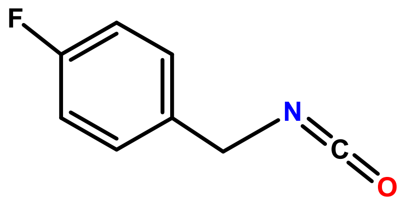

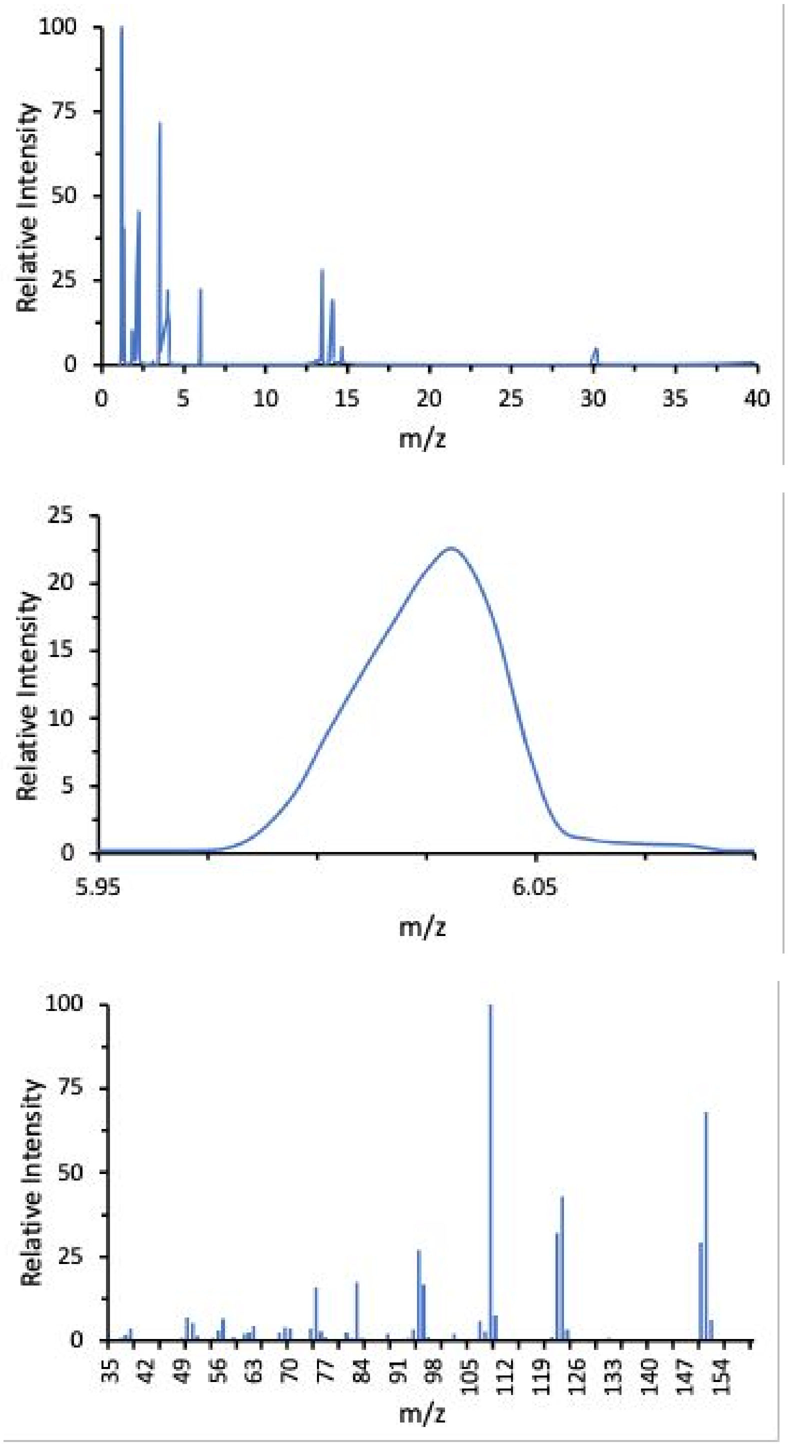

Figure S16: (1-isocyanatoethyl)benzene

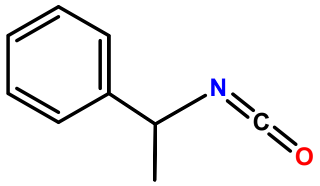

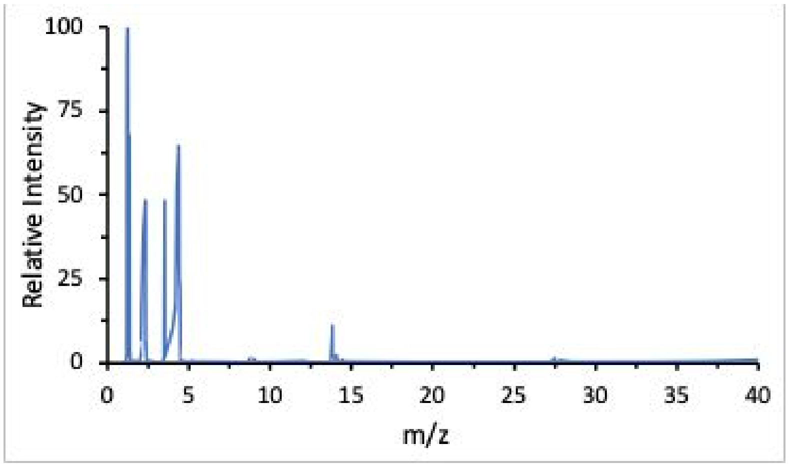

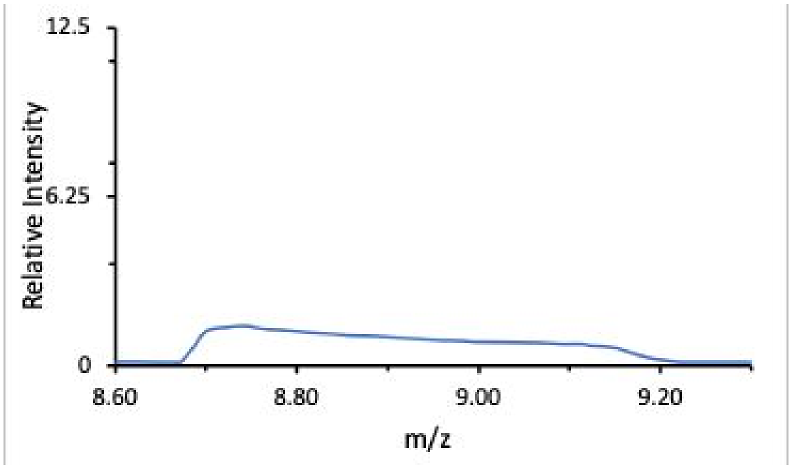

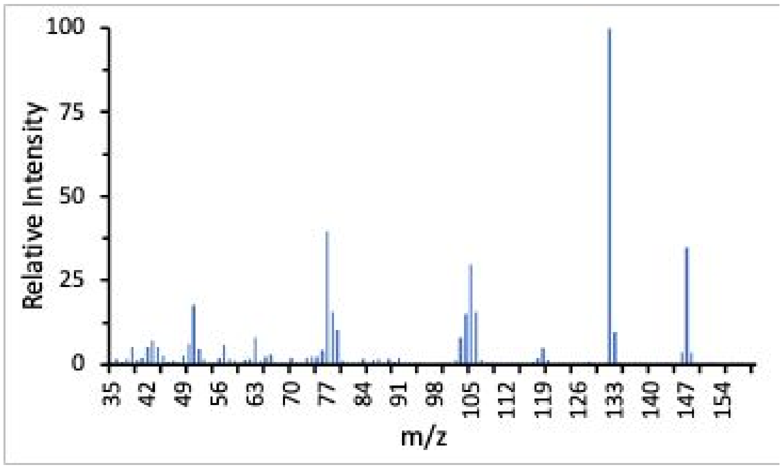

Figure S17: 1,3-dipropylurea

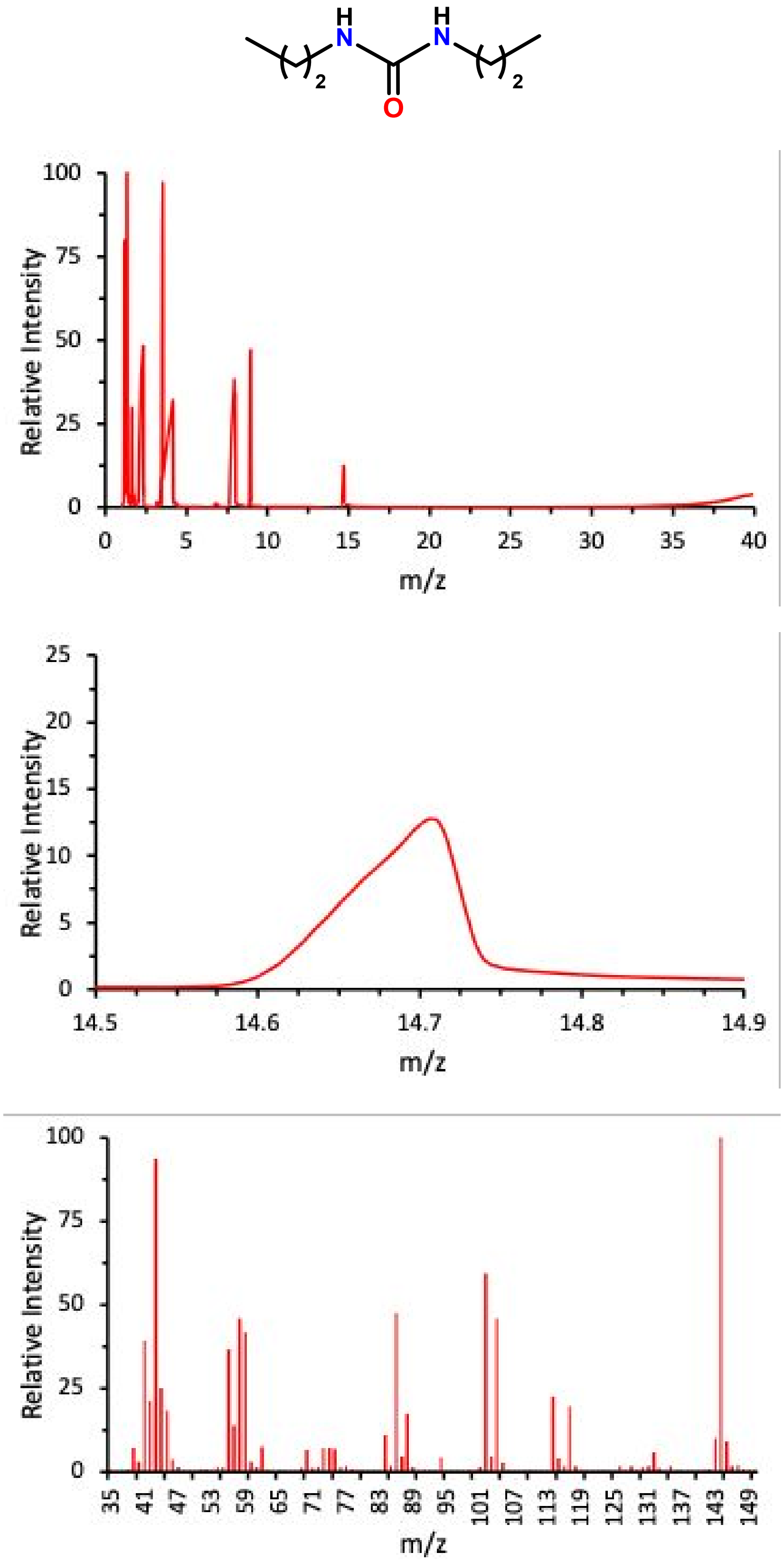

Figure S18: 1,3-dibutylurea

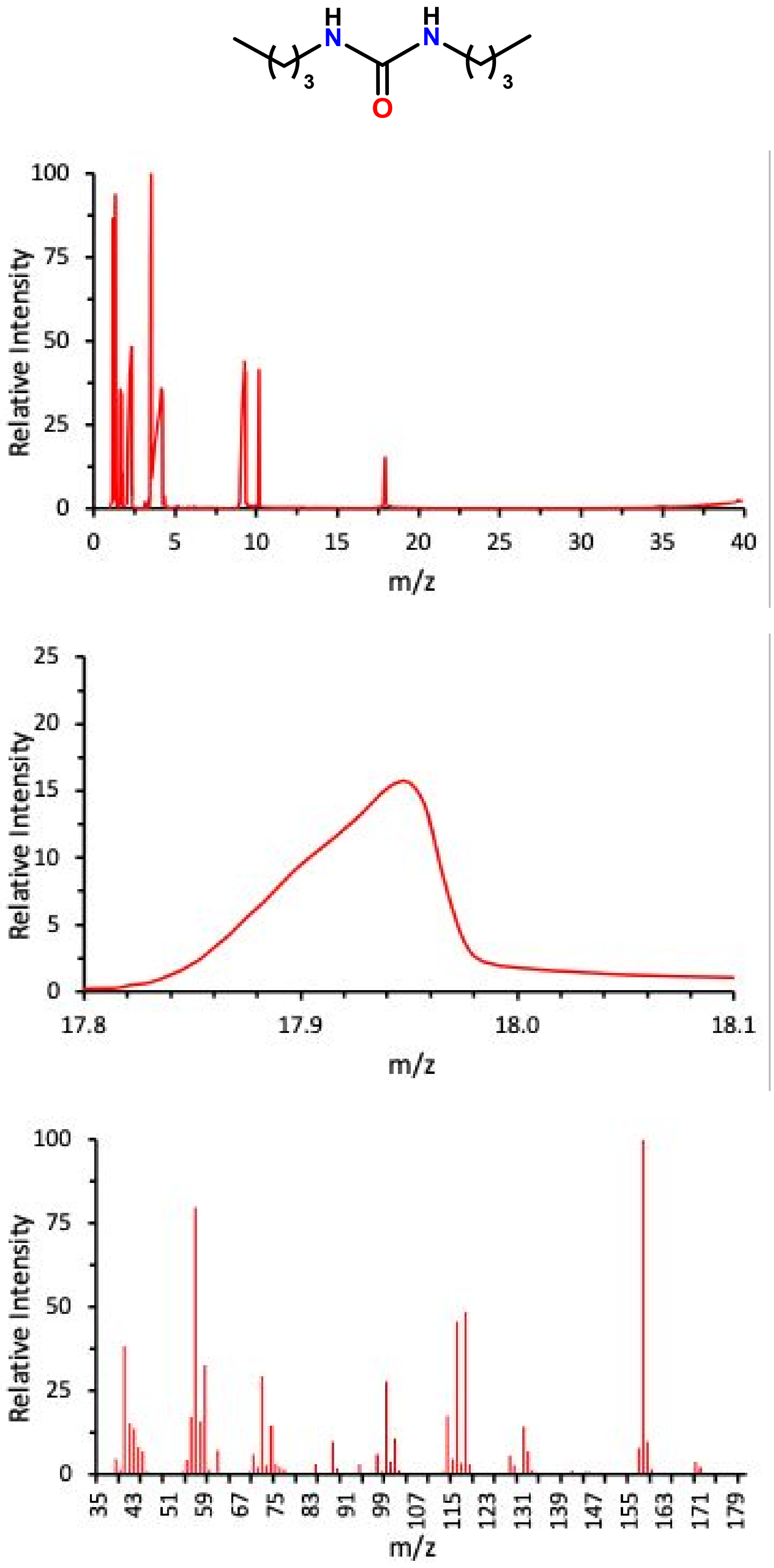

Figure S19: 1,3-dipentylurea

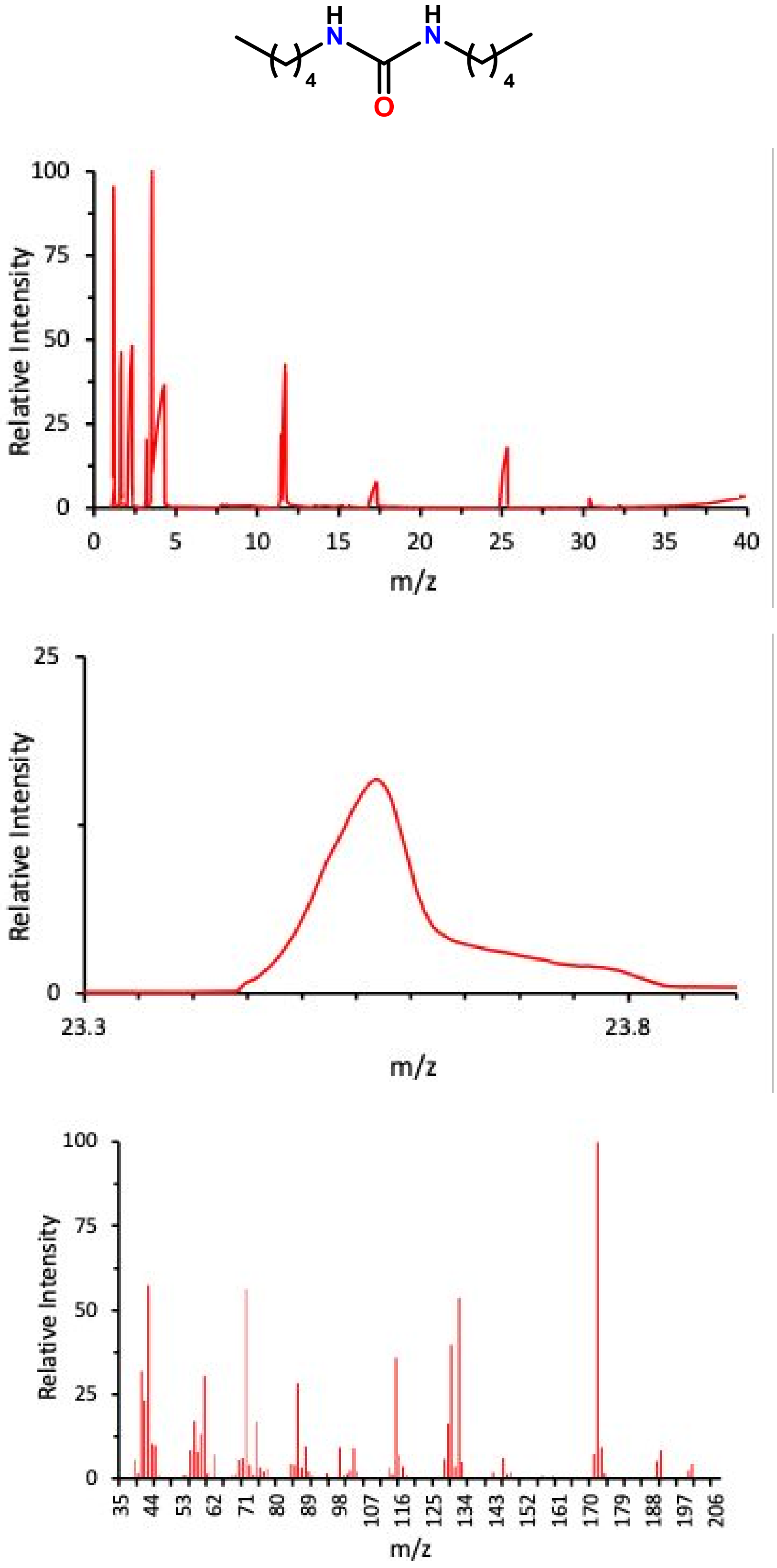

Figure S20: 1,3-dihexylurea

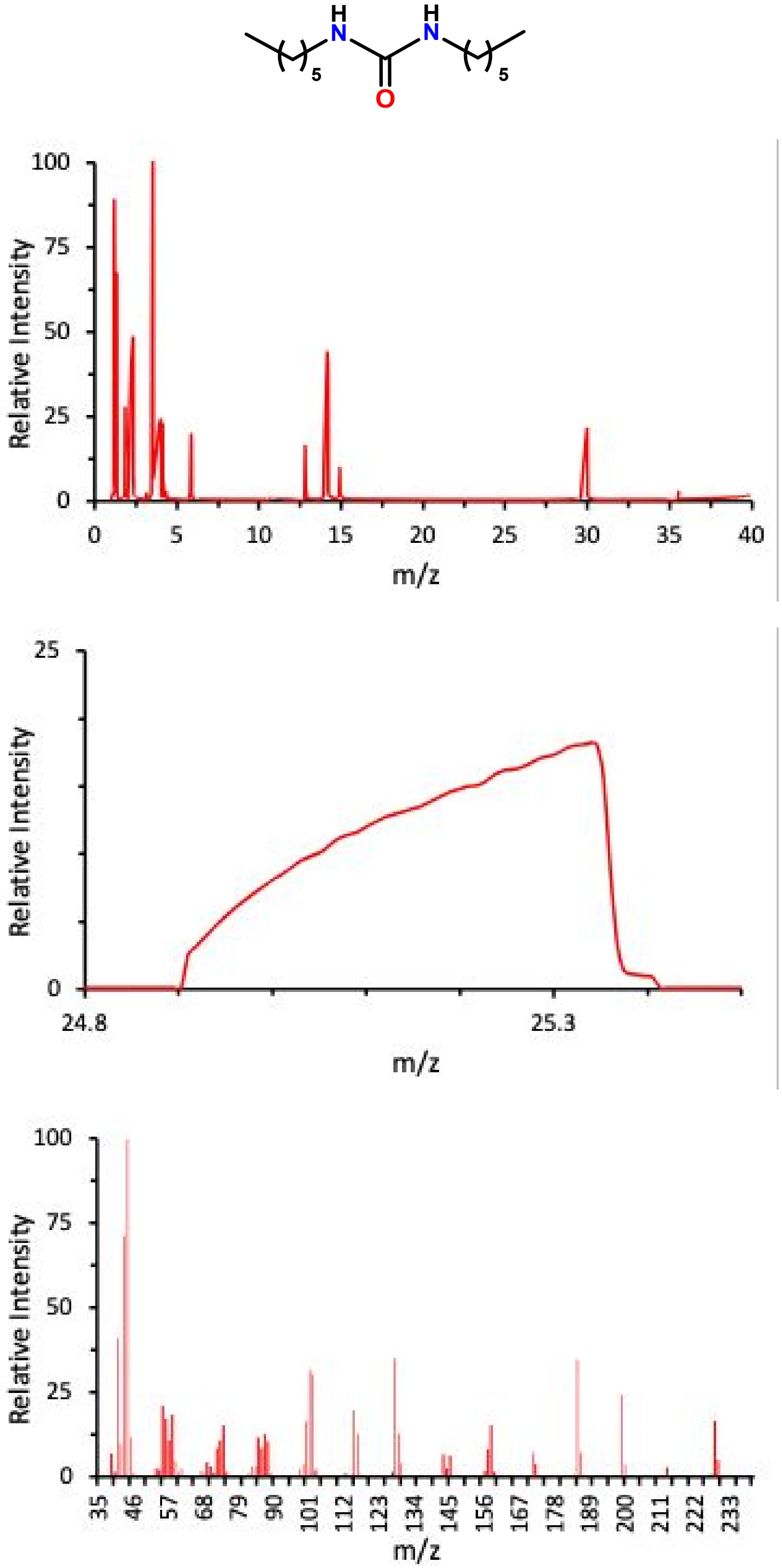

Figure S21: 1,3-diheptylurea

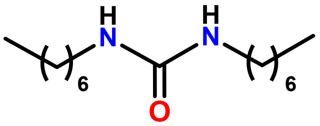

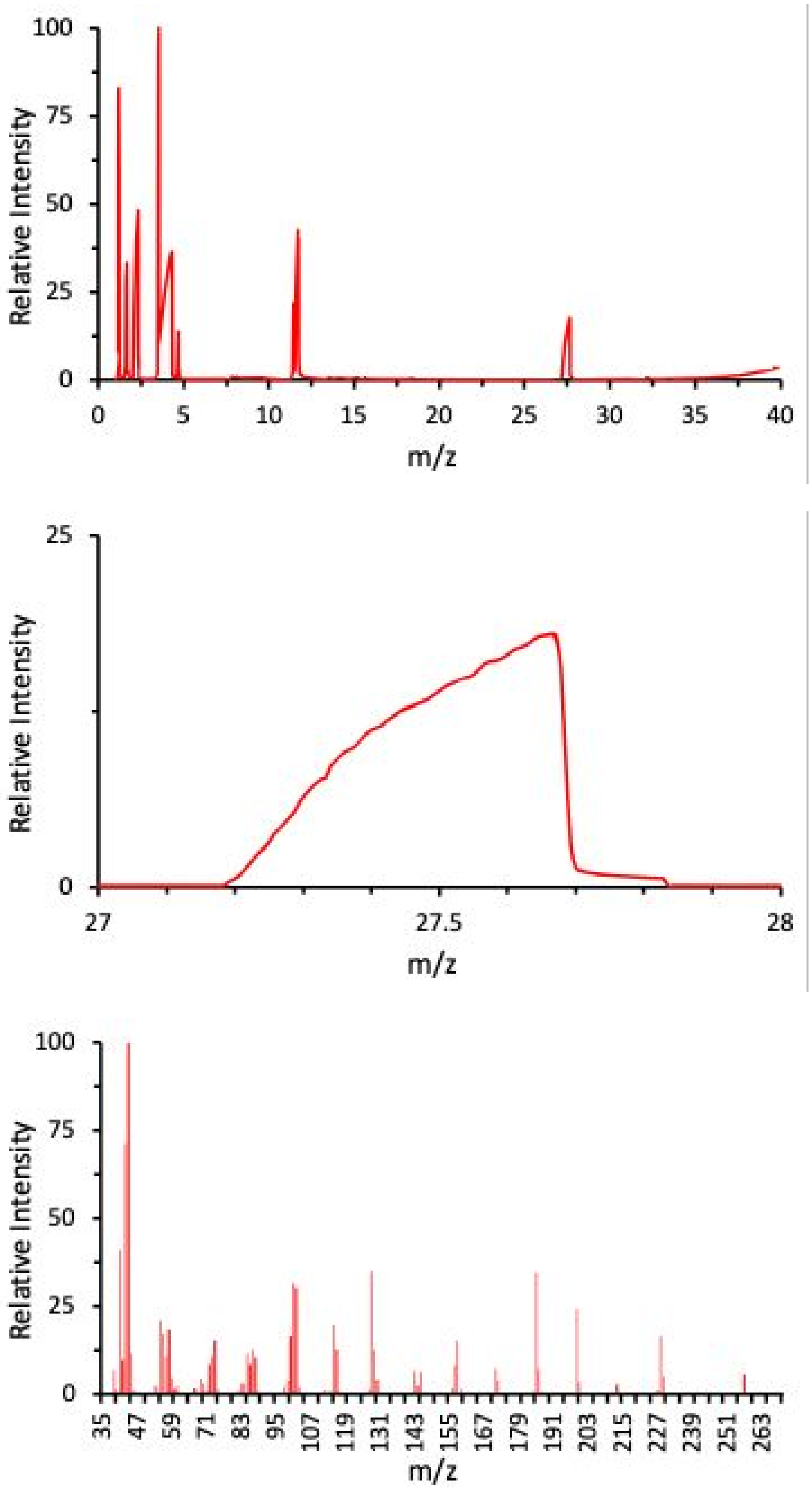

Figure S22: 1,3-dioctylurea

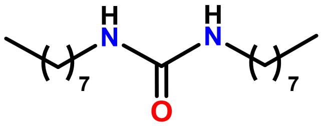

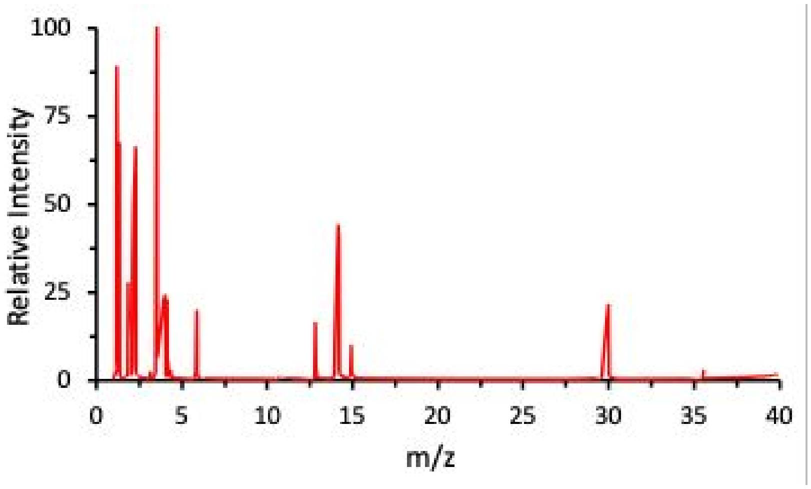

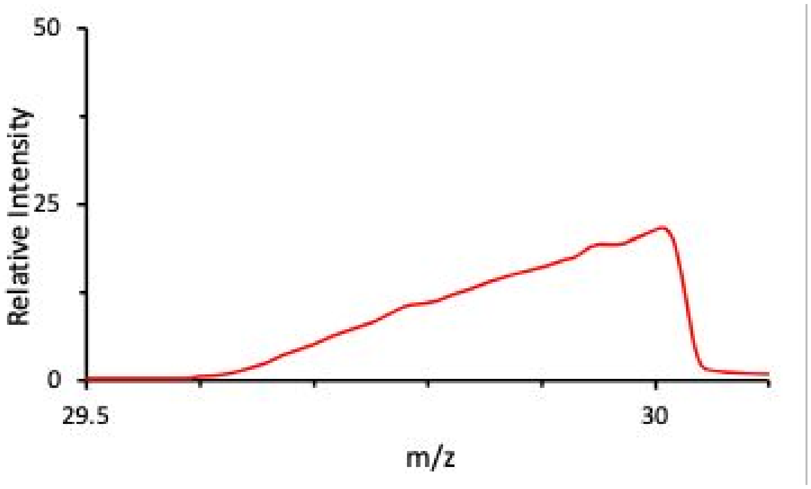

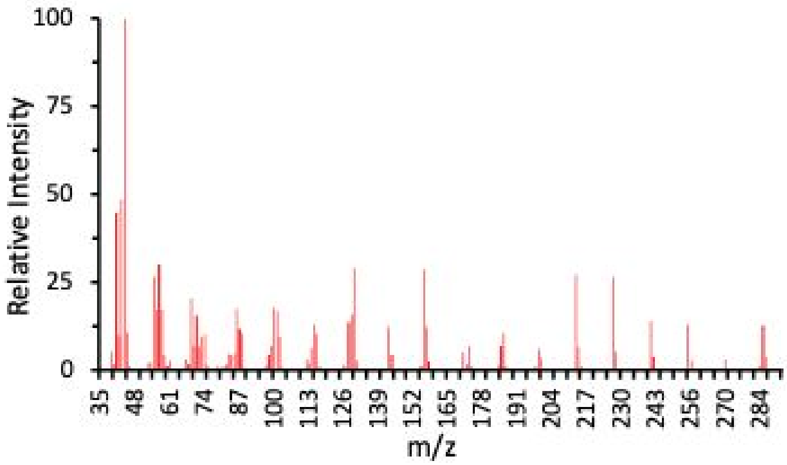

Figure S23: 1,3-dinonylurea

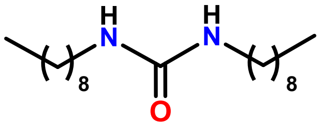

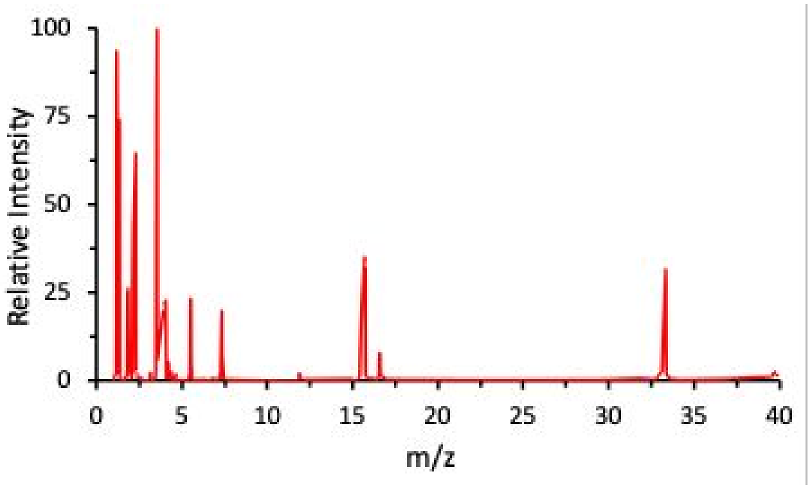

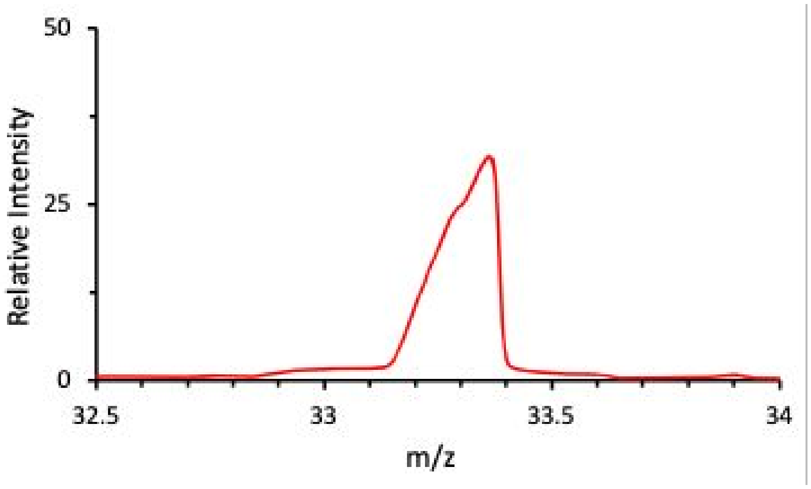

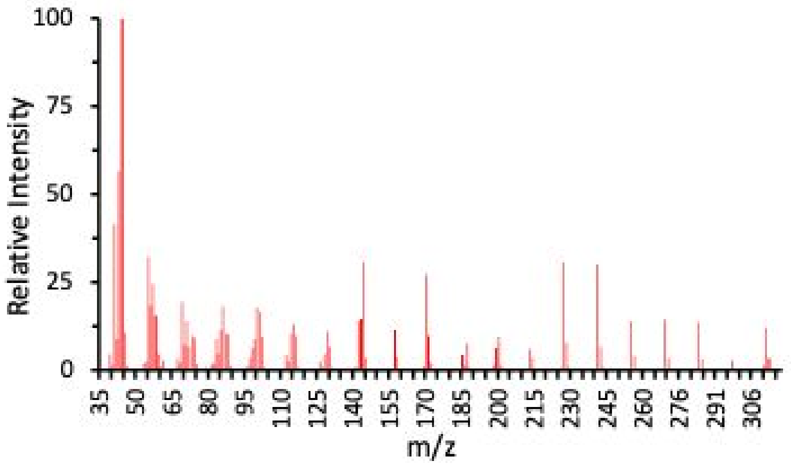

Figure S24: 1,3-didecylurea

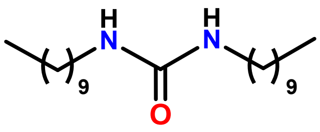

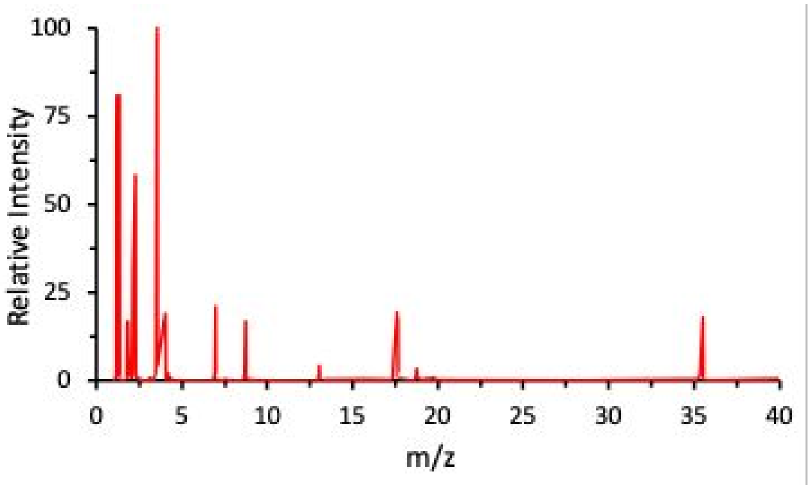

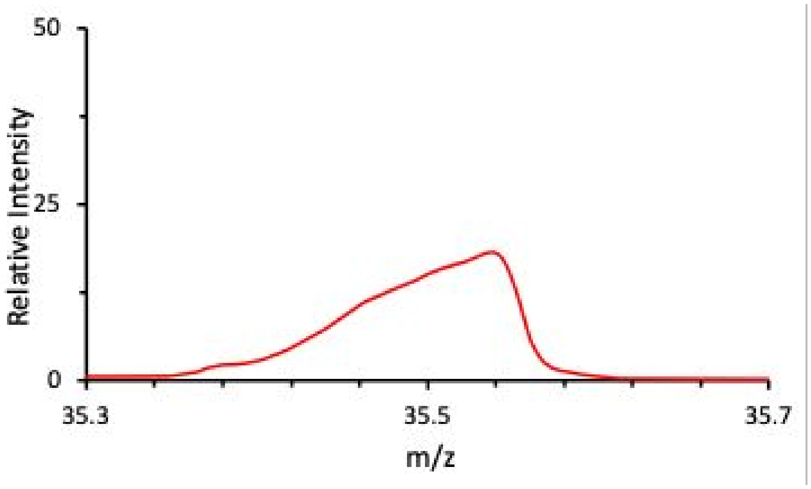

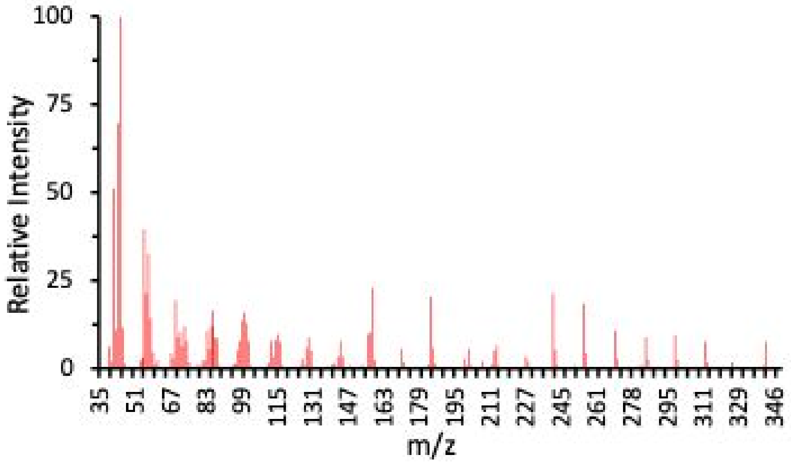

Figure S25: 1,3-dicyclohexylurea

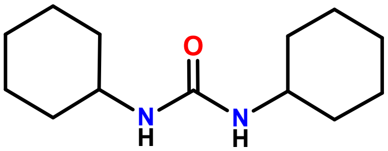

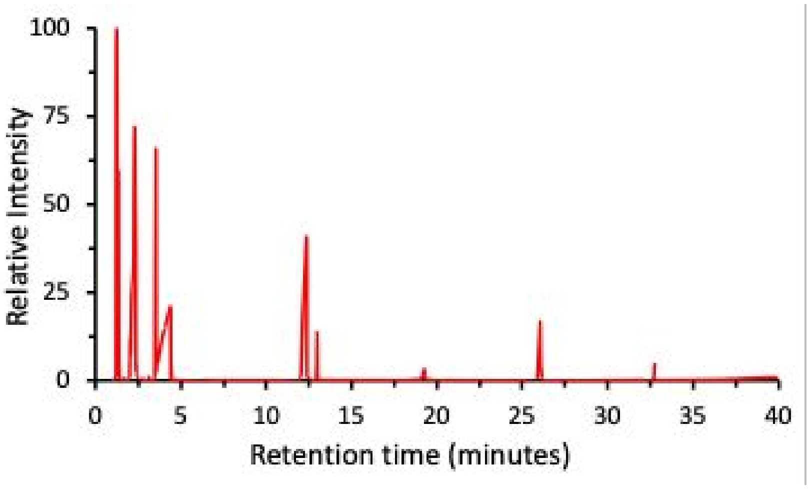

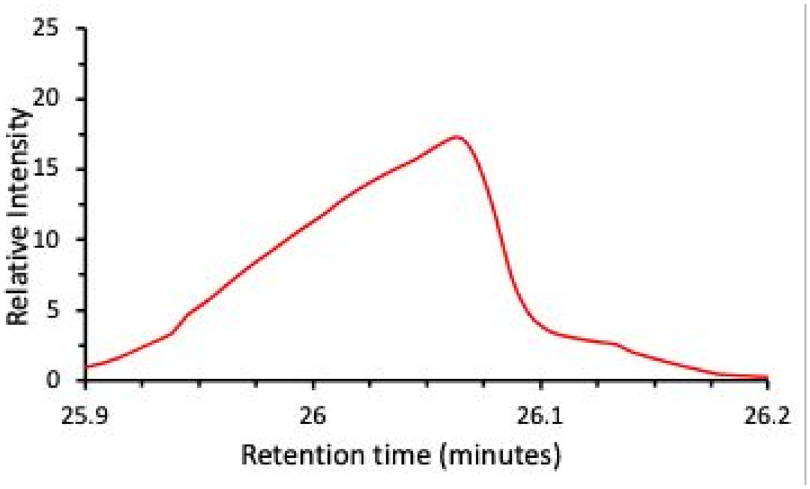

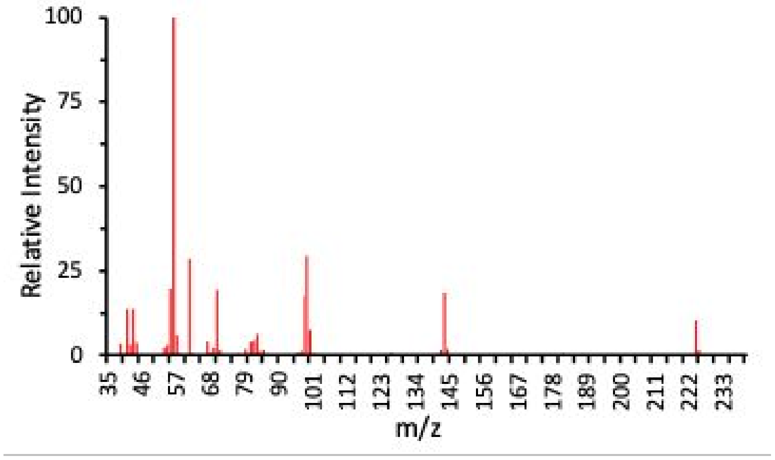

Figure S26: 1,3-dibenzylurea

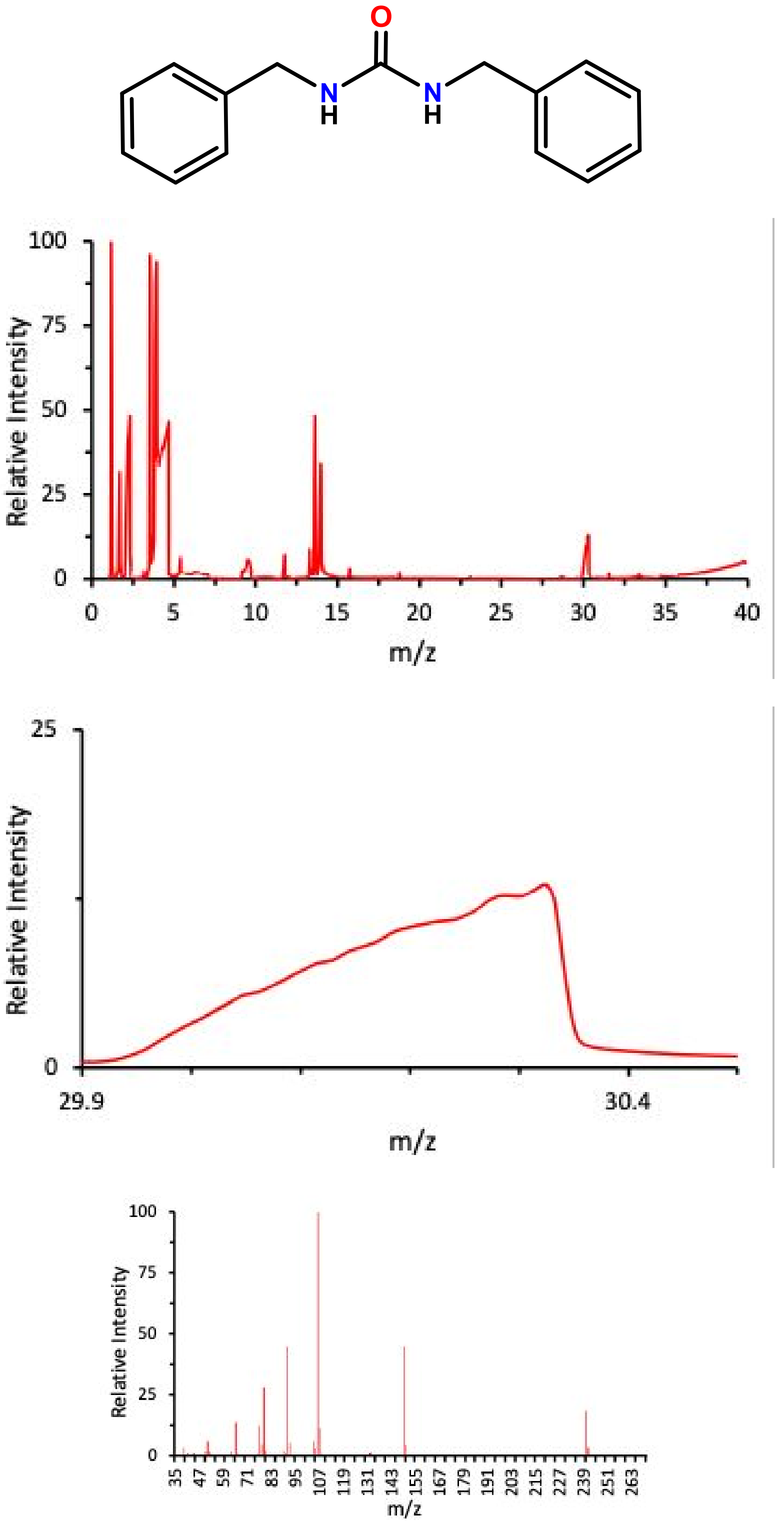

Figure S27: 1,3-bis(4-methylbenzyl)urea

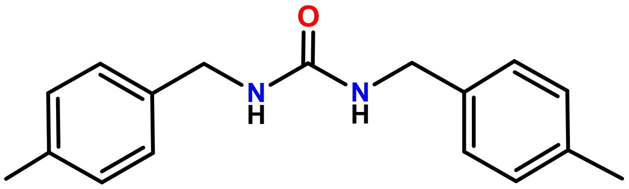

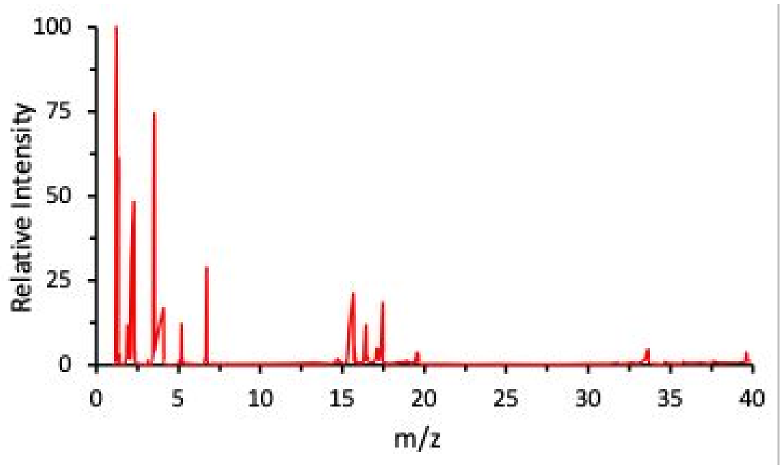

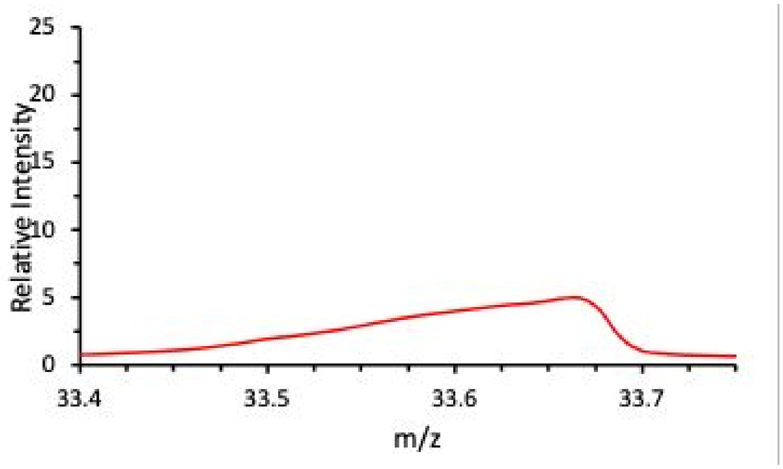

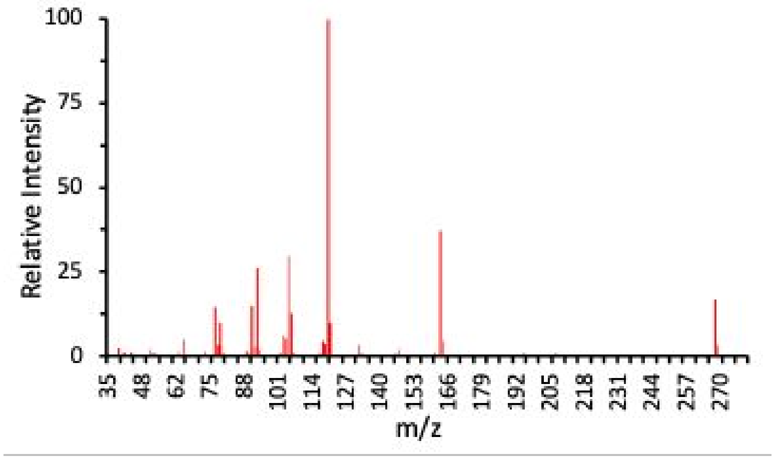

Figure S28: 1,3-bis(4-methoxybenzyl)urea

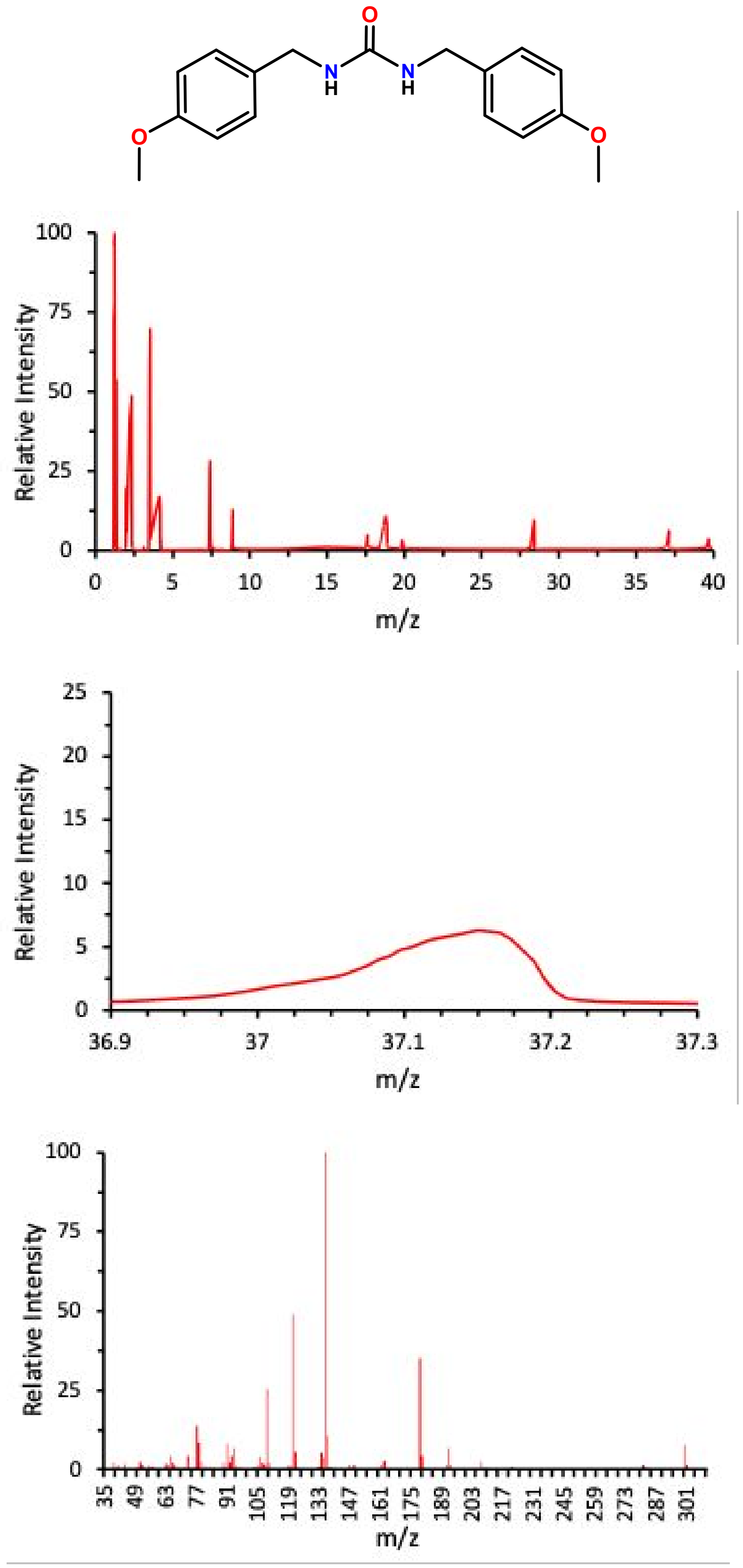

Figure S29: 1,3-diphenylpropylurea

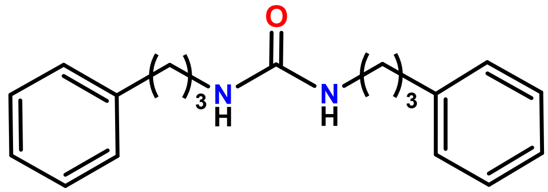

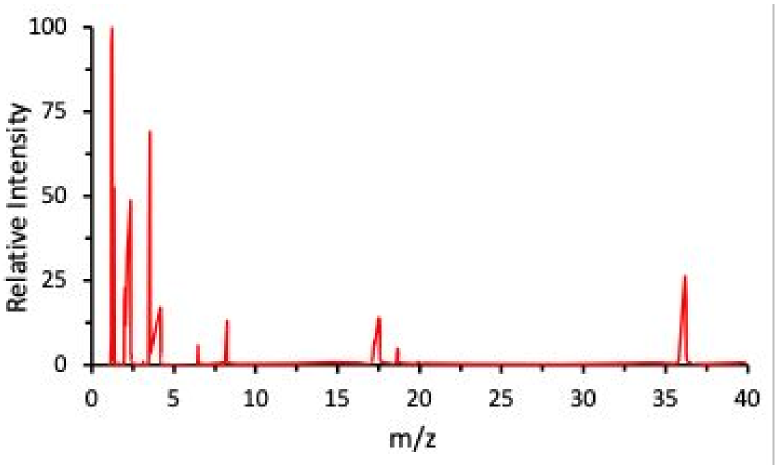

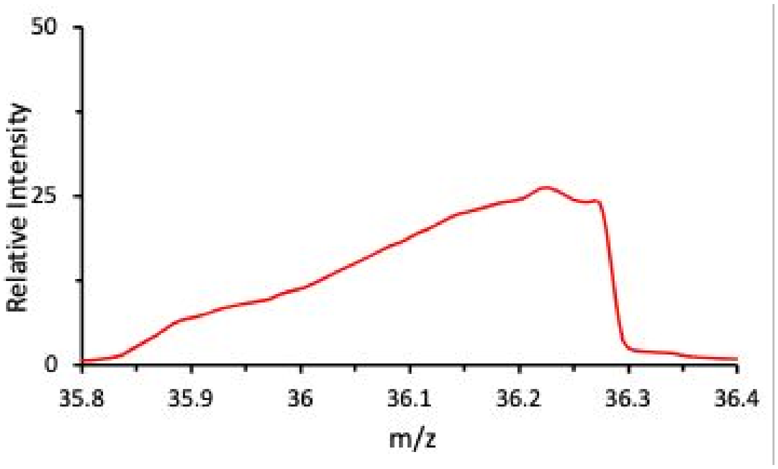

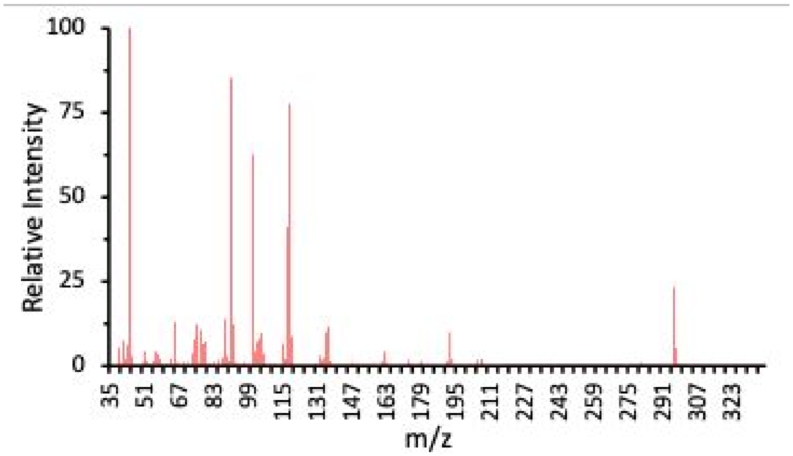

Figure S30: 1,3-bis(4-chlorobenzyl)urea

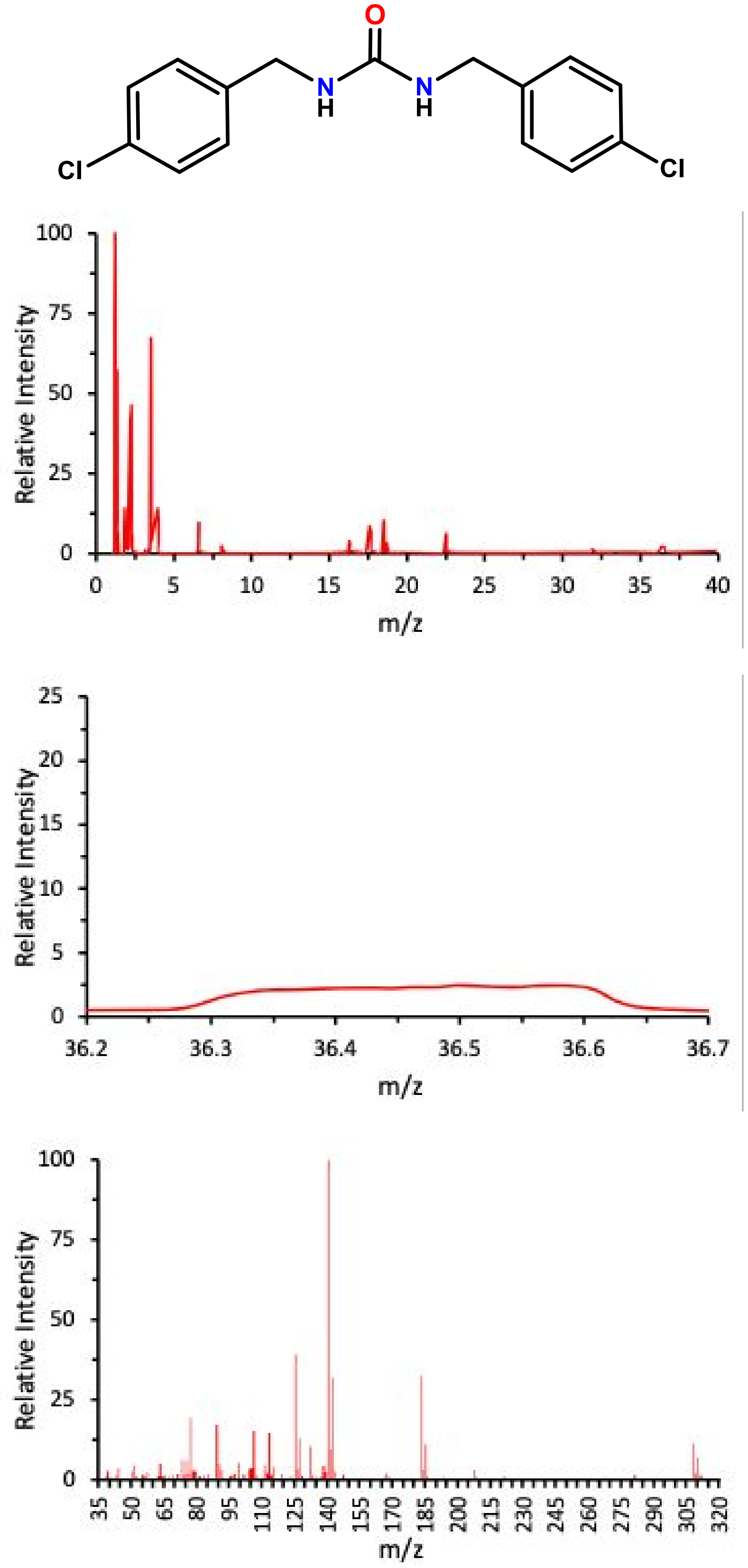

Figure S31: 1,3-bis(4-fluorobenzyl)urea

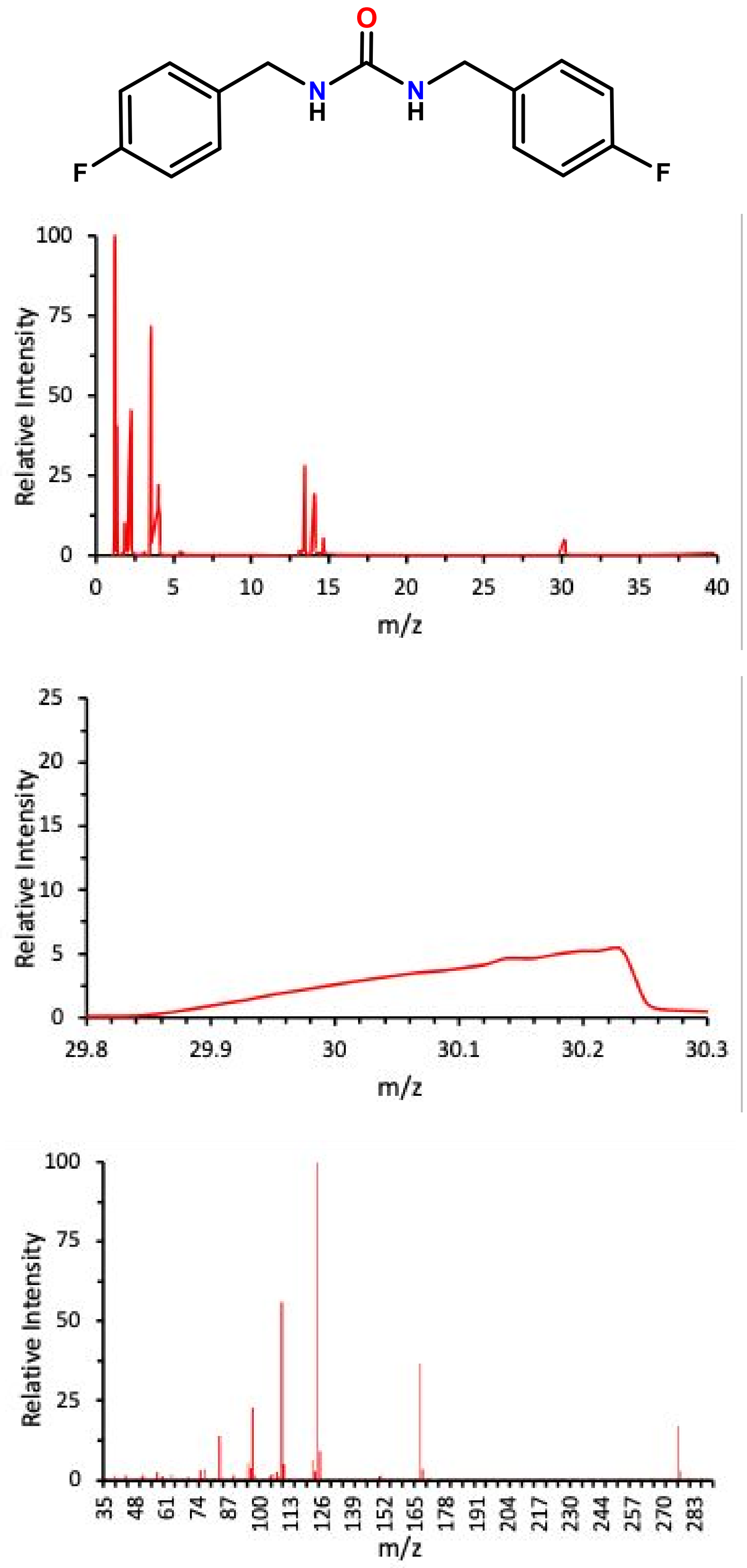

Figure S32: 1,3-bis(1-phenylethyl)urea

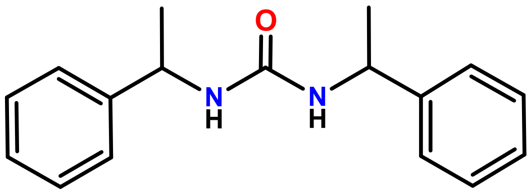

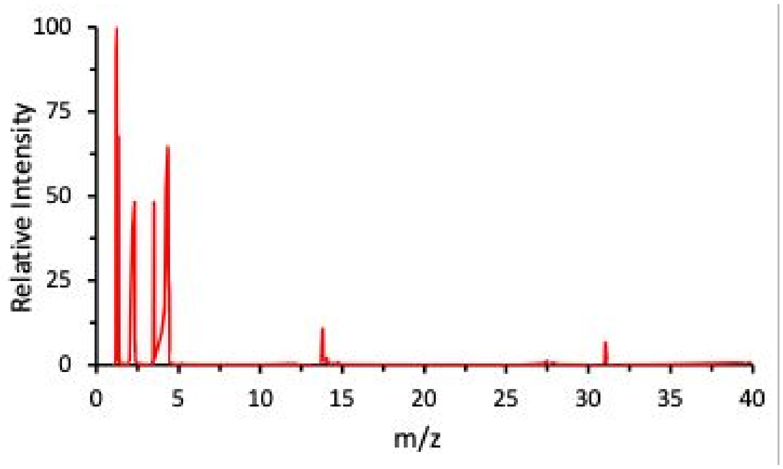

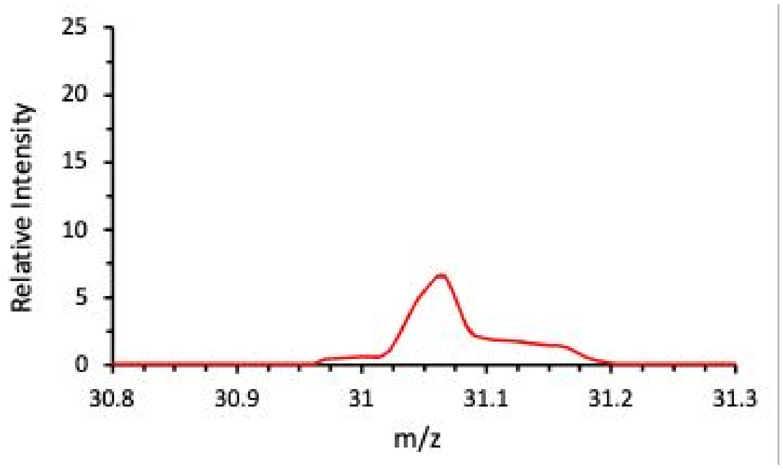

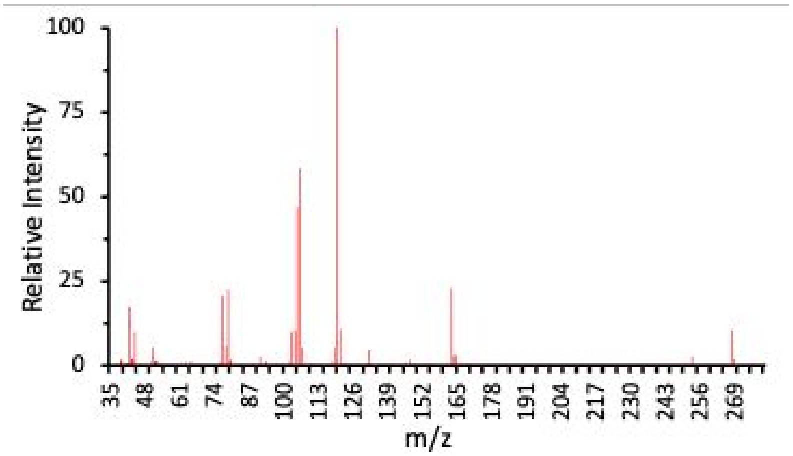

Other Instrumentation

Figure S33: FT-IR of NNN Ligand

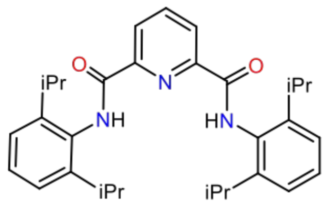

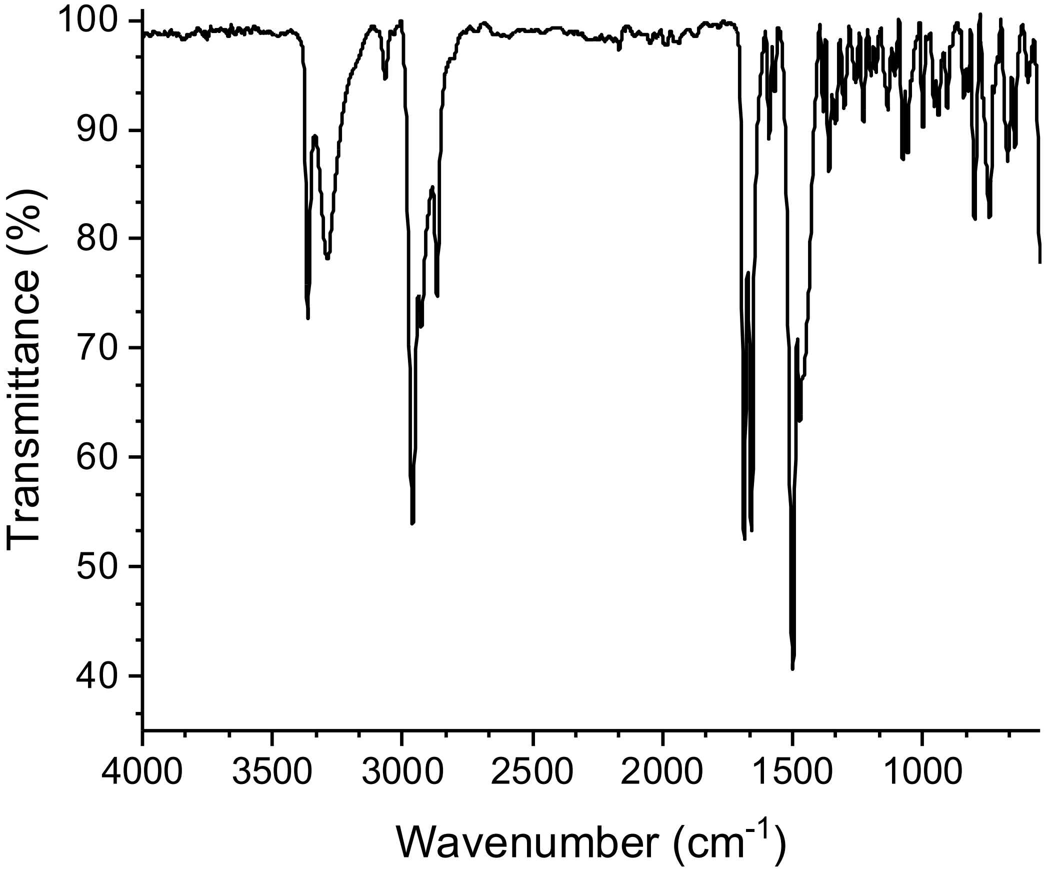

Figure S34: ^{1}H NMR of NNN Ligand

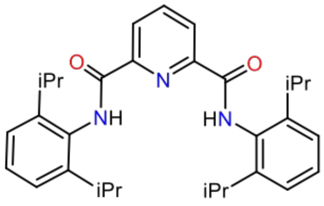

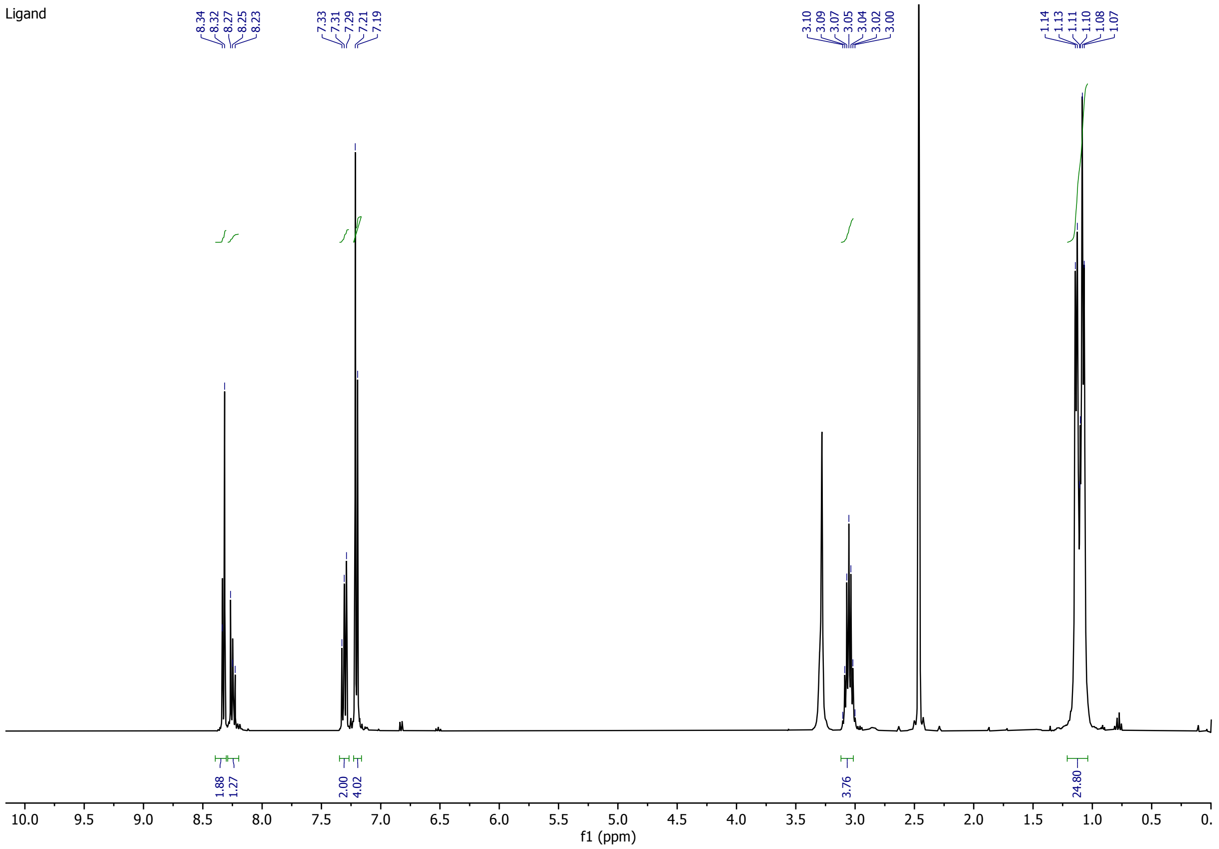

^{1}H NMR (400 MHz, DMSO-d_6) δ 8.33 (d, J = 7.8 Hz, 2H), 8.29 – 8.20 (m, 1H), 7.35 – 7.27 (m, 2H), 7.20 (d, J = 7.8 Hz, 4H), 3.06 (h, J = 6.9 Hz, 4H), 1.21 – 1.04 (m, 25H).

Figure S35: ^{13}C NMR of NNN Ligand

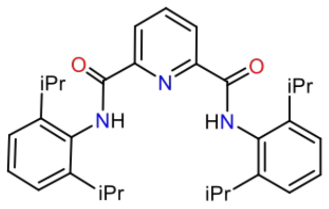

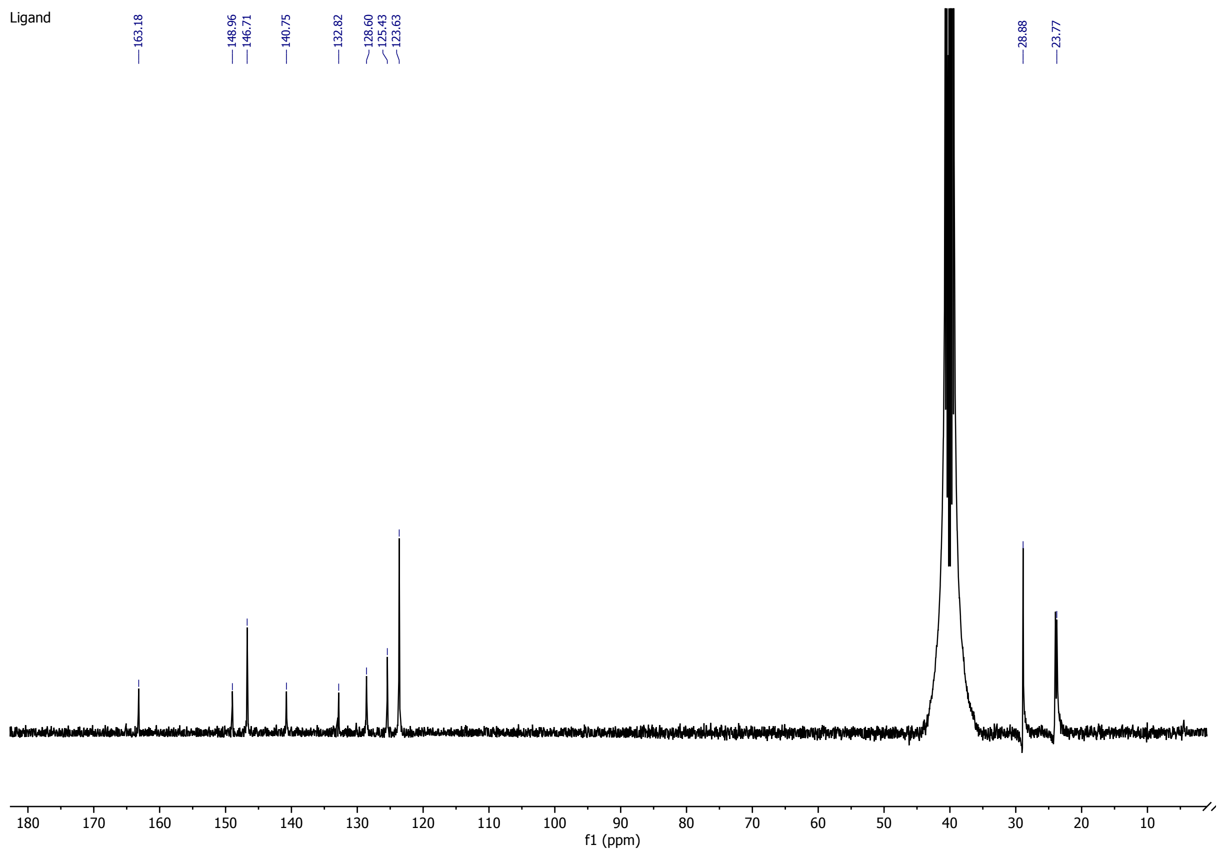

^{13}C NMR (101 MHz, DMSO-D_6) δ 163.18, 148.96, 146.71, 140.75 132.82, 128.60, 125.43, 123.63, 28.88, 23.77.

Figure S36: ESI-MS (negative mode) of nickel (II) complex

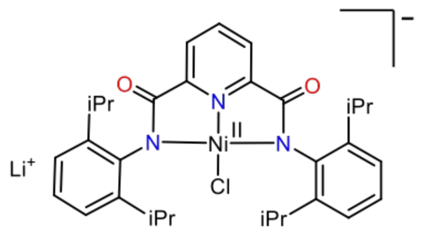

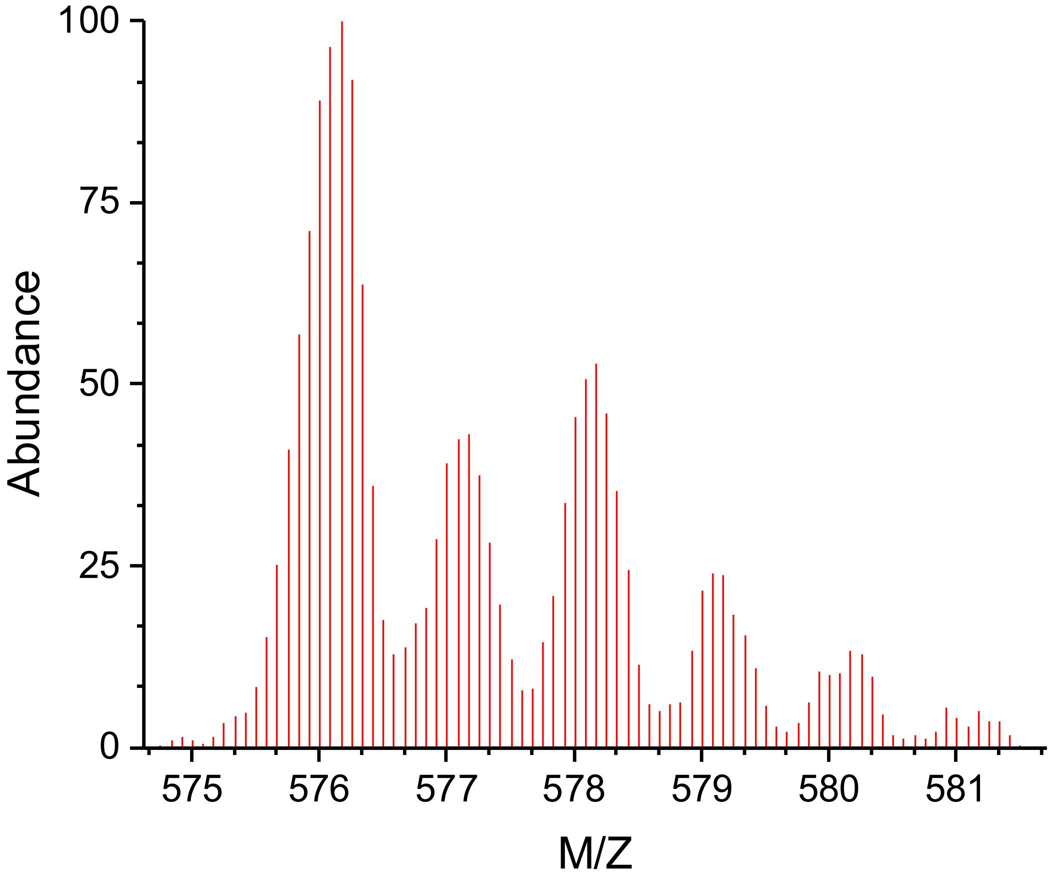

Figure S37: FT-IR of nickel (II) complex

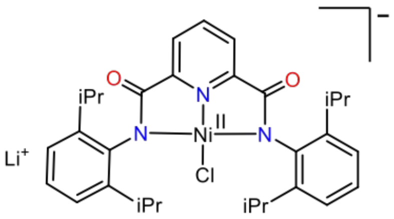

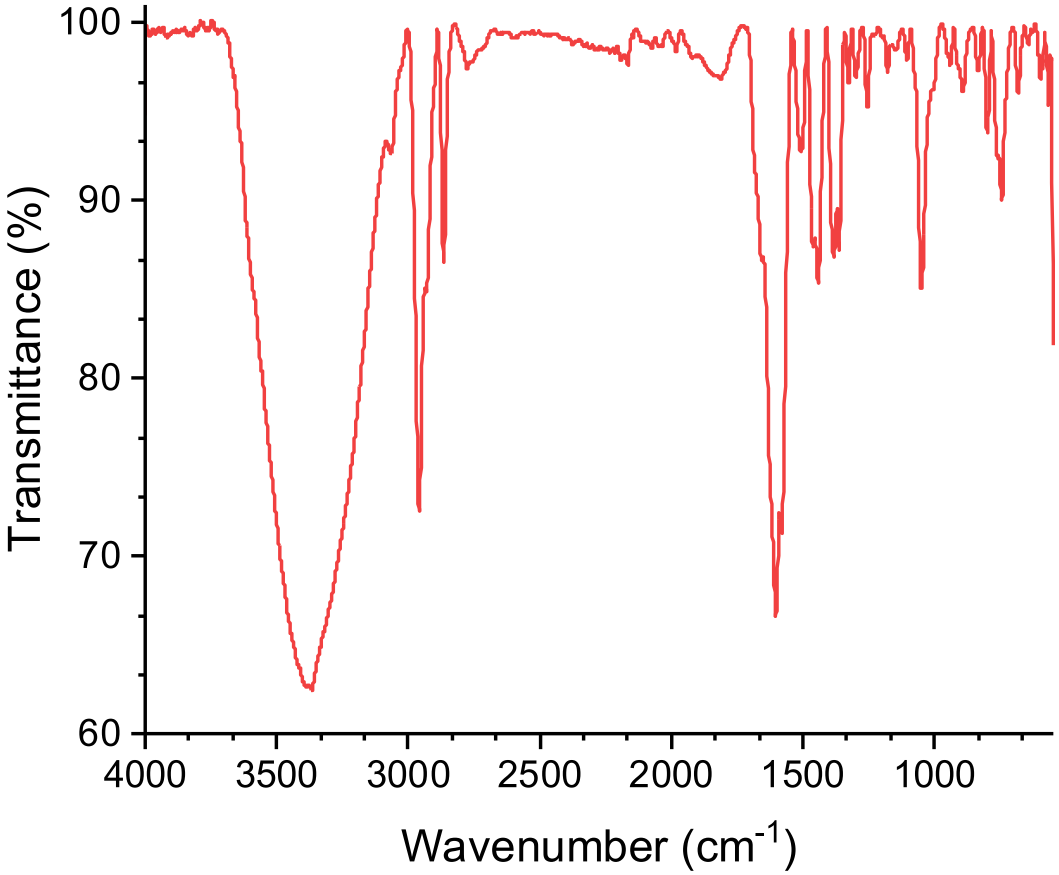

Figure S38: ^{1}H NMR of nickel (II) complex

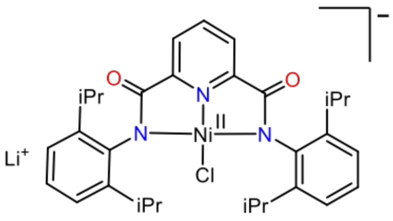

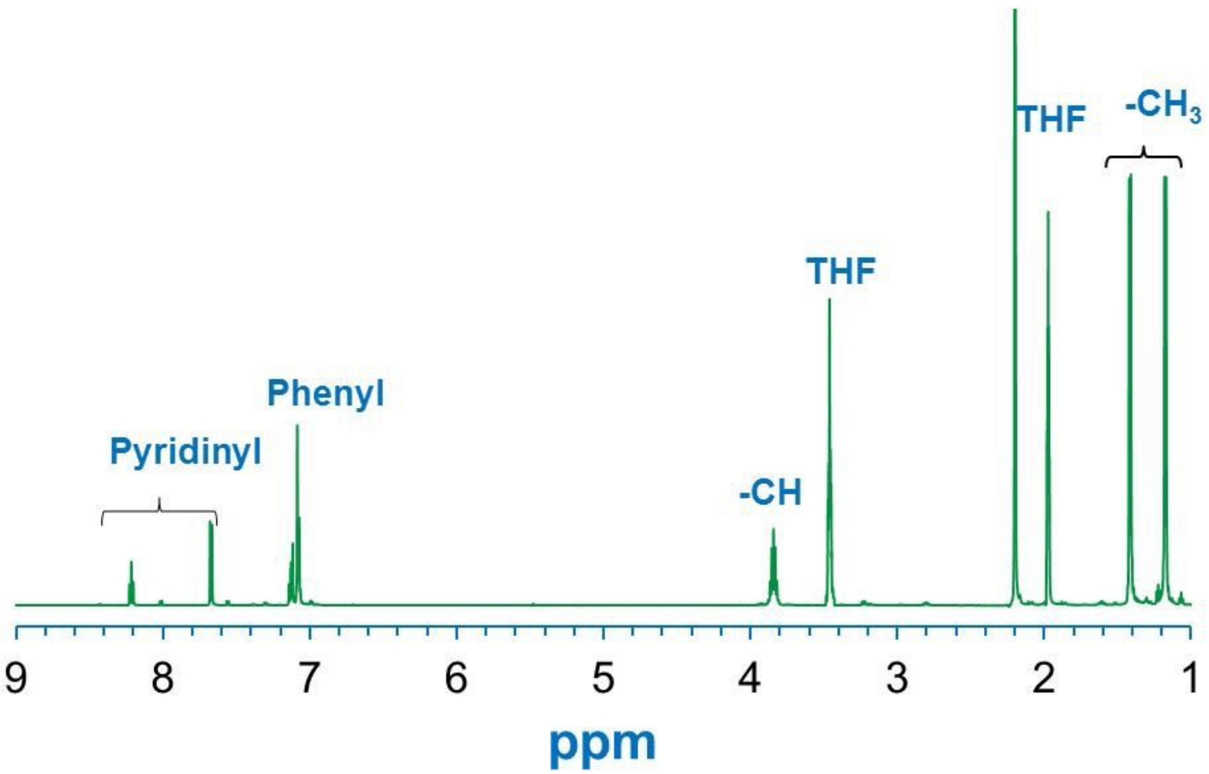

(^{1}H-NMR (600 MHz, 298 K, CD_3CN, δ)

Figure S39: ^{1}H NMR of 1-3-diheptyl urea

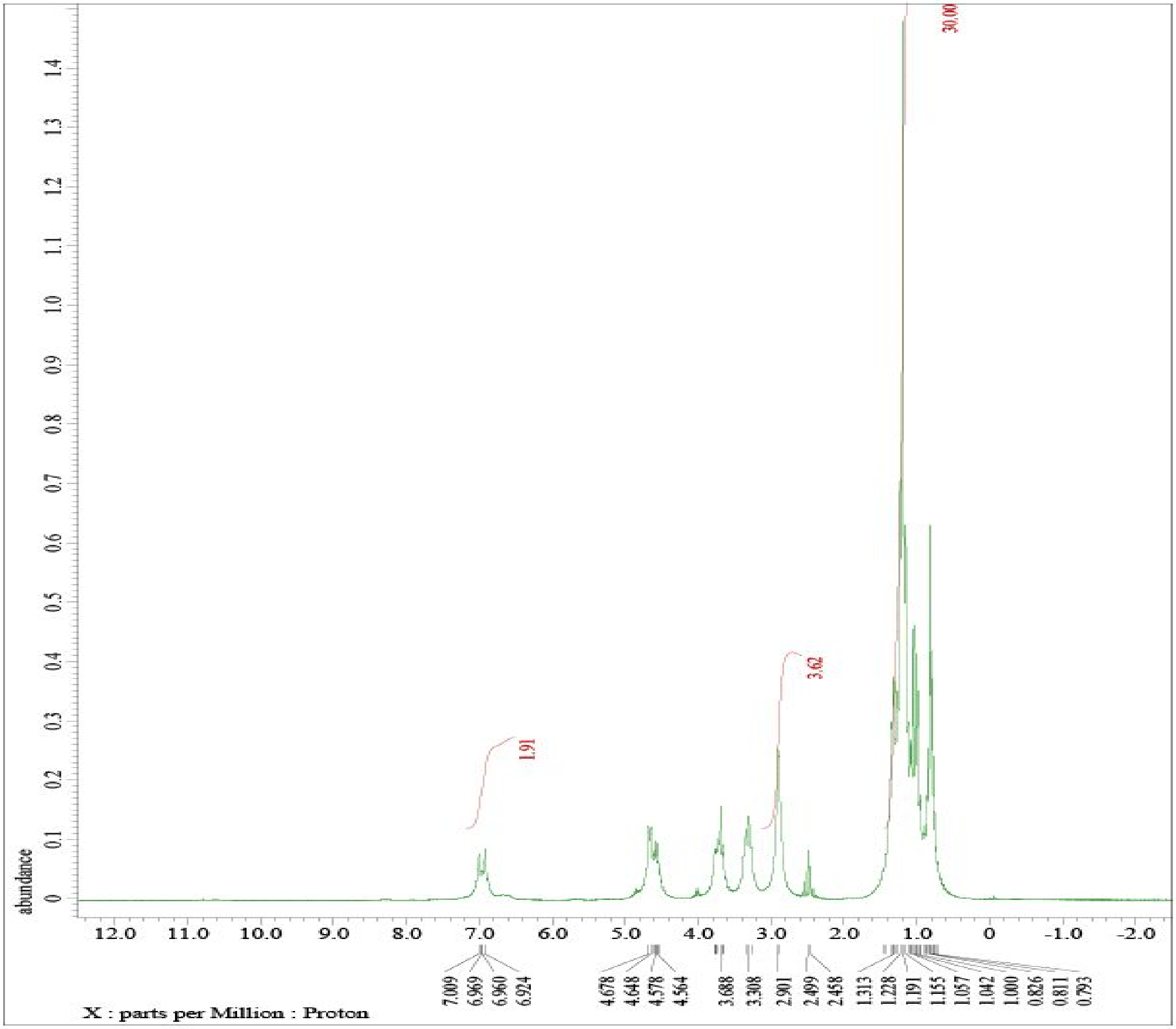

^{1}H-NMR (400 MHz, DMSO-D6) δ 7.01-6.92 (m, 2H), 2.89 (d, J = 5.5 Hz, 4H), 1.42-0.72 (m, 30H)

Figure S40: ^{13}C NMR of 1-3-diheptyl urea

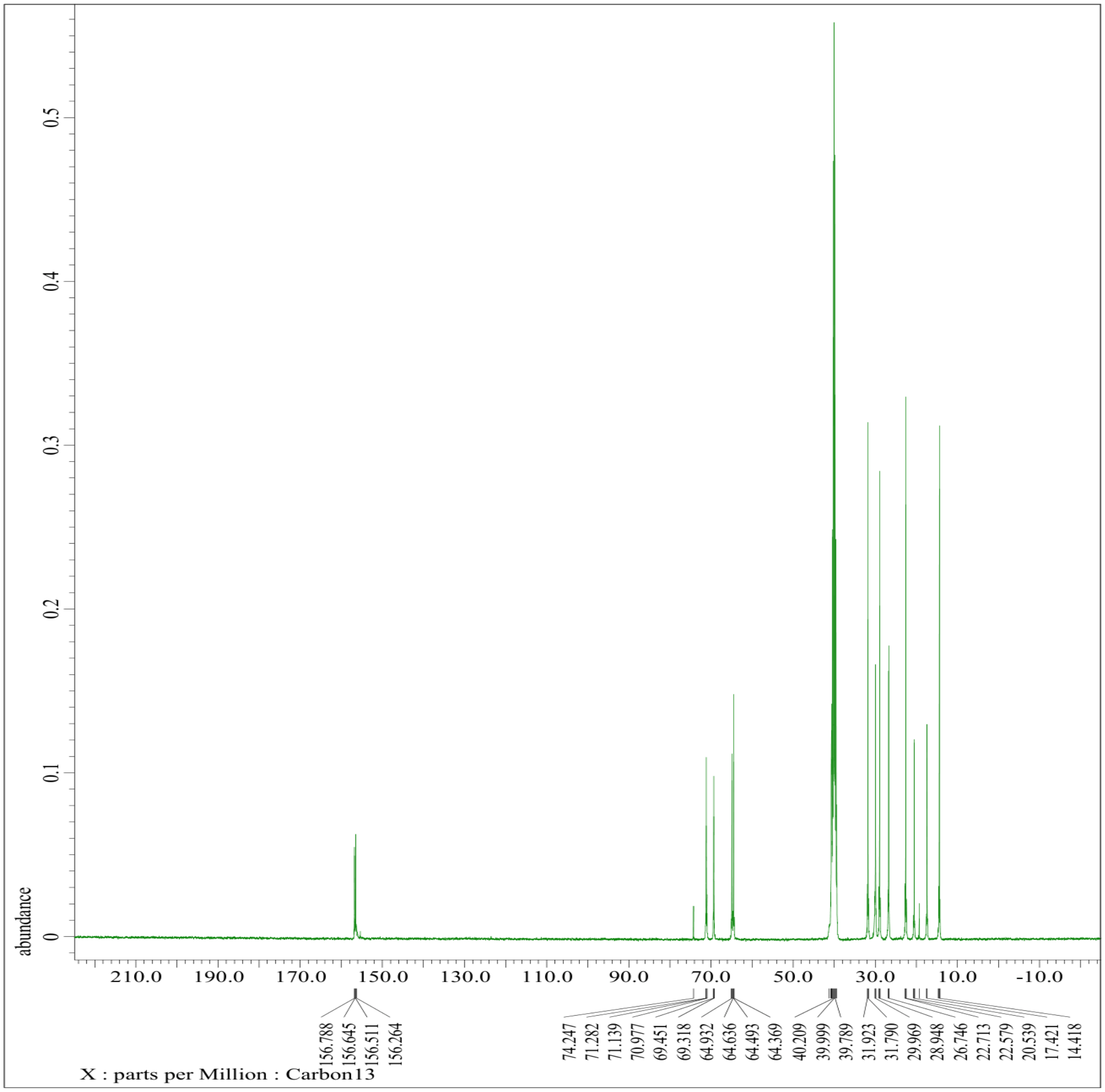

Figure S41: APCI mass spectrum (**A**) of the acyl alkoxy nickel(III) intermediate (**C**) as an sodium ion adduct; Inset Figure **B** is the theoretical isotope distribution calculation of the intermediate, **C**

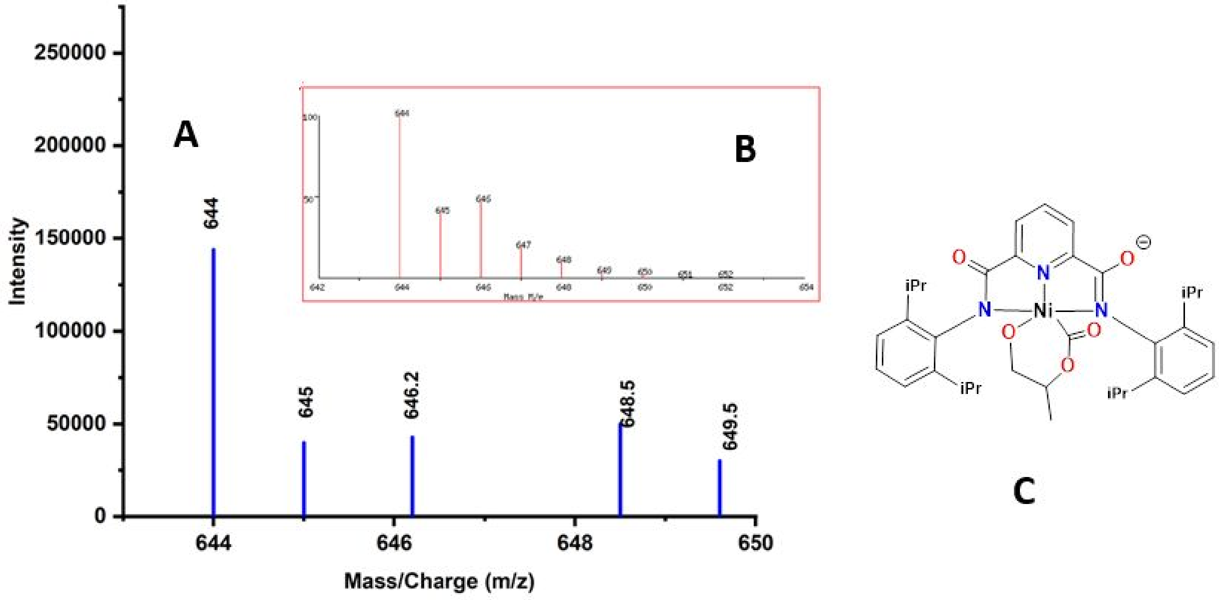

Supplement: Supplementary file 1 [file ao6c01401_si_001.pdf]
